# Study of Structure–Activity Relationships of the Marine Alkaloid Fascaplysin and Its Derivatives as Potent Anticancer Agents

**DOI:** 10.3390/md20030185

**Published:** 2022-03-02

**Authors:** Maxim E. Zhidkov, Moritz Kaune, Alexey V. Kantemirov, Polina A. Smirnova, Pavel V. Spirin, Maria A. Sidorova, Sergey A. Stadnik, Elena Y. Shyrokova, Dmitry N. Kaluzhny, Oleg A. Tryapkin, Tobias Busenbender, Jessica Hauschild, Tina Rohlfing, Vladimir S. Prassolov, Carsten Bokemeyer, Markus Graefen, Gunhild von Amsberg, Sergey A. Dyshlovoy

**Affiliations:** 1 Department of Chemistry and Materials, Institute of High Technologies and Advanced Materials, FEFU Campus, Far Eastern Federal University, Ajax Bay 10, Russky Island, 690922 Vladivostok, Russia; kantemirov_av@dvfu.ru (A.V.K.); smirnova_pa@dvfu.ru (P.A.S.); sidorova_ma@dvfu.ru (M.A.S.); pollianna_95@mail.ru (S.A.S.); triapkin_oa@dvfu.ru (O.A.T.); 2 Department of Oncology, Hematology and Bone Marrow Transplantation with Section Pneumology, Hubertus Wald-Tumorzentrum, University Medical Center Hamburg-Eppendorf, Martinistrasse 52, 20246 Hamburg, Germany; m.kaune@uke.de (M.K.); tobias.busenbender@gmx.de (T.B.); j.hauschild@uke.de (J.H.); trohlfing@uke.de (T.R.); c.bokemeyer@uke.de (C.B.); g.von-amsberg@uke.de (G.v.A.); 3 Department of Cancer Cell Biology, Engelhardt Institute of Molecular Biology, Russian Academy of Sciences, Vavilova 32, 119991 Moscow, Russia; spirin.pvl@gmail.com (P.V.S.); elena.j.shirokova@phystech.edu (E.Y.S.); prassolov@mail.ru (V.S.P.); 4 Center for Precision Genome Editing and Genetic Technologies for Biomedicine, Engelhardt Institute of Molecular Biology, Russian Academy of Sciences, Vavilova 32, 119991 Moscow, Russia; 5 Moscow Institute of Physics and Technology, National Research University, Institutskiy Per. 9, 141701 Dolgoprudny, Russia; 6 Laboratory of DNA Protein Interaction, Engelhardt Institute of Molecular Biology, Russian Academy of Sciences, Vavilova 32, 119991 Moscow, Russia; uzhny@mail.ru; 7 Martini-Klinik Prostate Cancer Center, University Hospital Hamburg-Eppendorf, Martinistrasse 52, 20246 Hamburg, Germany; graefen@uke.de; 8 Laboratory of Pharmacology, A.V. Zhirmunsky National Scientific Center of Marine Biology, Palchevskogo Str. 17, 690041 Vladivostok, Russia

**Keywords:** fascaplysin, synthesis, structure–activity relationships, prostate cancer, cytotoxicity, anticancer activity, selectivity

## Abstract

Marine alkaloid fascaplysin and its derivatives are known to exhibit promising anticancer properties in vitro and in vivo. However, toxicity of these molecules to non-cancer cells was identified as a main limitation for their clinical use. Here, for the very first time, we synthesized a library of fascaplysin derivatives covering all possible substituent introduction sites, i.e., cycles A, C and E of the 12*H*-pyrido[1-2-*a*:3,4-*b*’]diindole system. Their selectivity towards human prostate cancer versus non-cancer cells, as well as the effects on cellular metabolism, membrane integrity, cell cycle progression, apoptosis induction and their ability to intercalate into DNA were investigated. A pronounced selectivity for cancer cells was observed for the family of di- and trisubstituted halogen derivatives (modification of cycles A and E), while a modification of cycle C resulted in a stronger activity in therapy-resistant PC-3 cells. Among others, 3,10-dibromofascaplysin exhibited the highest selectivity, presumably due to the cytostatic effects executed via the targeting of cellular metabolism. Moreover, an introduction of radical substituents at C-9, C-10 or C-10 plus C-3 resulted in a notable reduction in DNA intercalating activity and improved selectivity. Taken together, our research contributes to understanding the structure–activity relationships of fascaplysin alkaloids and defines further directions of the structural optimization.

## 1. Introduction

**Figure 1:**
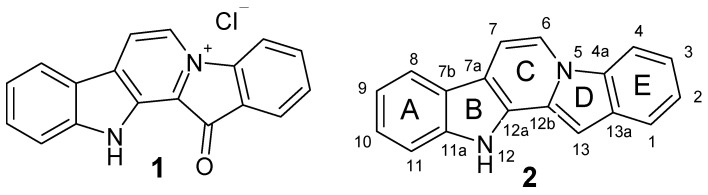
Structures of fascaplysin (**1**) and 12*H*-pyrido [1-2-*a*:3,4-*b*’]diindole ring system (**2**).

Compounds of natural origin possessing a wide range of pharmacological activity are widely used as lead molecules for the development of novel drugs, including anticancer therapeutics. Along with substances obtained from terrestrial plants and microorganisms, substances isolated from marine organisms have attracted particular interest [1,2,3]. One of these compounds is the red pigment fascaplysin (**1**, Figure 1). It was the very first representative of the group of marine alkaloids containing a 12*H*-pyrido [1-2-*a*:3,4-*b*’]diindole ring system (**2**) [4]. This group also includes homofascaplysins A–C, and their brominated analogues [5]. The therapeutic potential of fascaplysin (**1**) is based on a broad range of bioactivities, including anticancer, antibacterial, antifungal, antiviral and antimalarial properties [4,5,6].

However, the ability of fascaplysin to inhibit the proliferation of numerous cancer cell lines is of greatest interest. Fascaplysin effectively reduced the growth of small cell lung cancer (SCLC) spheroids derived from circulating tumor cells [7,8]. Fascaplysin also suppressed the growth of S180 cell-implanted tumors in vivo [9]. For decades, the major mechanisms of its anticancer action were considered as (i) selective inhibition of cyclin-dependent kinase 4 (CDK4), which regulates the G0–G1/S checkpoint of the cell cycle; (ii) intercalation with DNA; and (iii) consecutive induction of apoptosis [10,11,12]. Significant efforts have been made to create selective inhibitors of CDK 4 based on nonplanar analogs of fascaplysin [13,14,15,16,17,18]. As a result, the derivative named CA224 was synthesized. This compound specifically inhibits the CDK4−cyclin D1 complex, blocks cancer cell proliferation at the G0/G1 phase and inhibits tubulin polymerization in vitro. Additionally, it has good therapeutic index and showed antitumor activity in vivo in an HCT-116 tumor model [19]. Later, it was shown that fascaplysin has stronger anti-cancer effects than other CDK4 inhibitors, including PD0332991 and LY2835219, in a lung cancer model harboring wild-type or mutant retinoblastoma (RB) gene [20]. Finally, Kumar et al. have suggested that fascaplysin exerts a cumulative anticancer effect which includes a mitochondrial membrane potential loss, an attenuation of angiogenesis, as well as apoptosis and autophagy induction in HL-60 cells [21]. The latter two effects were executed via suppression of the PI3K-AKT-mTOR signaling cascade. Oh et al. performed a high-throughput kinase binding assay and identified that PI3K, AKT and mTOR might not be directly affected by fascaplysin, whereas multiple types of cancer-related kinases, such as VEGFR3, VEGFR2 and TRKA, were inhibited by fascaplysin at concentrations lower than 3 µM. Moreover, the administration of fascaplysin strongly reduced the accumulation of HIF-1α, which is a key transcriptional factor for regulating tumor angiogenesis in hypoxic tumor in vitro and in vivo. Fascaplysin suppresses the phosphorylation of mTOR, 4EBP1 and p70S6K1, all of which are pivotal signaling molecules for the cap-dependent translation machinery and attenuated survivin protein synthesis by dissociating eIF4E from survivin mRNA [20]. Autophagy induced by fascaplysin was reported to be a cytoprotective response and was executed via ROS and p8 [22]. Fascaplysin also induced a phosphorylation of protein kinase B (PKB), AKT and AMPK, and could sensitize cancer cells to other drugs targeting these kinases [23]. Preliminary in vivo assays using various models have shown that fascaplysin can inhibit tumor growth at a dose of 1–5 mg/kg [20,21]. Taken together, fascaplysin is a promising lead compound for the development of new anticancer drugs.

Notably, some derivatives of fascaplysin were reported to have stronger therapeutic potential compared to the parental alkaloid. The synthetic chloro derivative of fascaplysin at C-3 effectively inhibits the VEGF-mediated microvessel growth as well as the blood vessel formation in vivo. In addition, it inhibits tumor growth in mice. The compound was fivefold less toxic for non-malignant cells and no toxicity was observed in the mice receiving the drug [24]. In addition, natural 3- and 10-bromofascaplysins (**3**, **5**) showed anti-cancer activity at submicromolar concentrations. This effect was, at least in part, mediated via an induction of caspase-8, -9 and -3-dependent apoptosis [25]. Antitumor effects of 3-bromofascaplysin and 10-bromofascaplysin were examined in an in vitro glioma C6 cell model. The cytotoxic activity of **3** and **5** was higher than that of an original fascaplysin molecule; 3-bromofascaplysin exhibited the strongest cytotoxicity in glioma C6 cells [26]. Recently, the two-step approach toward the synthesis of fascaplysin was utilized by our group to generate 3-bromofascaplysin and 3,10-dibromofascaplysin (**8**), which were previously isolated from natural sources [27]. Remarkably, 3,10-dibromofascaplysin was able to suppress the cell metabolism at non-cytotoxic concentrations [28]. Further studies to determine the mechanism of the antitumor activity conducted in myeloid leukemia cells revealed that **8** activates the transcription factor E2F1 and decreases the expression of several genes responsible for cancer cell survival [29]. In addition, in human prostate cancer cells, we identified JNK1/2 to be one of the primary molecular targets of **8**. Additionally, **8** could synergize with PARP-inhibitor Olaparib, presumably due to the induction of ROS production and consequent oxidative DNA damage mediated by the drug [30]. Additionally, **8** increased the effects of well-established drugs such as cytarabine, cisplatin, carboplatin, as well as docetaxel and cabazitaxel [28,29]. Despite some promising results published in recent years, the cytotoxicity to normal (non-cancer) cells was assumed to be the main challenge for fascaplysin and related compounds on their way to clinically approved drugs. The ability of fascaplysin derivatives to intercalate into DNA was identified as the main reason for these undesired effects [15].

In the current research, we have synthesized a library of various fascaplysin derivatives (Table 1) and further explored their anticancer properties to identify the most promising molecules as well as to reveal structure–activity correlations. Since most of the naturally occurring fascaplysin derivatives studied are halogen derivatives, we decided to expand the series with synthetic mono- and disubstituted halogen derivatives of fascaplysin. In addition, fascaplysin derivatives at the central ring have not been studied yet. To assess the prospects of introducing a substituent in this cycle, radicals of various sizes were introduced at different positions. To obtain them, existing methods for the synthesis of fascaplysin were adapted, and novel approaches were developed. Our data indicate favorable directions of further synthetic modification and optimization of fascaplysin-like compounds. In our study, we have used several human prostate cancer cell lines harboring different levels of drug resistance as well as human non-cancer cell lines as a reference model for the drug evaluation selectivity. Prostate cancer is known to be one of the most common cancer entity in men [31]. At the early stages of the disease, tumor growth depends on androgens and therefore can be suppressed by the antiandrogen therapy. However, the tumors progress into an androgen-independent phenotype over time. Ultimately, they even develop resistance to second-line treatment medications, including untargeted chemotherapeutics such as platin-based agents and taxane derivatives [32,33].

## 2. Results and Discussion

### 2.1. Chemistry

**Scheme 1:**
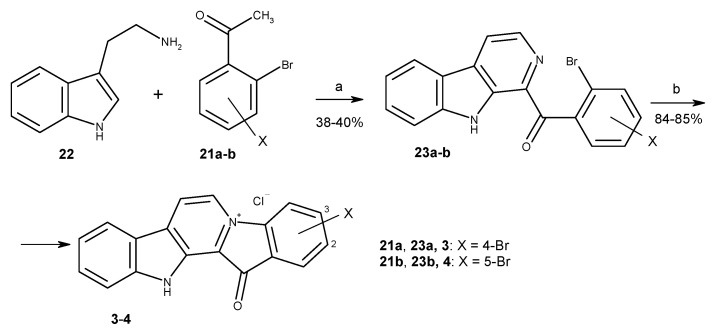
Synthesis of fascaplysin derivatives **3** and **4**. Reagents and conditions: (a) I_2_ (0.8 equiv.), DMSO, 110 °C, 1 h, then tryptamine (1.0 equiv.), DMSO, 110 °C, 4 h; (b) 220 °C, 15 min, then HCl (aq).

To date, more than ten methods to synthesize fascaplysin and its derivatives and analogs have been reported [34,35,36,37,38,39,40,41,42,43,44]. Most of them are based on the high-temperature quaternization of 1-benzoyl-β-carbolines (Stage **b**, Scheme 1), developed by Radchenko et al. [37] and with difference in the method of preparation of necessary β-carbolines. The two-step synthesis suggested by Zhu et al. is the most suitable for preparation of fascaplysin derivatives from substituted tryptamines and acetophenones [42]. This method was applied for the syntheses of fascaplysin and its derivatives **3** and **4** (Scheme 1). The cascade coupling protocol includes the sequential iodination of the corresponding acetophenone **21a-b**, the Kornblum oxidation of the intermediate in the presence of DMSO to phenylglyoxal, and its Pictet–Spengler condensation with tryptamine **22**, followed by the oxidation of the intermediate.

**Scheme 2:**
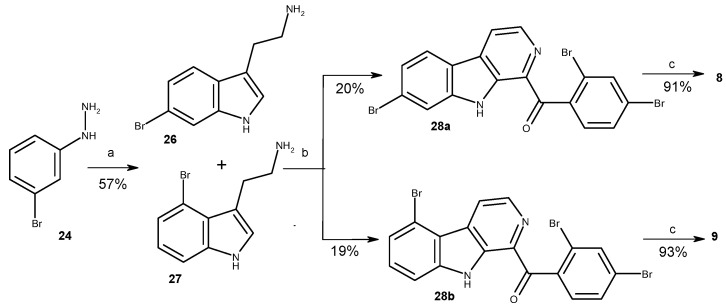
Synthesis of 3,10-dibromofascaplysin (**8**) and 3,8-dibromofascaplysin (**9**). Reagents and conditions: (a) 4-bromobutanal (**25**, 4.0 equiv.), EtOH, H_2_O, autoclave, 150 °C, 1 h; (b) 2,4-dibromoacetophenone (**21a**) (1 equiv.), I_2_ (0.8 equiv.), DMSO, 110 °C, 1 h, then tryptamines **26**, **27** (1.0 equiv.), DMSO, 110 °C, 4 h; (c) 220 °C, 15 min, then HCl (aq).

To apply this synthetic approach for the synthesis of 3,10-dibromofascaplysin (**8**), the reaction between 3-bromophenylhydrazine (**24**) and 4-bromobutanal (**25**) in an autoclave at 150 °C was used to prepare the mixture of 6-bromotryptamine (**26**) and 4-bromotryptamine (**27**). After chromatography purification, two isomeric 1-benzoyl-β-carbolines (**28a** and **28b**) were subsequently transformed to 3,10-dibromofascaplysin (**8**) and its isomer **9** with the procedure described above for fascaplysin (Scheme 2) [28].

**Scheme 3:**
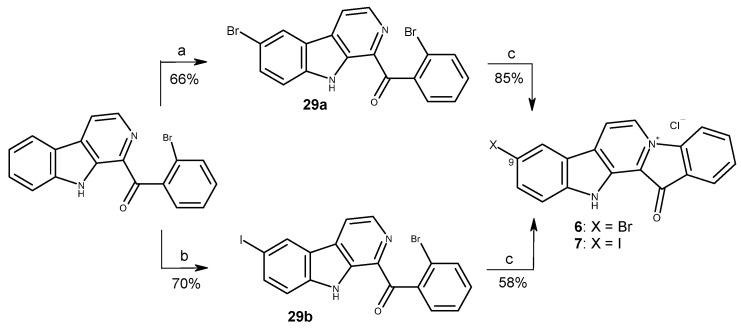
Synthesis of fascaplysin derivatives **6** and **7**. Reagents and conditions: (a) NBS (2.0 equiv.), CH_3_COOH, 90 °C, 1 h; (b) I_2_ (1.1 equiv.), TsOH (10 equiv.), DMSO, 110 °C, 1 h; (c) 220 °C, 0.5 h, then HCl (aq).

To simplify the preparation of variety of fascaplysin derivatives at cycle A, we initially used the interaction of fascaplysin at C-9 with molecular bromine and molecular chlorine in acetic acid, as described in the literature [45]. To expand the range of halogen derivatives of fascaplysin, the direct iodination of compound **1** was used by heating or microwave (MW) irradiation under various conditions (Table 2). However, the resulting mixtures of the reaction products were complex and hardly separatable. Instead, the direct bromination and iodination of 1-(2′-bromobenzoyl)-β-carboline were applied and ultimately resulted in 66–70% yields. Substituted β-carbolines **29a** and **29b** were further converted to fascaplysin derivatives **6** and **7** (Scheme 3). 10-Bromofascaplysin (**5**) was obtained using 2-bromophenylacetic acid and tryptamine **26** prepared as described previously [46].

**Scheme 4:**
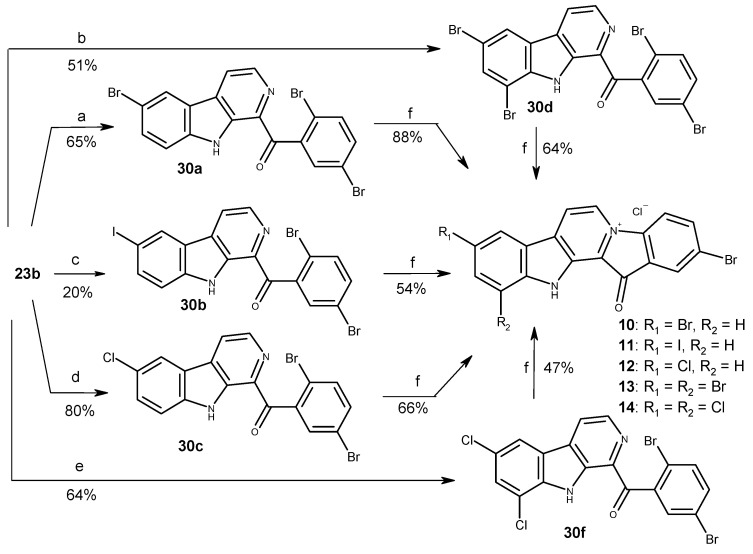
Synthesis of a series of disubstituted fascaplysin derivatives. Reagents and conditions: (a) NBS (2.0 equiv.), CH_3_COOH, 90 °C, 1 h; (b) NBS (4.0 equiv.), CH_3_COOH, 110 °C, 2 h; (c) I_2_ (1.1 equiv.), TsOH (10 equiv.), DMSO, 110 °C, 1 h; (d) Cl_2_ (excess), CH_3_COOH, r.t., 2 h; (e) Cl_2_ (excess), CH_3_COOH, 60 °C, 1 h; (f) 200 °C, 1–2 h, then HCl (aq).

It was also shown that chlorination and bromination conducted at higher temperatures lead to the production of compounds containing substituents at C-6 and C-8 of β-carboline skeleton. The combinational method developed by Zhu et al. with electrophilic substitution of intermediate β-carbolines allowed us to obtain a series of disubstituted derivatives of fascaplysin carrying the substitutes at rings A and E. Thus, chlorination, bromination and iodination of β-carboline **23b** and the subsequent quaternization of obtained products enabled us to prepare fascaplysin derivatives **10**–**14** (Scheme 4). In addition, for the evaluation of the effect of substitution of fascaplysin at C-3 and C-9, we carried out the bromination of β-carboline **23a** and the conversion of corresponding 1-benzoyl-β-carboline (**30 g**) into dibromo-substituted fascaplysin **15**.

**Scheme 5:**
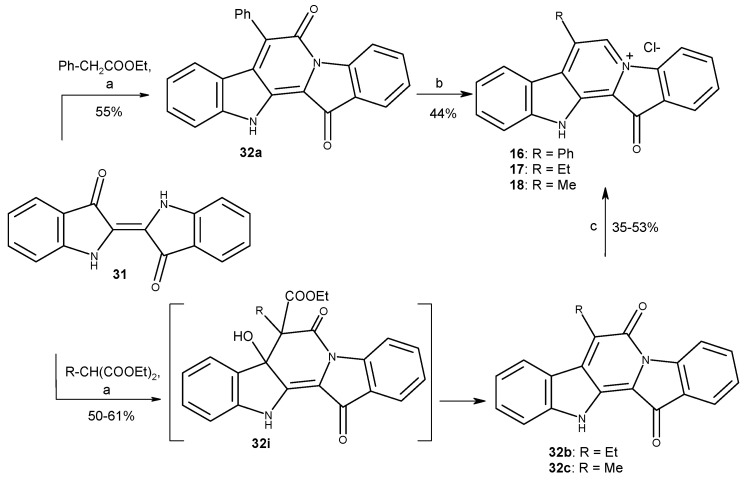
Syntheses of fascaplysin derivatives **16**–**18**. Reagents and conditions: (a) R-CH(COOEt)_2_ or PhCH_2_-COOEt (8 equiv.), NaH (4 equiv.), DMF, ∆, 0.5 h; (b) BH_3_·THF (24 equiv.), THF, Ar, ∆, 2 h, then H_2_O/H^+^, air, ∆, 2 h; (c) BH_3_·THF (24 equiv.), THF (autoclave), MW, 50 W, 30 min, then H_2_O/H^+^, air, ∆, 2 h.

To obtain fascaplysin derivatives at C-7, the condensation of the well-known dye indigo (**31**) with methylene active compounds was applied for the 12*H*-pyrido [1,2-*a*:3,4-*b*’]diindole ring system formation [37]. At the stage of condensation of indigo with ethyl methylmalonate and ethyl ethylmalonate, compounds **32b** and **c** were formed instead of expected intermediates **32i**. At the next step, the usage of the BH_3_–THF complex for the reduction of compounds **32b** and **c** under previously developed conditions of preparing of compound **16** (R = Ph) was not effective [43]. Target compounds **17** and **18** were obtained in an autoclave under microwave irradiation (Scheme 5).

**Scheme 6:**
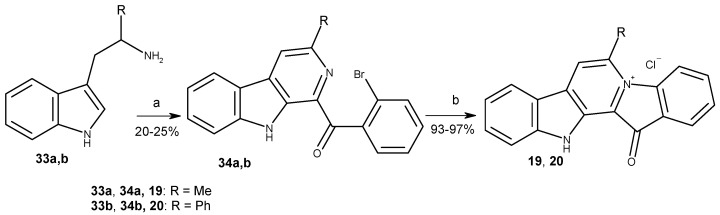
Syntheses of 6-methylfascaplysin (**19**) and 6-phenylfascaplysin (**20**). Reagents and conditions: (a) 2-bromoacetophenone (1.0 equiv.), I2 (0.8 equiv.), DMSO, 110 °C, 1 h, then tryptamines 33 (1.0 equiv.), DMSO, 110 °C, 4 h; (b) 220 °C, 15 min, then HCl (aq).

For comparison of the biological activities of fascaplysin derivatives at C-6 and C-7, 6-methylfascaplysin (**19**) and 6-phenylfascaplysin (**20**) were prepared with the method of Zhu using α-methyltryptamine (**33a**) and α-phenyltryptamine (**33b**). The syntheses have been described from isatin in the literature (Scheme 6) [47].

### 2.2. Biological Studies

#### 2.2.1. Activity and Selectivity of the Synthesized Compounds in Human Prostate Cancer Cells

In order to evaluate the anticancer potential of the synthesized compounds, we examined their cytotoxic effects using a panel of various human cell lines using a well-established MTT assay (Table S1). Thus, effects on human prostate cancer PC3, 22Rv1, DU145 and LNCaP cells, as well as human normal (non-cancer) PNT2, MRC-9 and HEK293 cell lines were examined. All the synthesized derivatives were active in this assay, having IC_50_ < 5 µM (and often <1 µM) in the vast majority of the cancer cell lines (Table S1). In general, the evaluated compounds have revealed certain selectivity towards hormone-independent 22Rv1 cells and lower activity in PC-3 cells, which are also known to be resistant to numerous hormonal treatment approaches and standard chemotherapies [48] (Table S1). Therefore, 22Rv1 cell lines have been chosen for the following examinations to determine antiproliferative and cytotoxic properties of the synthesized derivatives. As the MTT assay assesses metabolic activity of the cells [49], we have further applied a trypan blue staining assay, which allows us to distinguish between cells with intact (trypan blue-negative alive cells) and permeabilized cell membrane (trypan blue-positive dead cells) (Table S1). Additionally, a flow cytometry technique combined with propidium iodide staining was utilized to assess an effect on cell cycle progression as well as induction of apoptosis, which was measured as drug-induced DNA fragmentation (Table S1). Finally, the lipophilicity of the synthesized molecules was estimated in silico using the software ClogP (www.molinspiration.com, accessed on 31 January 2022) (Table S1).

**Figure 2:**
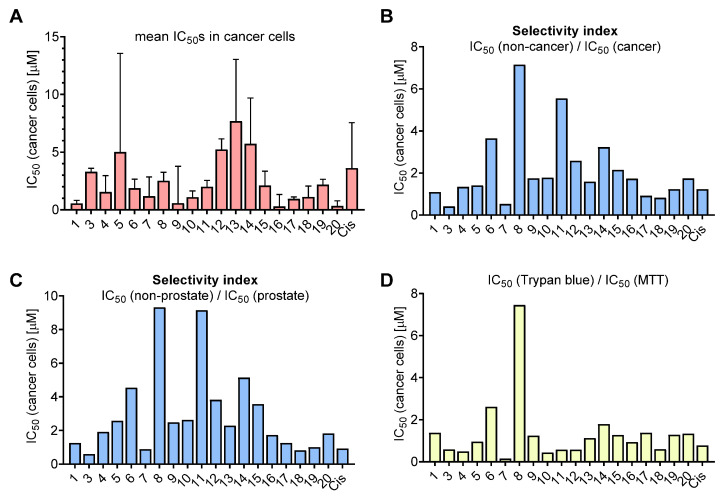
Activity and selectivity of the synthesized compounds in cancer versus non-cancer cell lines. (**A**) Mean IC_50_ evaluated using MTT assay in four prostate cancer cell lines (i.e., PC-3, 22Rv1, DU145 and LNCaP). Data are represented as mean ± SD. (**B**,**C**) Selectivity index (SI) calculated as: ratio of mean IC_50_ (non-cancer cell lines) versus mean IC_50_ (cancer cell lines) (**B**), or as mean IC_50_ (non-cancer non-prostate cell lines, i.e., MRC-9 and HEK-293) versus mean IC_50_ (prostate cells, i.e., prostate cancer cell lines plus PNT2 cells) (**C**). (**D**) Ratio of IC_50_ (22Rv1 cells, trypan blue assay) to IC_50_ (22Rv1 cells, MTT assay). The calculated values are presented in Supplementary Table S2. The values used for calculations (**A**–**D**) correspond to those shown in Table S1.

Next, SAR analysis was performed to identify the structural elements that are beneficial for anticancer activity of the fascaplysin-related molecules. Considering the complex and heterogeneous nature of tumors even within the same patient, we used calculated mean IC_50_ values of each drug in all prostate cancer cells for further analysis (Figure 2A). To identify the drug candidates with the highest therapeutic potential, the selectivity index (SI) was calculated as mean IC_50_ in non-cancer cells versus mean IC_50_ in cancer cells (Figure 2B), or as a mean IC_50_ in non-cancer (MRC-9 and HEK293) cells versus mean IC_50_ in prostate cells (cancer PC-3, 22Rv1, DU145, LNCaP and non-cancer PNT2 cells) (Figure 2C). Finally, to estimate the effects on cellular metabolism, we compared the activity evaluated by using trypan blue assay versus MTT assay (Figure 2D).

In general, an introduction of a single radical either to the cycle E (3-Br (**3**)) or (2-Br (**4**)), cycle A (10-Br (**5**), 9-Br (**6**) and 9-I (**7**)) or cycle C of the fascaplysin core (7-Et (**17**), 7-Me (**18**), 6-Me (**19**)) did not result in increased cytotoxic activity in cancer cells when compared to the activity of fascaplysin (Figure 2A). At the same time, a tendency towards a higher selectivity index (SI) was observed when the radical was introduced in cycle A (Figure 2B,C). Thus, the SI of bromo-derivatives **5** and **6** were twice as high compared to fascaplysin (**1**). Interestingly, moving the bromine atom from C-10 (**5**) to C-9 (**6**) resulted in a significant decrease in cytotoxic activity in non-malignant cells and therefore lead to a higher selectivity for tumor cells. On the other hand, the introduction of the iodine instead of bromine at C-9 dramatically increases the cytotoxicity of fascaplysin derivative (**7**) to non-cancer cells, which resulted in a SI < 1 (Figure 2B,C). Of note, comparison of cycle C derivatives **16**–**20** indicated an increase in cytotoxicity in the row Me- < Et- < Ph- (i.e., **18** < **17** < **16**). Moreover, phenyl-derivatives **16** and **20** had the highest cytotoxicity among all the tested molecules as well as some selectivity towards tumor cells (Figure 2B,C). Comparison of isomeric phenyl- (compounds **16** and **20** and methylfascaplysins (compounds **18** and **19**) did not reveal any significant difference in the activity between C-6 and C-7 derivatives. Of note, compounds possessing the modified cycle C were the only derivatives which were equally cytotoxic to therapy-resistant PC-3 cells, whereas other derivatives were less active in this cell line (Table S1).

The highest well-pronounced selectivity towards prostate cancer cells coupled with rather low absolute IC_50_ values was observed for the family of di- and trisubstituted halogen derivatives **8**–**15** (cycles A and E), making this group the most promising among the synthesized fascaplysin derivatives. Among all the synthesized compounds, 3,10-dibromofascaplysin (**8**) exhibited the highest selectivity to cancer cells, with SI = 7–9 (Figure 2B,C; Table S2). Within this group, moving the bromine atom at cycle A from C-8 (**9**) to C-9 (**15**) lead to a significant decrease in the cytotoxic effects on cancer cells, whereas the selectivity (SI) was not significantly affected.

Finally, based on a comparison of the cytotoxic activity of isomeric bromofascaplysins at C-3 (**3**) and C-2 (**4**), as well as C-10 (**5**) and C-9 (**6**), we speculated that 2,9-disubstituted fascaplysins should exhibit the most promising anticancer properties. In order to verify this, a series of derivatives **10**–**12**, possessing various halogen atoms in C-9, was synthesized. Unfortunately, 2,9-dibromofascaplysin (**10**) had neither stronger cytotoxicity nor higher selectivity. Moreover, neither substitution of bromine atom at C-9 with iodine (**11**) or chlorine (**12**), nor introduction of additional bromine (**13**) or chlorine (**14**) at cycle A resulted in better anticancer properties.

Interestingly, for two derivatives, namely compounds **6** and **8**, the IC_50_ determined by the trypan blue exclusion assay was 2.5- and 7.5-fold higher than by the MTT assay (Figure 2D). Therefore, derivatives **6** and **8** have been assumed to have a primary and probably specific effect on cellular metabolism, an inhibition which is detected in the MTT test. In contrast, the cellular membrane integrity assessed by trypan blue assay was not yet affected.

Previously, fascaplysin has been shown to induce G1 phase arrest of the cell cycle, presumably due to inhibition of CDK4 [25,26]. In line with previous reports, in our experiments, fascaplysin and most of its derivatives also induced G1-phase arrest of cell cycle progression of prostate cancer 22Rv1 cells. Additionally, all the compounds induced a certain degree of DNA fragmentation at their IC_50_. However, no correlation of either effect with the derivatives structure could be found (Table S1). Similar to this, no correlations of biological activity and lipophilicity were observed for the synthesized compounds (Table S1).

#### 2.2.2. Interaction of Fascaplysin and its Derivatives with DNA

**Figure 3:**
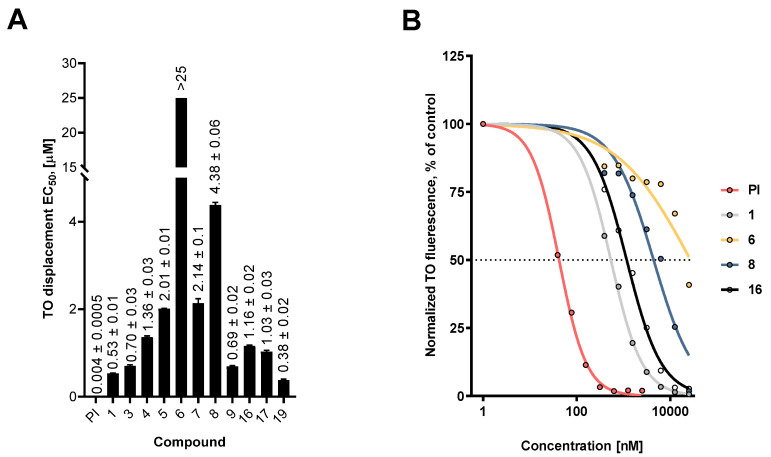
DNA intercalating activity of the synthesized fascaplysin derivatives. (**A**) Calculated half-maximal effective concentrations (EC_50_), determined using the fluorescent intercalator displacement assay. (**B**) Representative fluorescence curves for selected compounds. Propidium iodide was used as a positive control. Data are represented as mean ± SD.

Since DNA intercalation is considered an important component of the biological mechanism of action of fascaplysin, we studied this ability in selected synthesized derivatives. Drug–DNA complex formation was evaluated by a fluorescent intercalator displacement assay, based on detachment from DNA of the intercalative agent thiazole orange (TO) by tested compounds. The half-maximal concentrations for effective displacement of TO from the complex with DNA were calculated and are represented in Figure 3. In this experiment, a well-established DNA-binding compound, propidium iodide (PI), was used as a positive control. We found that most of the tested compounds effectively displaced TO from the fluorescent complex with DNA, indicating possible DNA intercalation.

**Figure 4:**
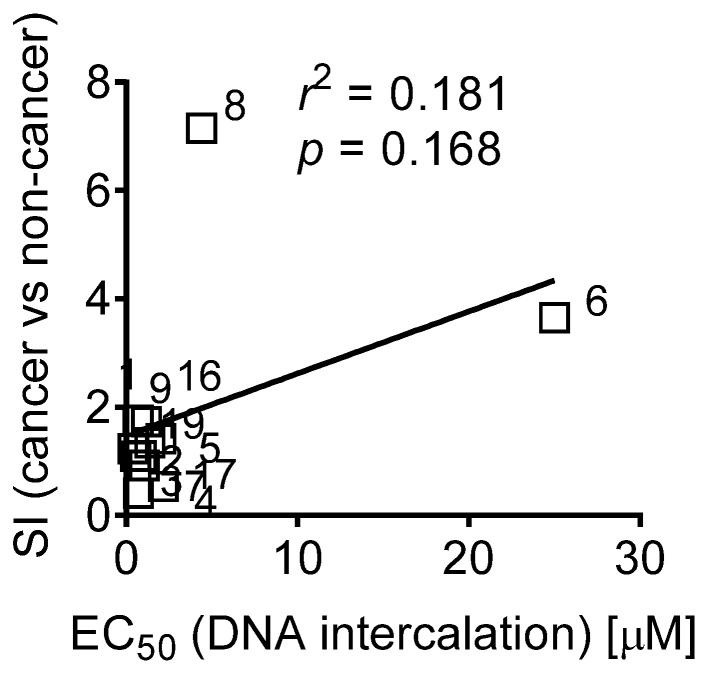
Correlation of the DNA intercalating half-maximal effective doses (EC_50_) of the synthesized compounds and their selectivity index (SI). SI was calculated as ratio of mean IC_50_ (non-cancer cell lines) versus mean IC_50_ (cancer cell lines) (Table S2; Figure 2B). The Pearson correlation coefficient (*r*) and its *p*-value were calculated using GraphPad Prism software v.9.1.1.

Comparison of these data with the data from Tables S1 and S2 revealed a correlation of DNA binding activity with cytotoxic activity as well as anticorrelation with selectivity in cancer cells. Thus, compounds having high SI had greater EC_50_ value (Figure 4). Moreover, compounds **6** and **8**, which presumably were capable of cellular metabolism targeting and revealing the highest IC_50_ (trypan blue)/IC_50_ (MTT) index, also had high EC_50s_, suggesting a low DNA intercalating activity.

Interestingly, the introduction of a bulky phenyl radical to the central cycle of fascaplysin did not affect its ability to interact with DNA. This may be due to the fact that (i) the central part of compound **1** molecule is not involved in the formation of a complex with DNA, or (ii) during the formation of a complex of DNA and **16**, the phenyl radical is in the same plane with the main skeleton of fascaplysin. Hence, the introduction of bulky and nonplanar radicals (e.g., *tert*-butyl) to various positions of the fascaplysin core might be beneficial for anticancer activity of this molecule and is of particular interest.

Taken together, it seems that the DNA intercalating activity indeed stipulates the non-selective cytotoxicity of the fascaplysin-like molecules. However, reduction of this activity, at least within the families of the derivatives bearing substitutes in cycles A, E or both, resulted in better selectivity towards cancer cells. The mechanisms of this phenomenon may be based on the antimetabolic activity and/or antiproliferative effects of the synthesized derivatives and are to be investigated in the future.

## 3. Materials and Methods

### 3.1. Chemistry

All starting materials are commercially available. Commercial reagents were used without any purification. The Discover^®^ microwave synthesis system was used for MW irradiation (CEM Corporation, Matthews, USA). The products were isolated by MPLC: Buchi B-688 pump, glass column C-690 (15 × 460 mm) or PP-cartridge (150 × 12 mm) with Silica gel (particle size 0.015–0.040 mm), UV-detector Knauer K-2001 (BÜCHI Labortechnik AG, Flawil, Switzerland). The analytical examples were purified by Shimadzu HPLC system (model: LC-20AP Shimadzu Europa GmbH, Duisburg, Germany) equipped with a UV detector (model: SPD-20A) using Supelco C18 (5 µm, 20 × 250 mm) column using MeOH:water (20:80, 50:50, 70:30) mobile phase by isocratic elution at flow rate of 15 mL/min. NMR spectra were recorded with an NMR instrument (Bruker BioSpin International, Zug, Switzerland) operating at 400 MHz (^1^H) and 100 MHz (^13^C). Proton spectra were referenced to TMS as internal standard, in some cases, to the residual signal of used solvents. Carbon chemical shifts were determined relative to the ^13^C signal of TMS or used solvents. Chemical shifts are given on the δ scale (ppm). Coupling constants (J) are given in Hz. Multiplicities are indicated as follows: s (singlet), d (doublet), t (triplet), q (quartet), m (multiplet) or br (broadened). The original spectra of the relative compounds can be found in Supplementary Materials. High-resolution mass spectra (HRMS) were obtained with a time-of-flight (TOF) mass spectrometer (model Agilent TOF 6210) equipped with an electrospray source at atmospheric pressure ionization (ESI).

#### 3.1.1. Preparation of Mixture of Tryptamines **26** and **27**

A mixture of 4-bromobutanal (1.33 g, 8.8 mmol), 3-bromophenylhydrazine hydrochloride (0.50 g, 2.2 mmol), EtOH (3 mL) and H_2_O (1 mL) was placed into an autoclave and heated at 150 °C for 1 h. After cooling, the mixture was poured into H_2_O (200 mL) and extracted with EtOAc (3 × 50 mL). Then, the aqueous solution was treated with NaOH to pH 12 and extracted with CH_2_Cl_2_ (3 × 50 mL). The combined organic layer was washed with brine (2 × 100 mL), dried over Na_2_SO_4_ and evaporated. After flash column chromatography (EtOAc, then EtOH/NH_3_), compounds **26** and **27** were isolated as a mixture in a ratio of 1:1 (brown oil, 300 mg, 57%).

#### 3.1.2. Preparation of Substituted 1-Benzoyl-β-Carbolines **23a-b**, **28a-b**, **35a-b**

Corresponding acetophenone (0.458 mmol) and iodine (92 mg, 0.366 mmol) were added to 2 mL of DMSO, and the resulting solution was heated at 90 °C for 1 h. After that, tryptamine, its derivative or their mixture (0.458 mmol) was added to the solution and this solution was stirred at the same temperature for 3–4 h until completion of the reaction (monitored by TLC). Then, the reaction mixture was cooled to room temperature followed by the addition of water (50 mL) and extraction with EtOAc (2 × 25 mL). The extract was washed with 10% Na_2_S_2_O_3_, dried over Na_2_SO_4_, filtered and evaporated under reduced pressure. The residue was purified by MPLC using benzene or benzene/hexanes as eluent to give the desired product.

For 1-(2′,4′-dibromobenzoyl)-β-carboline (**23a**): yellow solid, 38%. ^1^H NMR (400 MHz, DMSO-d6): *δ* 12.23 (br. s, 1H, NH), 8.48 (d, *J* = 4.9, 1H, H-3), 8.44 (d, *J* = 4.9, 1H, H-4), 8.34 (d, *J* = 7.9, 1H, H-5), 8.02 (d, *J* = 1.9, 1H, H-3′), 7.85 (d, *J* = 8.0, 1H, H-8), 7.76 (dd, *J* = 8.3, 1.9, 1H, H-5′), 7.64 (ddd, *J* = 7.2, 7.2, 1.0, 1H, H-7), 7.57 (d, *J* = 8.3, 1H, H-6′), 7.35 (ddd, *J* = 7.2, 7.2, 1.0, 1H, H-6). ^13^C NMR (100 MHz, DMSO-d6): *δ* 195.9, 142.0, 140.5, 137.9, 135.3, 134.8, 134.3, 131.4, 131.0, 130.3, 129.2, 128.3, 123.2, 121.9, 120.5, 120.0, 119.8, 113.1. HRMS-ESI, *m*/*z*: [M + H]^+^ calculated for C_18_H_11_^79^Br_2_N_2_O^+^ 428.9238, found 428.9264.

For 1-(2′,5′-dibromobenzoyl)-β-carboline (**23b**): yellow solid, 40%. ^1^H NMR (400 MHz, CDCl_3_): *δ* 10.52 (br. s., 1H), 8.61 (d, *J* = 4.9 Hz, 1H), 8.35 (d, *J* = 1.9 Hz, 1H), 8.16 (d, *J* = 5.0, 1H), 7.76 (dd, *J* = 8.7, 1.9 Hz, 1H), 7.73 (dd, *J* = 8.1, 1.1 Hz, 1H), 7.59 (dd, *J* = 7.4, 1.7 Hz, 1H), 7.56 (d, *J* = 8.6 Hz, 1H), 7.51 (ddd, *J* = 7.5, 1.1 Hz, 1H), 7.43 (ddd, *J* = 7.7, 1.8 Hz, 1H). ^13^C NMR (100 MHz, CDCl_3_): *δ* 198.4, 140.3, 140.0, 139.4, 137.2, 135.8, 133.4, 132.5, 131.6, 131.0, 130.1, 127.1, 124.9, 122.7, 120.3, 119.5, 114.0, 113.8. HRMS-ESI, *m*/*z*: [M + H]^+^ calculated for C_18_H_11_^79^Br_2_N_2_O^+^ 428.9235, found 428.9241.

For 1-(2′,4′-dibromobenzoyl)-7-bromo-β-carboline (**28a**): yellow solid, 20%. ^1^H NMR (400 MHz, CDCl_3_): *δ* 10.45 (br. s, 1H), 8.57 (d, *J* = 4.9 Hz, 1H), 8.15 (d, *J* = 4.9 Hz, 1H), 8.05 (d, *J* = 8.3 Hz, 1H), 7.88 (d, *J* = 1.7 Hz, 1H), 7.79 (d, *J* = 1.1 Hz, 1H), 7.62 (dd, *J* = 8.3, 1.7 Hz, 1H), 7.50 (dd, *J* = 8.3, 1.5 Hz, 1H), 7.46 (d, *J* = 8.2 Hz, 1H). ^13^C NMR (100 MHz, CDCl_3_): *δ* 196.7, 141.4, 138.9, 138.4, 136.5, 135.2, 134.7, 130.9, 130.6, 129.8, 124.4, 124.2, 122.9, 122.6, 120.5, 119.2, 118.9, 114.8. HRMS-ESI, *m*/*z*: [M + H]^+^ calculated for C_18_H_10_^79^Br_3_N_2_O^+^: 506.8340, found 506.8345.

For 1-(2′,4′-dibromobenzoyl)-5-bromo-β-carboline (**28b**): yellow solid, 19%. ^1^H NMR (400 MHz, CDCl_3_): *δ* 10.55 (br. s, 1H), 8.81 (d, *J* = 5.1 Hz, 1H), 8.62 (d, *J* = 5.1 Hz, 1H), 7.88 (d, *J* = 1.8 Hz, 1H), 7.63 (dd, *J* = 3.2, 1.4 Hz, 1H), 7.61 (dd, *J* = 2.9, 1.4 Hz, 1H), 7.54–7.58 (m, 1H), 7.51 (d, *J* = 7.80 Hz, 1H), 7.46 (d, *J* = 8.2 Hz, 1H). ^13^C NMR (100 MHz, CDCl_3_): *δ* 197.5, 142.4, 139.4, 139.1, 137.0, 135.9, 135.1, 131.6, 131.3, 130.4, 130.2, 125.4, 125.1, 121.5, 121.2, 120.3, 118.5, 111.3. HRMS-ESI, *m*/*z*: [M + H]^+^ calculated for C_18_H_10_^79^Br_3_N_2_O^+^ 506.8340, found 506.8347.

For 1-(2′-bromobenzoyl)-3-methyl-β-carboline (**35a**): yellow solid, 20%. ^1^H NMR (400 MHz, CDCl_3_): *δ* 10.37 (br. s., 1H), 8.52 (s, 1H), 8.23 (d, *J* = 5.1 Hz, 1H), 8.00 (d, *J* = 5.0 Hz, 1H), 7.73 (dd, *J*= 7.6, 1.7 Hz, 1H), 7.68 (d, *J* = 7.7 Hz, 1H), 7.61 (d, *J* = 7.8 Hz, 1H), 7.57 (dd, *J* = 7.4, 1.7 Hz, 1H), 7.48 (dd, *J* = 7.2 Hz, 1H), 7.40 (ddd, *J* = 7.7, 1.8 Hz, 1H), 2.62 (s, 3H). ^13^C NMR (100 MHz, CDCl_3_): *δ* 193.8, 149.7, 149.2, 145.0, 142.5, 138.5, 136.0, 132.7, 132.1, 127.2, 126.9, 125.5, 124.5, 124.5, 123.7, 121.3, 120.1, 113.8, 25.9. HRMS-ESI, *m*/*z*: [M + H]^+^ calculated for C_19_H_14_^79^BrN_2_O^+^ 365.0289, found 365.0370.

For 1-(2′-bromobenzoyl)-3-phenyl-β-carboline (**35b**): yellow solid, 25%. ^1^H NMR (400 MHz, CDCl_3_): *δ* 10.38 (br. s., 1H), 8.65 (s, 1H), 8.25 (d, *J* = 7.8 Hz, 1H), 8.02 (d, *J* = 1.4 Hz, 1H), 8.01 (s, 1H), 7.74 (dd, *J* = 8.0, 1.0 Hz, 1H), 7.68 (dd, *J* = 7.5 Hz, 1.7 Hz, 1H), 7.64–7.66 (m, 2H), 7.36–7.52 (m, 6H). ^13^C NMR (100 MHz, CDCl_3_): *δ* 199.6, 148.2, 143.2, 141.8, 141.0, 137.7, 135.7, 134.7, 134.5, 132.7, 132.1, 131.0, 130.3, 129.7, 128.2, 128.1, 123.4, 122.7, 122.6, 122.1, 117.5, 117.2, 113.7. HRMS-ESI, *m*/*z*: [M + H]^+^ calculated for C_24_H_16_^79^BrN_2_O^+^ 427.0446, found 427.0465.

#### 3.1.3. Preparation of Substituted 1-Benzoyl-β-Carbolines **29a**, **30a**, **30g**

Substituted β-carboline (0.1165 mmol) was mixed with NBS (41.5 mg, 0.2330 mmol), and acetic acid (3 mL) was added to the mixture. The resulting mixture was heated at 90 °C for 1 h. Then, the reaction mixture was cooled to room temperature followed by the addition of saturated aqueous solution of Na_2_CO_3_ (50 mL) and extraction with EtOAc (2 × 25 mL). The extract was washed with H_2_O, dried over Na_2_SO_4_, filtered and evaporated under reduced pressure. The residue was purified by MPLC using benzene to give the target compound.

For 1-(2′-bromobenzoyl)-6-bromo-β-carboline (**29a**): yellow solid, 66%. ^1^H NMR (400 MHz, CDCl_3_): *δ* 10.47 (br. s, 1H), 8.58 (d, *J* = 4.9 Hz, 1H), 8.32 (d, *J* = 1.9 Hz, 1H), 8.13 (dd, *J* = 4.9, 0.5 Hz, 1H), 7.73 (dd, *J* = 8.7, 1.9 Hz, 1H), 7.70 (dd, *J* = 8.0, 1.0 Hz, 1H), 7.56 (dd, *J* = 7.5, 1.7 Hz, 1H), 7.53 (d, *J* = 8.6 Hz, 1H), 7.49 (dd, *J* = 7.5, 1.1 Hz, 1H), 7.47 (dd, *J* = 7.5, 1.1 Hz, 1H), 7.40 (ddd, *J* = 7.7, 1.7 Hz, 1H). ^13^C NMR (100 MHz, CDCl_3_): *δ* 198.1, 140.0, 139.7, 139.2, 137.0, 135.6, 133.2, 132.3, 131.4, 130.7, 129.8, 126.9, 124.7, 122.5, 120.1, 119.3, 113.8, 113.6. HRMS-ESI, *m*/*z*: [M + H]^+^ calculated for C_18_H_11_^79^Br_2_N_2_O^+^ 428.9238, found 428.9260.

For 1-(2′,5′-dibromobenzoyl)-6-bromo-β-carboline (**30a**): yellow solid, 65%. ^1^H NMR (400 MHz, CDCl_3_): *δ* 10.42 (br. s., 1H), 8.57 (d, *J* = 5.0 Hz, 1H), 8.31 (d, *J* = 1.7 Hz, 1H), 8.13 (d, *J* = 5.0 Hz, 1H), 7.87 (d, *J* = 1.8 Hz, 1H), 7.74 (dd, *J* = 8.2, 1.8 Hz, 1H), 7.61 (dd, *J* = 8.1, 1.8 Hz, 1H), 7.53 (d, *J* = 8.6 Hz, 1H), 7.45 (d, *J* = 8.2 Hz, 1H). ^13^C NMR (101 MHz, CDCl_3_): *δ* 197.1, 166.8, 139.7, 139.1, 138.8, 135.7, 135.2, 132.4, 131.0, 130.8, 130.2, 124.9, 124.7, 122.4, 121.0, 119.5, 113.9, 113.6. HRMS-ESI, *m*/*z*: [M + H]^+^ calculated for C_18_H_10_^79^Br_3_N_2_O^+^ 506.8340, found 506.8346.

For 1-(2′,4′-dibromobenzoyl)-6-bromo-β-carboline (**30g**): yellow solid, 67%. ^1^H NMR (400 MHz, CDCl_3_): *δ* 10.42 (br. s., 1H), 8.57 (d, *J* = 4.9 Hz, 1H), 8.31 (s, 1H), 8.13 (d, *J* = 5.0 Hz, 1H), 7.87 (s, 1H), 7.73 (d, *J* = 8.7 Hz, 1H), 7.61 (d, *J* = 8.1 Hz, 1H), 7.52 (d, *J* = 8.7 Hz, 1H), 7.44 (d, *J* = 8.2 Hz, 1H). ^13^C NMR (101 MHz, CDCl_3_): *δ* 197.1, 139.7, 139.2, 138.8, 137.0, 135.7, 135.2, 132.4, 131.1, 130.8, 130.2, 124.9, 124.8, 122.5, 121.0, 119.5, 113.9, 113.6. HRMS-ESI, *m*/*z*: [M + H]^+^ calculated for C_18_H_10_^79^Br_3_N_2_O^+^ 506.8340, found 506.8351.

#### 3.1.4. Synthesis of Compound **30d**

β-Carboline **23c** (50 mg, 0.1165 mmol) was mixed with NBS (83 mg, 0.466 mmol), and acetic acid (3 mL) was added to the mixture. The resulting mixture was heated at 110 °C for 2 h. Then, the reaction mixture was cooled to room temperature followed by the addition of saturated aqueous solution of Na_2_CO_3_ (50 mL) and extraction with EtOAc (2 × 25 mL). The extract was washed with H_2_O, dried over Na_2_SO_4_, filtered and evaporated under reduced pressure. The residue was purified by MPLC using benzene to give compound **30d** (35 mg, 51%) as a yellow solid. ^1^H NMR (400 MHz, CDCl_3_): *δ* 10.43 (br. s., 1H), 8.64 (d, *J* = 5.0 Hz, 1 H), 8.29 (d, *J* = 1.2 Hz, 1H), 8.16 (d, *J* = 5.0 Hz, 1H), 7.96 (d, *J* = 1.7 Hz, 1H), 7.92 (d, *J* = 1.9 Hz, 1H), 7.65 (dd, *J* = 8.2, 1.8 Hz, 1H), 7.47 (d, *J* = 8.2 Hz, 1H). ^13^C NMR (101 MHz, CDCl_3_): *δ* 196.8, 139.7, 138.8, 138.5, 136.4, 135.8, 135.7, 134.0, 131.1, 131.1, 130.2, 125.0, 123.8, 123.1, 121.0, 119.9, 113.8, 106.1. HRMS-ESI, *m*/*z*: [M + H]^+^ calculated for C_18_H_9_^79^Br_4_N_2_O^+^: 585.7523, found 585.7541.

#### 3.1.5. Preparation of Compounds **29b**, **30b**

Corresponding β-carboline (0.04 mmol), TsOH (50 mg, 0.5 mmol) and iodine (112 mg, 0.44 mmol) were added to 2 mL of DMSO, and the resulting solution was heated at 110 °C for 1 h. Then, the reaction mixture was cooled to room temperature followed by the addition of water (50 mL) and extraction with EtOAc (2 × 25 mL). The extract was washed with 10% Na_2_S_2_O_3_, dried over Na_2_SO_4_, filtered and evaporated under reduced pressure. The residue was purified by MPLC using benzene, triturated with Et_2_O and dried to give the target product.

For 1-(2′-bromobenzoyl)-6-iodo-β-carboline (**29b**): yellow solid, 70%. ^1^H NMR (C_6_D_6_): *δ* 8.28 (d, 1H), 8.12 (d, 1H), 7.51 (dd, 1H), 7.40 (dd, 1H), 7.37 (d, 1H) 7.24 (d, 1H), 6.96 (dd, 1H), 6.76 (ddd, 1H), 6.33 (d, 1H). ^13^C NMR (C_6_D_6_): *δ* 197.7, 170.3, 140.7, 139.6, 138.5, 136.8, 135.8, 135.5, 132.4, 130.3, 130.1, 129.3, 126.1, 122.7, 119.9, 119.5, 118.5, 113.6. HRMS-ESI, *m*/*z*: [M + H]^+^ calculated for C_18_H_12_^79^BrIN_2_O^+^ 476,9099, found 476,9123.

For 1-(2′.5′-dibromobenzoyl)-6-iodo-β-carboline (**30b**): yellow solid, 20%. ^1^H NMR (C_6_D_6_): *δ* 9.97 (br.s., 1H), 8.31 (d, *J* = 4.9 Hz, 1H), 8.19 (d, *J* = 1.1 Hz, 1H), 7.61 (d, *J* = 3.0 Hz, 1H), 7.59 (dd, *J* = 8.6, 1.7 Hz, 1H), 7.30 (d, *J* = 4.9 Hz, 1H), 7.00 (d, *J* = 8.6 Hz, 1H), 6.91 (dd, *J* = 8.6, 2.4 Hz, 1H), 6.41 (d, *J* = 8.6 Hz, 1H). ^13^C NMR (C_6_D_6_): *δ* 195.8, 142.2, 139.5, 138.6, 136.9, 135.9, 134.8, 133.7, 133.2, 132.0, 130.1, 129.7, 122.6, 120.3, 119.5, 118.7, 118.4, 113.5. HRMS-ESI, *m*/*z*: [M + H]^+^ calculated for C_18_H_9_^79^Br_2_IN_2_O^+^ 554.8205, found 554.8237.

#### 3.1.6. Synthesis of Compound **30c**

β-Carboline **23b** (50 mg, 0.1165 mmol) was added to saturated solution of chlorine in acetic acid (4 mL). The resulting mixture was stirred at r.t. for 2 h. Then, the reaction mixture was cooled to room temperature followed by the addition of saturated aqueous solution of Na_2_CO_3_ (50 mL) and extraction with EtOAc (2 × 25 mL). The extract was washed with H_2_O, dried over Na_2_SO_4_, filtered and evaporated under reduced pressure. The residue was purified by MPLC using benzene to give compound **30c** (43 mg, 80%) as a yellow solid. ^1^H NMR (400 MHz, CDCl_3_): *δ* 10.42 (br. s., 1H), 8.56 (d, *J* = 5.0 Hz, 1H), 8.16 (d, *J* = 1.7 Hz, 1H), 8.14 (d, *J* = 4.9 Hz, 1H), 7.87 (d, *J* = 1.8 Hz, 1H), 7.56–7.62 (m, 3H), 7.45 (d, *J* = 8.2 Hz, 1H). ^13^C NMR (101 MHz, CDCl_3_): *δ* 197.1, 139.4, 139.1, 138.8, 137.2, 135.7, 135.2, 131.0, 130.9, 130.2, 129.8, 126.7, 124.8, 121.9, 121.7, 121.0, 119.5, 113.2. HRMS-ESI, *m*/*z*: [M + H]^+^ calculated for C_18_H_10_^79^Br_2_^35^ClN_2_O^+^ 462.8848, found 462.8876.

#### 3.1.7. Preparation of Compound **30f**

β-Carboline **23b** (50 mg, 0.1165 mmol) was added to saturated solution of chlorine in acetic acid (4 mL). The resulting mixture was heated at 60 °C for 1 h. Then, the reaction mixture was cooled to room temperature followed by the addition of saturated aqueous solution of Na_2_CO_3_ (50 mL) and extraction with EtOAc (2 × 25 mL). The extract was washed with H_2_O, dried over Na_2_SO_4_, filtered and evaporated under reduced pressure. The residue was purified by MPLC using benzene to give compound **30f** (37 mg, 64%) as a yellow solid. ^1^H NMR (400 MHz, CDCl_3_): *δ* 10.47 (br. s., 1H), 8.64 (d, *J* = 5.0 Hz, 1H), 8.17 (dd, *J* = 5.0, 1.0 Hz, 1H), 8.10 (d, *J* = 1.8 Hz, 1H), 7.92 (d, *J* = 1.8 Hz, 1H), 7.69 (d, *J* = 1.8 Hz, 1H), 7.66 (dd, *J* = 8.2, 1.8 Hz, 1H), 7.48 (d, *J* = 8.2 Hz, 1H). ^13^C NMR (101 MHz, CDCl_3_): *δ* 196.8, 139.6, 138.5, 137.0, 136.6, 135.8, 135.7, 131.1, 130.2, 128.8, 126.8, 125.0, 122.8, 121.0, 120.2, 119.8, 118.2, 115.9. HRMS-ESI, *m*/*z*: [M + H]^+^ calculated for C_18_H_9_^79^Br_2_^35^Cl_2_N_2_O^+^ 496.8458, found 496.8490.

#### 3.1.8. Quaternization of Substituted 1-Benzoyl-β-carbolines

Substituted 1-benzoyl-β-carboline (0.326 mmol) was heated in sealed tube at 200–220 °C for 0.5–2 h. After cooling, the reaction mixture was washed with EtOAc (3 × 3 mL) and H_2_O (3 × 10 mL). The combined aqueous layer was acidified with hydrochloric acid and evaporated under reduced pressure to give target product as a red powder.

For 3-bromofascaplysin (**3**): prepared at 220 °C, 0.5 h, red solid, 84%. ^1^H NMR (400 MHz, MeOH-d4): *δ* 9.35 (d, *J* = 6.2 Hz, 1H, H-6), 8.95 (d, *J* = 6.2 Hz, 1H, H-7), 8.68 (s, 1H, H-4), 8.48 (d, *J* = 8.1 Hz, 1H, H-8), 7.93 (s, 2H, H-1, H-2), 7.88 (t, *J* = 7.6 Hz, 1H, H-10), 7.79 (d, *J* = 8.1 Hz, 1H, H-11), 7.52 (t, *J* = 7.6 Hz, 1H, H-9). ^13^C-NMR (100 MHz, MeOH-d4): *δ* 182.0, 149.4, 148.9, 143.1, 136.0, 135.6, 132.3, 132.2, 127.7, 127.6, 125.1, 124.5, 124.5, 123.8, 121.1, 120.9, 120.3, 114.5. HRMS-ESI, *m*/*z*: [M]^+^ calculated for C_18_H_10_^79^BrN_2_O^+^ 348.9974, found 348.9980.

For 12,13-dihydro-2-bromo-13-oxopyrido[1,2-*a*:3,4-*b*’]diindol-5-ium chloride (**4**): prepared at 240 °C, 1 h, red solid, 85%. ^1^H NMR (400 MHz, MeOH-d4): *δ* 9.38 (d, *J* = 6.1 Hz, 1H), 8.97 (d, *J* = 6.0 Hz, 1H), 8.48 (d, *J* = 8.0 Hz, 1H), 8.29 (d, *J* = 8.5 Hz, 1H), 8.19 (d, *J* = 1.8 Hz, 1H), 8.14 (dd, *J* = 8.4, 1.7 Hz, 1H), 7.89 (t, *J* = 7.5 Hz, 1H), 7.79 (d, *J* = 8.3 Hz, 1H), 7.53 (t, *J* = 7.5 Hz, 1H). ^13^C-NMR (100 MHz, MeOH-d4): *δ* 182.1, 149.1, 147.6, 143.2, 140.6, 136.2, 133.2, 129.7, 128.0, 127.5, 126.1, 125.5, 124.8, 123.5, 121.4, 118.4, 115.5, 114.8. HRMS-ESI, *m*/*z*: [M]^+^ calculated for C_18_H_10_^79^BrN_2_O^+^ 348.9974, found 348.9982.

For 12,13-dihydro-9-bromo-13-oxopyrido[1,2-*a*:3,4-*b*’]diindol-5-ium chloride (**6**): prepared at 240 °C, 1 h, red solid, 85%. ^1^H NMR (400 MHz, MeOH-d4): *δ* 9.41 (d, *J* = 6.2 Hz, 1H), 8.98 (d, *J* = 6.2 Hz, 1H), 8.71 (d, *J* = 1.8 Hz, 1H), 8.34 (d, *J* = 8.1 Hz, 1H), 8.06 (dd, *J* = 7.5, 0.6 Hz, 1H), 7.94–8.01 (m, 2H), 7.71–7.79 (m, 2H). ^13^C-NMR (100 MHz, MeOH-d4): *δ* 183.2, 148.9, 147.5, 141.7, 138.5, 138.4, 133.4, 133.0, 127.9, 127.8, 127.0, 125.6, 124.4, 123.0, 122.1, 117.3, 116.7, 116.4. HRMS-ESI, *m*/*z*: [M]^+^ calculated for C_18_H_10_^79^BrN_2_O^+^ 348.9974, found 348.9978.

For 12,13-dihydro-9-iodo-13-oxopyrido[1,2-*a*:3,4-*b*’]diindol-5-ium chloride (**7**): prepared at 220 °C, 0.5 h, red solid, 58%. ^1^H (MeOH–d_4_): *δ* 9.36 (s, 1H), 8.93 (s, 1H), 8.87 (s, 1H), 8.32 (d, *J* = 7.5 Hz, 1H), 8.12 (d, *J* = 8.5 Hz, 1H), 8.03 (d, *J* = 7.2 Hz, 1H), 7.96 (dd, *J* = 7.0, 6.2 Hz, 1H), 7.74 (dd, *J* = 7.0, 6.2 Hz, 1H), 7.61 (d, *J* = 8.6 Hz, 1H). ^13^C (100 MHz, MeOH-d4): *δ* 181.7, 147.3, 146.4, 142.4, 139.9, 136.9, 132.7, 131.5, 131.4, 126.5, 125.5, 124.1, 122.0, 120.5, 118.0, 115.2, 115.0. HRMS-ESI, *m*/*z*: [M]^+^ calculated for C_18_H_10_IN_2_O^+^ 396.9832, found 396.9856.

For 3,10-dibromofascaplysin (**8**): prepared at 220 °C, 0.5 h, red solid, 91%. ^1^H NMR (400 MHz, MeOH-d4): δ 9.38 (d, *J* = 6.4, 1H, H-6), 8.97 (d, *J* = 6.4, 1H, H-7), 8.69 (br. s., 1H, H-4), 8,41 (d, *J* = 8.8, 1H, H-8), 8.05 (d, *J* = 1.4, 1H, H-11), 7.97 (d, *J* = 0.8, 2H, H-1, H-2), 7.71 (dd, *J* = 8.6, 1.7, 1H, H-9). ^13^C NMR (100 MHz, MeOH-d4): *δ* 180.2, 147.7, 147.6, 140.8, 134.0, 131.4, 130.7, 128.7, 126.6, 126.4, 126.0, 124.8, 122.7, 119.5, 119.5, 118.7, 118.4, 115.9. ^13^C-NMR (100 MHz, DMSO-d-6): *δ* 181.3, 148.0, 147.8, 140.2, 134.4, 131.2, 130.5, 128.2, 127.7, 127.1, 126.6, 126.1, 123.5, 123.3, 120.8, 119.6, 118.6, 116.4. HRMS-ESI, *m*/*z*: [M]^+^ calculated for C_18_H_9_^79^Br_2_N_2_O^+^ 426.9079, found 426.9085.

For 12,13-dihydro-3,8-dibromo-13-oxopyrido[1,2-*a*:3,4-*b*’]diindol-5-ium chloride (**9**): prepared at 220 °C, 0.5 h, red solid, 93%. ^1^H NMR (400 MHz, MeOH-d4): *δ* 9.44 (d, *J* = 4.7 Hz, 1 H), 9.36 (d, *J* = 4.5 Hz, 1H), 8.76 (s, 1H), 7.98 (s, 2H), 7.76–7.88 (m, 3H). ^13^C NMR (100 MHz, MeOH-d4): *δ* 180.1, 148.1, 147.5, 140.1, 134.5, 134.2, 131.4, 130.7, 126.8, 126.0, 122.7, 122.4, 120.3, 119.5, 118.9, 118.9, 118.5, 112.2. HRMS-ESI, *m*/*z*: [M]^+^ calculated for C_18_H_9_^79^Br_2_N_2_O^+^ 426.9079, found 426.9083.

For 12,13-dihydro-2,9-dibromo-13-oxopyrido[1,2-*a*:3,4-*b*’]diindol-5-ium chloride (**10**): prepared at 200 °C, 1 h, red solid, 88%. ^1^H NMR (400 MHz, MeOH-d4): *δ* 9.39 (br.s., 1H), 8.96 (br.s., 1H), 8.70 (br.s., 1H), 8.40 (d, *J* = 8.0, 1H), 8.00 (s. 1H), 7.90–7.94 (m, 2H), 7.67 (d, *J* = 7.6 Hz, 1H). ^13^C NMR (100 MHz, MeOH-d4): *δ* 180.6, 148.1, 148.0, 141.2, 134.5, 131.9, 131.1, 129.1, 127.1, 126.8, 126.4, 125.3, 123.1, 120.0, 119.2, 118.8, 116.3, 116.0. HRMS-ESI, *m*/*z*: [M]^+^ calculated for C_18_H_9_^79^Br_2_N_2_O^+^ 426.9079, found 426.9088.

For 12,13-dihydro-2-bromo-9-iodo-13-oxopyrido[1,2-*a*:3,4-*b*’]diindol-5-ium chloride (**11**): prepared at 200 °C, 1 h, red solid, yield 54%. ^1^H NMR (400 MHz, DMSO-d6): *δ* 13.68 (br. s., 1H), 9.71 (d, *J* = 6.2 Hz, 1H), 9.18 (d, *J* = 6.2 Hz, 1H), 9.02 (s, 1H), 8.44 (d, *J* = 8.5 Hz, 1H), 8.29 (d, *J* = 1.6 Hz, 1H), 8.24 (dd, *J* = 8.5, 1.6 Hz, 1H), 8.12 (dd, *J* = 8.7, 1.1 Hz, 1H), 7.61 (d, *J* = 8.7 Hz, 1H). ^13^C (100 MHz, DMSO-d6): *δ* 181.6, 169.0, 146.6, 142.7, 139.9, 139.7, 133.5, 131.4, 128.9, 127.9, 126.7, 125.4, 124.9, 123.8, 122.5, 121.8, 118.4, 118.2, 116.6. HRMS-ESI, *m*/*z*: [M]^+^ calculated for C_18_H_9_^79^BrIN_2_O^+^ 474.8937, found 474.8961.

For 12,13-dihydro-2-bromo-9-chloro-13-oxopyrido[1,2-*a*:3,4-*b*’]diindol-5-ium chloride (**12**): prepared at 200 °C, 1 h, red solid, 66%. ^1^H NMR (400 MHz, MeOH-d4): *δ* 9.38 (d, *J* = 6.0 Hz, 1H), 8.98 (d, *J* = 6.0 Hz, 1H), 8.70 (s, 1H), 8.55 (d, *J* = 1.5 Hz, 1H), 7.94 (m, 2H), 7.87 (dd, *J* = 8.9, 1.5 Hz, 1H), 7.79 (d, *J* = 8.9 Hz, 1H). ^13^C NMR (101 MHz, MeOH-d4): *δ* 180.8, 148.3, 146.1, 140.8, 134.8, 132.7, 131.3, 131.1, 129.0, 126.8, 126.6, 123.5, 123.3, 121.1, 120.7, 119.4, 116.2, 114.9. HRMS-ESI, *m*/*z*: [M]^+^ calculated for C_18_H_9_^79^Br^35^ClN_2_O^+^ 382.9581, found 382.9608.

For 12,13-dihydro-2,9,11-tribromo-13-oxopyrido[1,2-*a*:3,4-*b*’]diindol-5-ium chloride (**13**): prepared at 200 °C, 2 h, red solid, 64%. ^1^H NMR (400 MHz, MeOH-d4): *δ* 9.50 (br. s., 1H), 9.05 (br. s., 1H), 8.77 (s, 1H), 8.74 (s, 1H), 8.26 (s, 1H), 7.98 (s, 2H). ^13^C NMR (101 MHz, MeOH-d4): *δ* 180.3, 148.1, 139.2, 135.0, 132.3, 131.5, 129.9, 128.0, 127.4, 126.8, 126.0, 125.0, 123.2, 123.0, 121.6, 119.6, 116.2, 116.0. HRMS-ESI, *m*/*z*: [M]^+^ calculated for C_18_H_8_^79^Br_3_N_2_O^+^ 504.8181, found 504.8209.

For 12,13-dihydro-2-bromo-9,11-dichloro-13-oxopyrido[1,2-*a*:3,4-*b*’]diindol-5-ium chloride (**14**): prepared at 200 °C, 1 h, red solid, 47%. ^1^H NMR (400 MHz, MeOH-d4): *δ* 9.46 (d., *J* = 6.2 Hz, 1H), 9.05 (d, *J* = 6.1 Hz, 1H), 8.71 (s., 1H), 8.55 (d, *J* = 1.7 Hz, 1H), 7.99 (d, *J* = 1.7 Hz, 1H), 7.95 (s, 2H). ^13^C NMR (101 MHz, MeOH-d4): *δ* 179.7, 147.6, 142.7, 140.7, 134.5, 133.1, 132.0, 131.0, 128.5, 127.3, 126.2, 122.7, 122.1, 121.8, 121.4, 121.0, 119.0, 115.7. HRMS-ESI, *m*/*z*: [M]^+^ calculated for C_18_H_8_^79^Br^35^Cl_2_N_2_O^+^ 416.9192, found 416.9221.

For 12,13-dihydro-3,9-dibromo-13-oxopyrido[1,2-*a*:3,4-*b*’]diindol-5-ium chloride (**15**): prepared at 200 °C, 2 h, red solid, 64%. ^1^H NMR (400 MHz, DMSO-d6): *δ* 13.73 (br. s., 1H), 9.69 (s, 1H), 9.19 (s, 1H), 8.91 (s, 1H), 8.85 (s, 1H), 7.95 (m, 3H), 7.74 (d, *J* = 7.6 Hz, 1H). ^13^C NMR (101 MHz, DMSO-d6): *δ* 181.6, 148.3, 146.2, 140.0, 139.8, 137.1, 134.8, 131.5, 131.3, 130.9, 127.7, 127.3, 124.2, 123.7, 121.5, 120.0, 116.3, 115.5. HRMS-ESI, *m*/*z*: [M]^+^ calculated for C_18_H_9_^79^Br_2_N_2_O^+^ 426.9079, found 426.9085.

For 12,13-dihydro-6-methyl-13-oxopyrido[1,2-*a*:3,4-*b*’]diindol-5-ium chloride (**19**): prepared at 220 °C, 0.5 h, red solid, 93%. ^1^H NMR (400 MHz, MeOH-d4): *δ* 8.76 (s, 1H), 8.37 (d, *J* = 8.2 Hz, 2H), 8.07 (d, *J* = 7.2 Hz, 1H), 7.98 (t, *J* = 7.7 Hz, 1H), 7.82 (t, *J* = 7.7 Hz, 1H), 7.76 (t, *J* = 7.4 Hz, 1H), 7.70 (d, *J* = 7.7 Hz, 1H), 7.45 (t, *J* = 7.4 Hz, 1H), 3.40 (s, 3H). ^13^C NMR (100 MHz, MeOH-d4): *δ* 183.8, 150.0, 149.6, 145.1, 142.8, 138.6, 136.1, 132.8, 132.3, 127.2, 126.6, 125.5, 124.6, 124.5, 124.0, 121.6, 120.6, 114.6, 23.4. HRMS-ESI, *m*/*z*: [M]^+^ calculated for C_19_H_13_N_2_O^+^ 285.1022, found 285.1039.

For 12,13-dihydro-6-phenyl-13-oxopyrido[1,2-*a*:3,4-*b*’]diindol-5-ium chloride (**20**): prepared at 220 °C, 0.5 h, red solid, 97%. ^1^H NMR (400 MHz, MeOH-d4): *δ* 8.86 (s, 1H), 8.46 (d, *J* = 8.1 Hz, 1H), 8.06 (dd, *J1* = 7.4 Hz, *J2*= 0.9 Hz, 1H), 7.77–7.94 (m, 7H), 7.62 (dd, *J1* = *J2* = 7.7 Hz, 1H), 7.49–7.64 (m, 2H), 6.49 (d, *J* = 8.4 Hz, 1H). ^13^C NMR (100 MHz, MeOH-d4): *δ* 183.8, 149.9, 149.8, 144.1, 142.6, 137.6, 136.3, 134.1, 133.1, 132.2, 131.1, 130.8, 126.9, 126.7, 125.7, 124.7, 124.5, 124.4, 121.2, 120.4, 114.8, 113.5. HRMS-ESI, *m*/*z*: [M]^+^ calculated for C_24_H_15_N_2_O^+^ 347.1179, found 347.1196.

#### 3.1.9. General Procedure for the Condensation of Indigo with Methylene Active Compounds

Sodium hydride (75 mg, 60% dispersion in mineral oil) was added to a suspension of powdered indigo (131 mg, 0.5 mmol) in dry DMF (10 mL). When the evolution of hydrogen was finished, the methylene active compound (4 mmol) was added gradually. The reaction mixture was heated at reflux and stirred for 30 min, then cooled, diluted with H_2_O (50 mL), acidified with hydrochloric acid and filtered. The solid residue was washed with Et_2_O (2 × 3 mL) and dried. The crude product was purified by MPLC using chloroform as an eluent. Compound samples for recording NMR and mass spectra were repurified by the Shimadzu HPLC system.

For 7-phenyl-6,13-dioxopyrido[1,2-*a*:3,4-*b*’]diindole (**32a**): dark purple powder, 55%. ^1^H NMR (400 MHz, 50 °C, DMSO-d_6_): *δ* 11.65 (s, 1H), 8.59 (d, *J* = 8.1 Hz, 1H), 7.82 (d, *J* = 7.5 Hz, 1H), 7.72 (t, *J* = 7.8 Hz, 1H), 7.51–7.63 (m, 5H), 7.37–7.44 (m, 3H), 6.80–6.89 (m, 2H). ^13^C NMR (100 MHz, 50 °C, DMSO-d_6_): *δ* 179.6, 156.3, 147.4, 145.9, 137.8, 135.3, 134.0, 133.7, 131.4, 129.3, 129.0, 128.6, 128.3, 125.9, 124.7, 123.7, 123.3, 120.5, 119.4, 117.3, 114.5, 112.3. HRMS (ESI^+^): *m*/*z* calculated for C_24_H_14_N_2_O_2_Na^+^ [M + Na]^+^ 385.0953, found 385.0958.

For 7-ethyl-6,13-dioxopyrido[1,2-*a*; 3,4-*b*’]diindole (**32b**): dark purple powder, 50%. ^1^H NMR (400 MHz, DMSO-d_6_): *δ* 11.48 (br. s, 1H), 8.62 (d, *J* = 8.2 Hz, 1H), 7.98 (d, *J* = 7.9 Hz, 1H), 7.77 (d, *J* = 7.4 Hz, 1H), 7.70 (m, 1H), 7.50 (t, *J* = 7.5 Hz, 1H), 7.43 (d, *J* = 7.9 Hz, 1H), 7.37 (t, *J* = 7.5 Hz, 1H), 7.19 (t, *J* = 7.5 Hz, 1H), 3.11 (q, *J* = 7.5 Hz, 2H), 1.26 (t, *J* = 7.5 Hz, 3H). ^13^C NMR (100 MHz, DMSO-d_6_): *δ* 180.3, 170.0, 157.5, 147.8, 146.6, 139.4, 137.3, 135.9, 131.9, 129.9, 126.8, 125.8, 125.7, 124.2, 122.2, 120.4, 118.2, 113.0, 20.9, 12.8. HRMS (ESI^+^): *m*/*z* calculated for C_20_H_14_N_2_O_2_Na^+^ [M + Na]^+^ 337.0952, found 337.1121.

For 7-methyl-6,13-dioxopyrido[1,2-*a*; 3,4-*b*’]diindole (**32c**): dark purple powder, 61%. ^1^H NMR (400 MHz, DMSO-d_6_): *δ* 11.56 (br.s., 1H), 8.62 (d, *J* = 8.2 Hz, 1H), 8.02 (d, *J* = 7.9 Hz, 1H), 7.75 (d, *J* = 7.5 Hz, 1H), 7.68 (dd, *J* = 7.6 Hz, 1H), 7.48 (dd, *J* = 7.4 Hz, 1H), 7.41 (d, *J* = 7.9 Hz, 1H), 7.34 (dd, *J* = 7.4 Hz, 1H), 7.17 (dd, *J* = 7.6 Hz, 1H), 2.58 (s, 3H). ^13^C NMR (100 MHz, 50 °C, DMSO-d_6_): *δ* 182.3, 164.3, 157.8, 147.5, 146.5, 135.7, 133.4, 131.5, 131.4, 126.5, 125.7, 125.6, 123.9, 123.0, 121.8, 121.0, 118.0, 112.7, 13.8. HRMS (ESI^+^): *m*/*z* calculated for C_19_H_12_N_2_O_2_Na^+^ [M + Na]^+^ 323.0796, found 323.0815.

#### 3.1.10. Preparation of Compounds **16**–**18**

A dry 50 mL flask equipped with a magnetic stirring bar and a reflux condenser was cooled to 0 °C and then charged with NaBH_4_ (135 mg, 4.0 mmol) followed by sequential additions of dry THF (10 mL) and BF_3_∙Et_2_O (564 µL, 4.4 mmol) at 0 °C. The ice bath was removed, and contents were stirred at RT for 15 min with subsequent addition of compound **32a**–**c** (0.17 mmol). The reaction mixture was heated at reflux under argon for 2 h (for **16**) or was placed in a sealed 10 mL vial and irradiated using a microwave synthesis system at 50 W for 30 min under argon (for **17** and **18**). After cooling to room temperature, the mixture was cautiously acidified, diluted by water and refluxed again for 2 h. After cooling to room temperature, the reaction was extracted with CHCl3. Then, the water phase was separated and brought to 8–9 pH with aqueous ammonia (mixture color changed from red to green) and extracted with CHCl_3_ (3 × 20 mL). The combined organic layer was filtered, acidified with hydrochloric acid (color changed from green to red), evaporated under reduced pressure and dried to give fascaplysin derivative as a dark red water-soluble powder.

For 12,13-dihydro-7-phenyl-13-oxopyrido[1,2-*a*:3,4-*b*’]diindol-5-ium chloride (**16**): red powder, 44%. ^1^H NMR (400 MHz, MeOH-d4): *δ* 9.34 (s, 1H), 8.38 (d, *J* = 8.0 Hz, 1H), 8.05 (d, *J* = 7.4 Hz, 1H), 7.94 (t, *J* = 7.4 Hz, 1H), 7.79–7.85 (m, 4H), 7.72–7.76 (m, 4H), 7.63 (d, *J* = 8.2 Hz, 1H), 7.25 (ddd, *J* = 8.2, 5.0, 3.1 Hz, 1H). ^13^C NMR (100 MHz, MeOH-d4): *δ* 181.9, 169.0, 147.7, 147.4, 138.2, 137.7, 137.0, 134.3, 133.8, 132.2, 131.6, 130.5, 129.5, 129.2, 126.5, 125.6, 124.6, 124.5, 122.9, 121.2, 119.7, 115.6, 113.6. HRMS (ESI^+^): *m*/*z* calculated for C_24_H_15_N_2_O M^+^ 347.1181, found 347.1185.

For 12,13-dihydro-7-ethyl-13-oxopyrido[1,2-*a*:3,4-*b*’]diindol-5-ium chloride (**17**): red powder, 35%. ^1^H NMR (400 MHz, MeOH-d4): *δ* 8.80 (s, 1H), 8.56 (d, *J* = 7.5 Hz, 1H), 8.38 (d, *J* = 7.4 Hz, 1H), 8.10 (d, *J* = 7.2 Hz, 1H), 8.00 (t, *J* = 7.8 Hz, 1H), 7.84 (t, *J* = 7.9 Hz, 1H), 7.78 (t, *J* = 7.9 Hz, 1H), 7.73 (d, *J* = 7.2 Hz, 1H), 7.48 (t, *J* = 7.7 Hz, 1H), 3.29 (q, *J* = 8.1 Hz, 2H), 1.26 (t, *J* = 7.8 Hz, 3H). ^13^C NMR (100 MHz, DMSO-d_6_): *δ* 181.9, 147.7, 147.4, 137.7, 137.0, 134.3, 133.8, 131.6, 130.5, 129.5, 129.2, 126.5, 125.6, 124.5, 122.9, 119.7, 115.6, 113.6, 20.48, 15.8. HRMS-ESI, *m*/*z*: [M]^+^ calculated for C_20_H_15_N_2_O^+^ 299.1179, found 299.1194.

For 12,13-dihydro-7-methyl-13-oxopyrido[1,2-*a*:3,4-*b*’]diindol-5-ium chloride (**18**): red powder, 53%. ^1^H NMR (400 MHz, MeOH-d4): *δ* 9.35 (s, 1H), 8.47 (d, *J* = 8.2 Hz, 1H), 8.35 (d, *J* = 8.1 Hz, 1H), 8.03 (d, *J* = 7.3 Hz, 1H), 7.96 (t, *J* = 7.8 Hz, 1H), 7.88 (dd, *J* = 7.1 Hz, 1H), 7.83 (d, *J* = 8.2 Hz, 1H), 7.73 (t, *J* = 7.6 Hz, 1H), 7.56 (t, *J* = 7.5 Hz, 1H), 3.17 (s, 3H). ^13^C NMR (100 MHz, MeOH-d4): *δ* 181.8, 148.6, 147.2, 147.0, 139.1, 137.0, 136.6, 135.0, 133.5, 131.2, 126.4, 125.3, 124.4, 123.2, 120.3, 115.0, 113.2, 112.0, 17.0. HRMS-ESI, *m*/*z*: [M]^+^ calculated for C_19_H_13_N_2_O^+^ 285.1022, found 285.1040.

### 3.2. Biological Assay

#### 3.2.1. Reagents

MTT (3-(4,5-dimethylthiazol-2-yl)-2,5-diphenyltetrazolium bromide) and propidium iodide (PI) from Sigma (Taufkirchen, Germany), thiazole orange from Merck KGaA, Germany, RNase from Carl Roth (Karlsruhe, Germany).

#### 3.2.2. Cell Lines and Culture Conditions

The human prostate cancer cell lines PC-3, DU145, 22Rv1 and LNCaP, as well as human prostate non-cancer cell line PNT2 were purchased from ATCC (Manassas, VA, USA). HEK 293T (human embryonic kidney cells) and MRC-9 (human fibroblast cells) cell lines were purchased from ECACC (Salisbury, UK). All the cells had a passage number ≤ 30. Authentication of all cell lines used was recently performed by a commercial service (Multiplexion GmbH, Heidelberg, Germany).

Cells were cultured as monolayers at 37 °C in a humidified atmosphere with 5% (*v*/*v*) CO_2_ in the correspondent culture medium: 10% FBS/RPMI medium (RPMI medium supplemented with Glutamax^TM^-I (gibco^®^ Life technologies^TM^, Paisley, UK) containing 10% fetal bovine serum (FBS, gibco^®^ Life technologies^TM^) and 1% penicillin/streptomycin (Invitrogen)) for PNT2, LNCaP, 22Rv1, PC-3 and DU145 and cells; 10% FBS/DMEM medium (DMEM medium supplemented with Glutamax^TM^-I (gibco^®^ Life technologies^TM^) containing 10% FBS and 1% penicillin/streptomycin (gibco^®^ Life technologies^TM^)) for MRC-9 and HEK 293 cells. Cells were continuously kept in culture for a maximum of 3 months, and were regularly checked for stable phenotype and mycoplasma infection.

#### 3.2.3. MTT Assay

Effect of the drugs on cell viability in terms of metabolic activity was assessed by MTT assay, as previously reported [50]. Cells were pre-incubated overnight in 96-well plates (6 × 10^3^ cells/well in 100 μL/well) in the correspondent culture media. Then, the medium was replaced with fresh 10% FBS/RPMI medium containing the investigated compounds (100 μL/well), and cells were incubated for 48 h, unless otherwise stated. Next, 10 μL/well of (3-(4,5-dimethylthiazol-2-yl)-2,5-diphenyltetrazolium bromide) reagent (MTT, 5 mg/mL) was added. After 2–4 h of incubation, the media was aspirated and the plates were dried. Then, 50 μL/well of DMSO was added to each well and the cell viability was measured using an Infinite F200PRO reader (TECAN, Männedorf, Switzerland). Results were calculated using the GraphPad Prism software v.9.1.1 (GraphPad Software, San Diego, CA, USA) and are represented as IC_50_ of the compounds calculated using the cells treated with the solvent alone as a control.

#### 3.2.4. In Vitro Trypan Blue-Based Viability Assay

The effect of the drugs on cell viability in terms of cellular membrane integrity was evaluated by the trypan blue exclusion assay as described before [50]. In brief, cells (0.2 × 10^6^ cells/well) were seeded in 6-well plates and incubated overnight, and the media was replaced with fresh media (1 mL/well) containing drugs at indicated concentrations. After treatment for 48 h, the cells were harvested by trypsinization, stained with trypan blue and the viability was measured using an automatic Beckman Coulter Vi-CELL (Beckman Coulter, Krefeld, Germany). Trypan blue-positive cells were assumed to have damaged cellular membrane (i.e., dead), whereas trypan blue-negative cells were considered to have an intact cellular membrane (i.e., alive).

#### 3.2.5. Analysis of Cell Cycle Progression and DNA Fragmentation

The effects on cell cycle progression and DNA fragmentation were analyzed by flow cytometry using PI staining, as described before [51]. Cells (2 × 10^5^ cells/well) were pre-incubated overnight in 6-well plates and then treated with the investigated drugs in fresh culture media (2 mL/well) for 48 h. Cells were harvested by trypsinization, fixed in 70% EtOH, stained with PI solution and analyzed by FACS Calibur (BD Bioscience, San Jose, CA, USA). The results were quantified using BD Bioscience Cell Quest Pro v.5.2.1. software (BD Bioscience). The cells appearing as a sub-G1 population (containing fragmented DNA) were considered to be apoptotic.

#### 3.2.6. Thiazole Orange Displacement (DNA Intercalation) Assay

DNA intercalation activity of the compounds was calculated as an ability to displace thiazole orange (TO) from the double-stranded DNA. For this experiment, a mixture (100 µL) containing 1 µM of double-stranded calf thymus DNA (recalculated as a concentration of base pairs) and 2 µM of thiazole orange (TO) solved in water was prepared. Then, the investigated compounds were added up to final concentrations of 0.39–25 µM; DMSO concentration in the samples was <0.02%. Propidium iodide (PI, positive control) was added up to final concentrations of 0.039–2.5 uM. After 7 min of incubation at room temperature, TO fluorescence was monitored using a multimodal plate reader TECAN Spark (Tecan Group Ltd., Männedorf Switzerland) at the excitation λ = 480 nm; fluorescence was recorded at λ = 530 nm. The bandwidth was 10 nm. The concentration of the drug that caused a decrease in TO fluorescence by 50% (EC_50_) was determined by approximating the experimental data using a non-linear regression algorithm and calculated using the GraphPad Prism software v.9.1.1 (GraphPad Software, San Diego, CA, USA).

#### 3.2.7. Data and Statistical Analysis

For statistical analyses of the data, GraphPad Prism software v.9.1.1 (GraphPad Software, San Diego, CA, USA) was used. Data are presented as mean ± SD (standard deviation). The unpaired Student’s *t*-test or one-way ANOVA followed by Dunnett’s post-hoc tests were used for comparison of two or several groups, correspondingly. Dunnett’s post-hoc test was used to compare multiple treatments with a single control. All the experiments were performed in triplicates (*n* = 3, biological replicates) unless otherwise stated. Differences were considered to be statistically significant (*) if *p* < 0.05.

## 4. Conclusions

In conclusion, we have synthesized a library of various derivatives of the marine alkaloid fascaplysin and determined their anticancer potential. In the current research, for the very first time, the derivatives covering all the possible positions for fascaplysin modifications (namely, substituent introduction only to the cycles A, C or E) have been reported and analyzed. The highest, well-pronounced selectivity towards prostate cancer cells has been observed for the family of di- and trisubstituted halogen derivatives (modification of the cycles A and E). At the same time, an introduction of a small alkyl (methyl or ethyl) or phenyl radical to the cycle C resulted in stronger activity in treatment-resistant PC-3 cells, which has not been observed for the other types of synthesized derivatives. Of note, 3,10-dibromofascaplysin exhibited the highest selectivity, presumably due to the cytostatic effects executed via cellular metabolism targeting. Finally, we have shown that an introduction of bromine at C-9, C-10 or C-10 plus C-3 leads to a notable reduction in DNA intercalating activity. This was accompanied by a meaningful reduction in the unfavorable cytotoxicity to non-cancer cells and resulted in higher drug selectivity. Taken together, our studies make a significant contribution to understanding the structure–activity relationships of fascaplysin derivatives and indicate the need to expand and study the series of bulky alkyl (e.g., *tert*-butyl) and aryl fascaplysin derivatives. It also seems promising to introduce halogen atoms to the rings A und E of fascaplysin at the positions C-3 and C-10.

## Figures and Tables

**Table 1:** The structures of the synthesized fascaplysin derivatives.

Compound	X1	R	X2
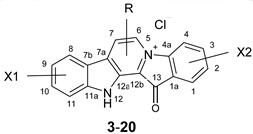			
**1**	-	-	-
**3**	-	-	3-Br
**4**	-	-	2-Br
**5**	10-Br	-	-
**6**	9-Br	-	-
**7**	9-I	-	-
**8**	10-Br	-	3-Br
**9**	8-Br	-	3-Br
**10**	9-Br	-	2-Br
**11**	9-I	-	2-Br
**12**	9-Cl		2-Br
**13**	9, 11-Br	-	2-Br
**14**	9, 11-Cl	-	2-Br
**15**	9-Br	-	3-Br
**16**	-	7-Ph	-
**17**	-	7-Et	-
**18**	-	7-Me	-
**19**	-	6-Me	-
**20**		6-Ph	

**Table 2:** The direct iodination of fascaplysin under various reaction conditions.

Experiment.	Heating (°C) or MW	Time, min	Reactant Ratio			Reaction Results
			1, mmol	I_2_, equiv.	DMSO, mL	
1	120	280	0.011	18.8	1	mixture of **1** and **7**
2	120	240	0.007	28	0.5	mixture of **1** and **7**
3	120	120	0.007	28	1	decomposition of the mixture
4	MW, 50 W	18	0.04	5.3	1	mixture of **1** and **7** (50/50)
5	MW, 50 W	28	0.04	5.3	1	15 min—**7** found, 28 min—decomposition of the mixture
6	MW, 45 W	30	0.04	5.3	1 (autoclave)	mixture of **1** and **7** (minor)
7	MW, 45 W	30	0.04	5.3	2	no reaction
8	MW, 45 W	30	0.01	18.8	1	mixture of **1** and **7** (minor)

## Data Availability

The data presented in this study are available on request from the corresponding author.

## Supplementary Materials

The following supporting information can be downloaded at: https://www.mdpi.com/article/10.3390/md20030185/s1, Table S1: Pro-apoptotic and anti-proliferative activity of the synthesized compounds in cancer and non-cancer cells; Table S2: Activity and selectivity of the synthesized compounds in cancer versus non-cancer cell lines. Cisplatin (Cis) was used as a positive control; Spectra data.
