# Supplementary material for: Study of Structure–Activity Relationships of the Marine Alkaloid Fascaplysin and Its Derivatives as Potent Anticancer Agents
Source: Mar Drugs. 2022 Mar 2;20(3):185. doi: 10.3390/md20030185 (PMC8949187; doi:10.3390/md20030185)

Supporting Information

Comprehensive study of structure-activity relationships of the marine alkaloid fascaplysin and its derivatives as potent anticancer agents

Maxim E. Zhidkov [1,*], Moritz Kaune [2], Alexey V. Kantemirov [1], Polina A. Smirnova [1], Pavel V. Spirin [3,4], Maria A. Sidorova [1], Sergey A. Stadnik [1], Elena Y. Shyrokova [3,5], Dmitry N. Kaluzhny [6], Oleg A. Tryapkin [1], Tobias Busenbender [2], Jessica Hauschild [2], Tina Rohlfing [2], Vladimir S. Prassolov [3,4], Carsten Bokemeyer [2], Markus Graefen [7], Gunhild von Amsberg [2,7] and Sergey A. Dyshlovoy [2,7,8,*]

[1] Department of chemistry and materials, Institute of high technologies and advanced materials, Far Eastern Federal University, FEFU Campus, Ajax Bay 10, Russky Island, 690922 Vladivostok, Russia;
[2] Department of Oncology, Hematology and Bone Marrow Transplantation with Section Pneumology, Hubertus Wald-Tumorzentrum, University Medical Center Hamburg-Eppendorf, Martinistrasse 52, 20246 Hamburg, Germany
[3] Department of Cancer Cell Biology, Engelhardt Institute of Molecular Biology, Russian Academy of Sciences, Vavilova 32, 119991 Moscow, Russia
[4] Center for Precision Genome Editing and Genetic Technologies for Biomedicine, Engelhardt Institute of Molecular Biology, Russian Academy of Sciences, Vavilova 32, 119991 Moscow, Russia.
[5] Moscow Institute of Physics and Technology (National Research University), Institutskiy Per. 9, 141701 Dolgoprudny, Russia.
[6] Laboratory of DNA protein interaction, Engelhardt Institute of Molecular Biology, Russian Academy of Sciences, Vavilova 32, 119991 Moscow, Russia
[7] Martini-Klinik Prostate Cancer Center, University Hospital Hamburg-Eppendorf, Martinistrasse 52, 20246 Hamburg, Germany
[8] Laboratory of Pharmacology, A.V. Zhirmunsky National Scientific Center of Marine Biology, Palchevskogo str. 17, 690041 Vladivostok, Russian Federation

* Correspondence: zhidkov.me@dvfu.ru; and s.dyshlovoy@uke.de

Contents

Table S1. Pro-apoptotic and anti-proliferative activity of the synthesized compounds in cancer and non-cancer cells. Cisplatin (Cis) was used as a positive control 3

Table S2. Activity and selectivity of the synthesized compounds in cancer versus non-cancer cell lines. Cisplatin (Cis) was used as a positive control 4

Spectra Data 5-72

Table S 1. Pro-apoptotic and anti-proliferative activity of the synthesized compounds in cancer and non-cancer cells. Cisplatin (Cis) was used as a positive control.

Compound	[a] IC_{50} [µM], MTT assay							Activity in 22Rv1 cells			[e] LogP
	Prostate cancer cells				Non-cancer cells			[b] IC_{50} [µM], trypan blue staining assay	[c] Cell cycle arrest (at IC_{50})	[d] % of apoptotic cells (at IC_{50})	
	PC-3	22Rv1	DU145	LNCaP	PNT2	MRC-9	HEK-293				
**1**	0.766±0.126	0.242±0.81	0.798±0.054	0.409±0.02	0.457±0.075	0.89±0.046	0.458±0.19	0.335±0.093	G1	11.43±0.843	0.28
**3**	10.03±6.75	0.417±0.06	1.51±0.13	1.25±0.38	0.727±0.06	2.3±0.87	1.01±0.071	0.24±0.041	G1	23.3±9.19	1.06
**4**	3.4±1.89	0.529±0.069	1.9±0.634	0.349±0.081	0.82±0.076	4.27±0.662	1.1±0.074	0.26±0.054	G1	8.83±0.7	1.06
**5**	17.82±1.94	0.5±0.067	1.27±0.206	0.46±0.076	0.302±0.09	20.3±5.4	0.649±0.078	0.482±0.091	G1	3.63±0.844	1.06
**6**	2.78±0.37	1.07±0.094	2.24±0.180	1.41±0.089	2.45±0.95	15.5±2.5	2.54±1.14	2.79±0.223	No	32.38±1	1.06
**7**	3.67±0.2	0.55±0.071	0.319±0.075	0.171±0.035	0.145±0.066	0.924±0.132	0.809±0.164	0.08±0.004	G1	6.3±0.906	1.34
**8**	7.28±0.48	0.69±0.019	1.51±0.056	0.593±0.091	3.49±1.01	>50	0.54±0.048	5.14±0.22	No	3.83±0.442	1.85
**9**	1.39±0.7	0.209±0.072	0.334±0.034	0.361±0.085	0.369±0.024	2.07±0.47	0.58±0.099	0.259±0.094	No	28.7±3.49	1.83
**10**	1.83±0.1	0.49±0.039	0.913±0.116	1.13±0.803	0.614±0.098	4.96±0.386	0.25±0.034	0.215±0.036	G1	24.0±5.37	1.85
**11**	3.14±0.98	2.33±0.87	1.35±0.318	1.17±0.084	0.86±0.072	31.9±7.07	0.48±0.052	1.33±0.277	G1	13.1±2.86	2.12
**12**	13.24±2.87	2.89±0.89	2.08±0.58	2.7±0.173	3.33±0.806	36.3±9.51	0.75±0.065	1.64±0.85	No	6.95±0.901	1.72
**13**	5.94±0.86	3.98±0.3	13.2±4.44	7.63±0.74	4.38±0.674	30±5.80	2.25±0.99	4.48±1.65	G1	27.6±7.52	2.59
**14**	5.29±0.132	4.15±0.34	6.91±0.65	6.51±2.32	2.75±1.74	>50	2.61±0.429	7.43±0.85	No	23.6±1.2	2.33
**15**	2.95±0.23	2.24±0.811	0.616±0.094	2.6±0.367	0.643±0.049	12.6±6.94	0.275±0.021	2.85±0.487	G2	8.63±0.71	1.85
**16**	0.391±0.084	0.185±0.039	0.491±0.068	0.141±0.058	0.434±0.021	0.596±0.11	0.538±0.069	0.172±0.074	G1	7±1.4	2.00
**17**	0.564±0.098	0.271±0.046	2.37±0.45	0.611±0.047	0.49±0.09	1.45±0.645	0.7±0.211	0.375±0.09	G1	13.4±0.92	1.12
**18**	1.07±0.55	0.79±0.038	1.79±0.478	0.803±0.08	0.956±0.092	1.18±0.096	0.59±0.085	0.472±0.185	G1	23.1±3.42	0.66
**19**	2.67±0.14	1.59±0.514	2.24±0.89	2.24±0.81	3.25±0.795	2.98±0.93	1.82±0.387	2.03±0.609	G1	14.2±0.567	0.50
**20**	0.429±0.1	0.156±0.032	0.444±0.095	0.283±0.035	0.44±0.075	0.86±0.111	0.412±0.066	0.207±0.074	No	16.4±2.47	2.00
**Cis**	34.6±	0.99±0.29	1.32±0.53	2.75±0.55	9.41±3.12	6.75±1.99	6.44±1.77	0.19±0.044	G2/M	14.7±3.43	N/A

[a,b] Cytotoxic activity of the synthesized compounds determined by MTT assay ([a]) and trypan blue staining assay ([b]). For the trypan blue staining assay 22Rv1 cells were used ([b]). Activity has been represented as IC_{50} [µM] following 48 h of treatment. [c,d] Effect of the synthesized compounds on cell cycle progression ([c]) and DNA fragmentation (apoptosis, [d]) in 22Rv1 cells. Cells were treated with the investigated compounds at the corresponding IC_{50}s (MTT assay, [a]) for 48 h, harvested, fixed, stained and analyzed using a flow cytometry technique. Cells appeared at sub-G1 phase were assumed to have fragmentated DNA and were therefore assigned as apoptotic ([d]). ([e]), Lipophilicity of the synthesized compounds estimated *in silico* using the software ClogP (www.molinspiration.com). Data is represented as mean ± SD.

Table S 2. Activity and selectivity of the synthesized compounds in cancer versus non-cancer cell lines. Cisplatin (Cis) was used as a positive control.

Compound	[a] mean IC_{50}s in cancer cells	Selectivity index		[d] IC_{50} (Trypan blue) / IC_{50} (MTT)
		[b] IC_{50} (non-cancer) / IC_{50} (cancer)	[c] IC_{50} (non-prostate) / IC_{50} (prostate)	
**1**	0.55	1.09	1.26	1.38
**3**	3.30	0.41	0.59	0.58
**4**	1.55	1.34	1.92	0.49
**5**	5.01	1.41	2.57	0.96
**6**	1.88	3.65	4.54	2.61
**7**	1.18	0.53	0.89	0.15
**8**	2.52	7.15	9.32	7.45
**9**	0.57	1.75	2.48	1.24
**10**	1.09	1.78	2.62	0.44
**11**	2.00	5.55	9.15	0.57
**12**	5.23	2.58	3.82	0.57
**13**	7.69	1.59	2.29	1.13
**14**	5.72	3.23	5.14	1.79
**15**	2.10	2.15	3.56	1.27
**16**	0.30	1.73	1.73	0.93
**17**	0.95	0.92	1.25	1.38
**18**	1.11	0.82	0.82	0.60
**19**	2.18	1.23	1.00	1.28
**20**	0.33	1.74	1.82	1.33
**Cis**	3.62	1.23	0.92	0.78

[a] Mean IC_{50} evaluated using MTT assay in four prostate cancer cells lines (i.e. PC-3, 22Rv1, DU145 and LNCaP). [b,c] Selectivity index (SI) calculated as: ratio of mean IC_{50} [non-cancer cell lines] versus mean IC_{50} [cancer cell lines] ([b]) or as mean IC_{50} [non-cancer non-prostate cell lines, i.e. MRC-9 and HEK-293] versus mean IC_{50} [prostate cells, i.e. prostate cancer cell lines plus PNT2 cells] ([c]). Color code: yellow, $2 > SI > 1.5$; red, $SI > 2$. [d] Ratio of IC_{50} [22Rv1 cells, trypan blue assay] to IC_{50} [22Rv1 cells, MTT assay]. The values used for calculations ([a-d]) are corresponding to those shown in Table S 1.

Spectra Data

^{1}H NMR spectra of 1-(2’,4’-dibromobenzoyl)-β-carboline

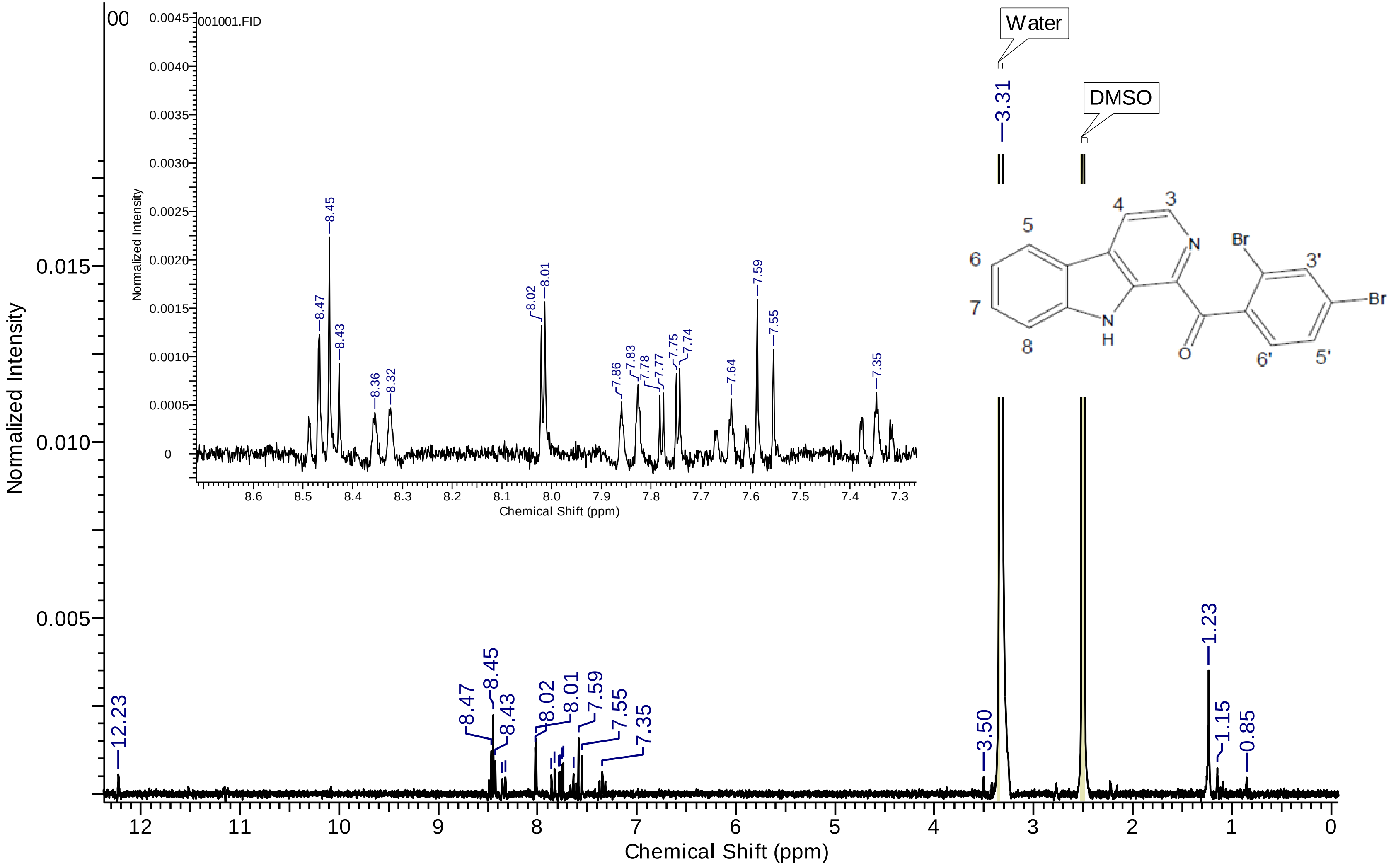

^{13}C NMR spectra of 1-(2',4'-dibromobenzoyl)-β-carboline

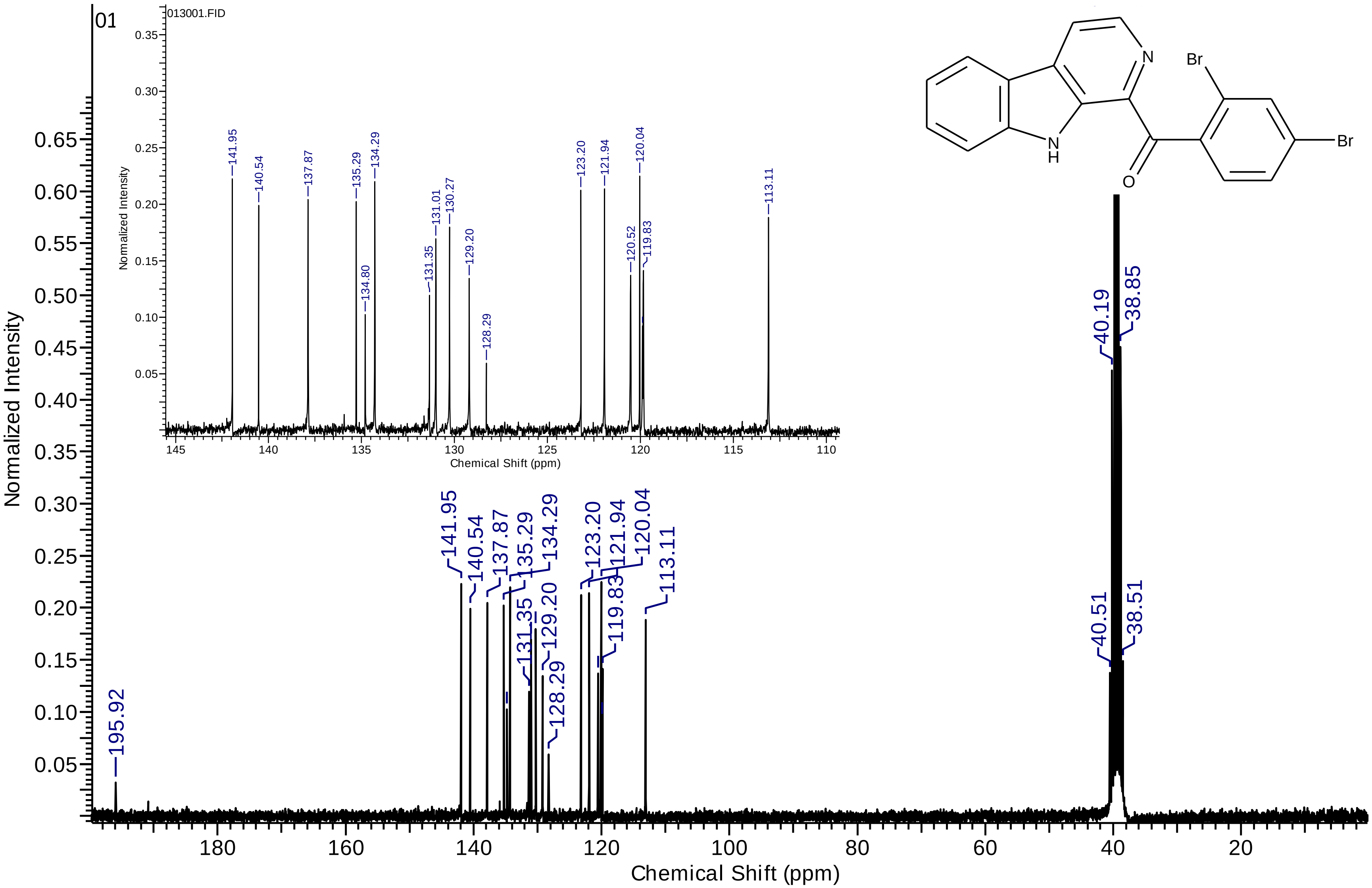

^{1}H NMR spectra of 1-(2’,5’-dibromobenzoyl)-β-carboline

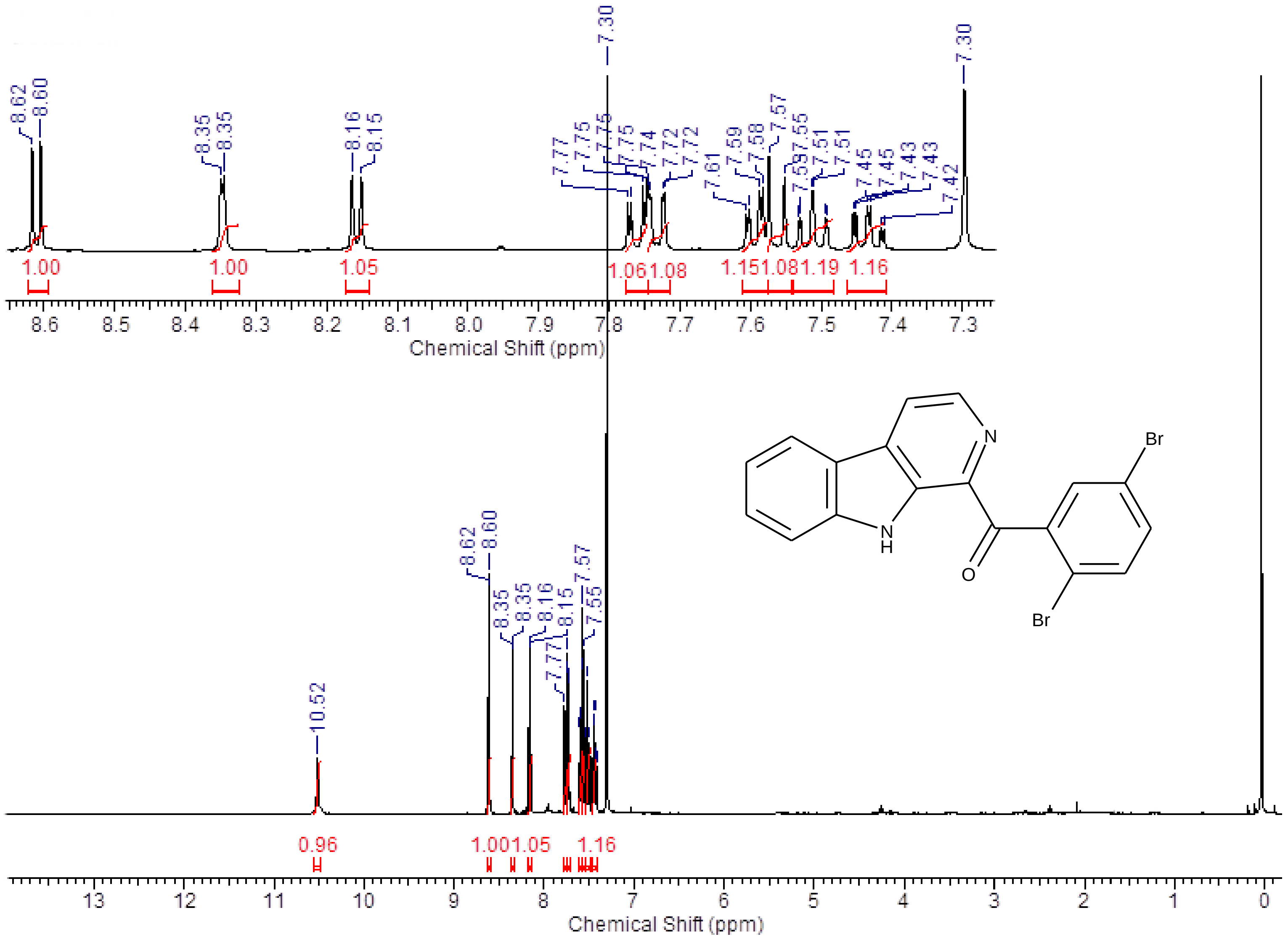

^{13}C NMR spectra of 1-(2’,5’-dibromobenzoyl)-β-carboline

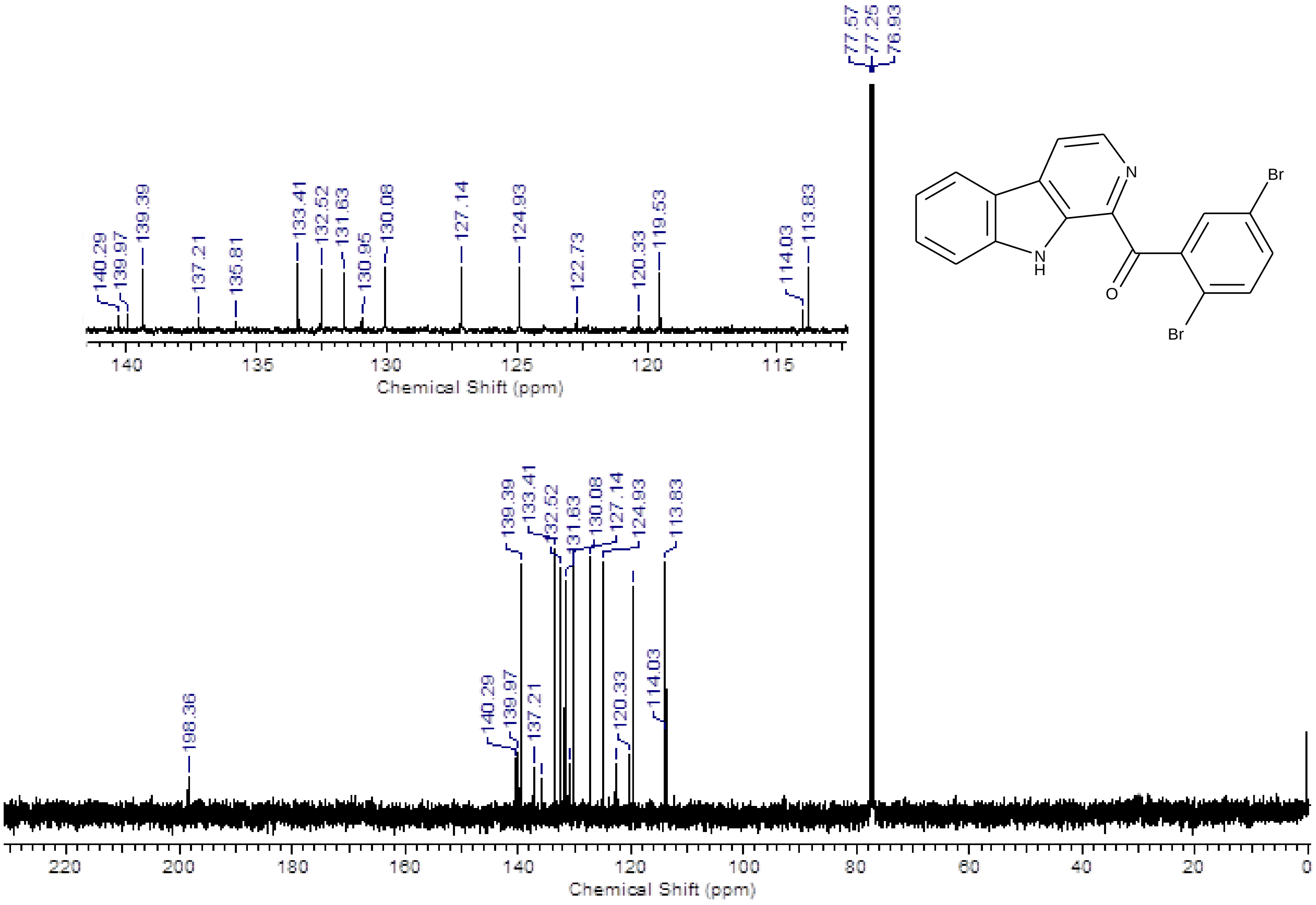

^{1}H NMR spectra of 1-(2’,4’-dibromobenzoyl)-7-bromo-β-carboline

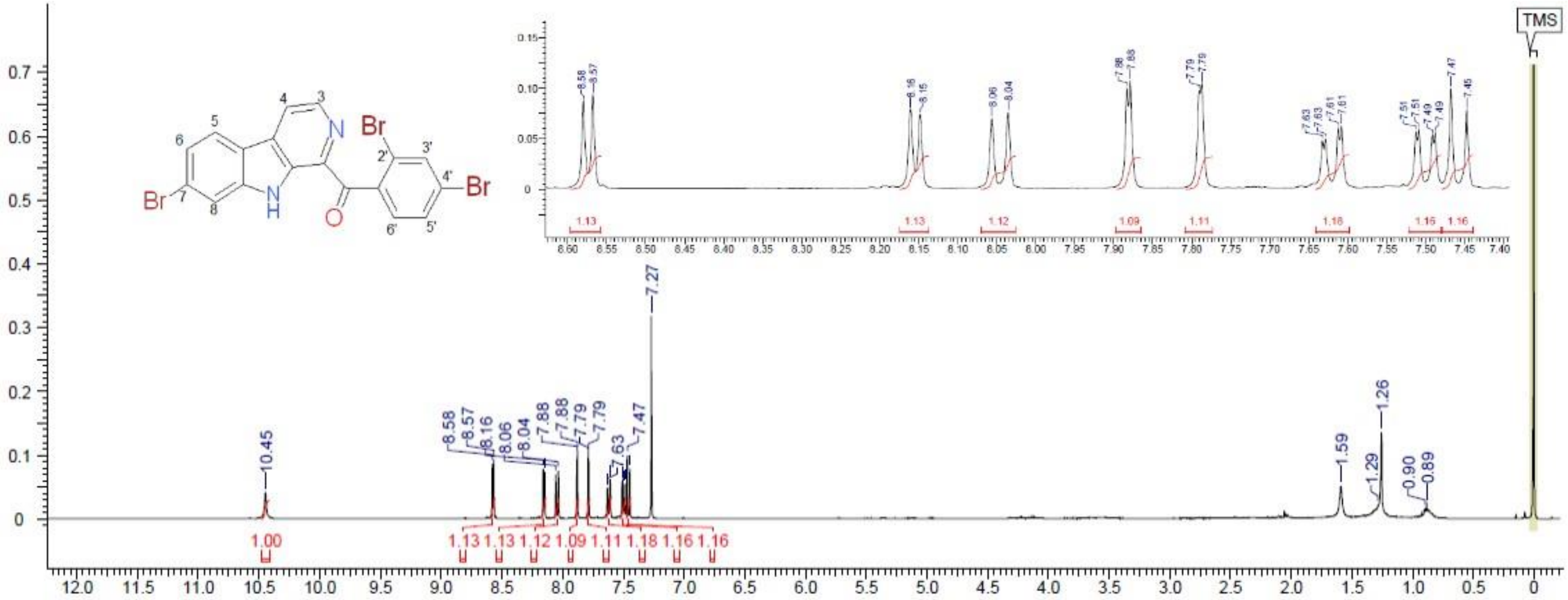

^{13}C NMR spectra of 1-(2’,4’-dibromobenzoyl)-7-bromo-β-carboline

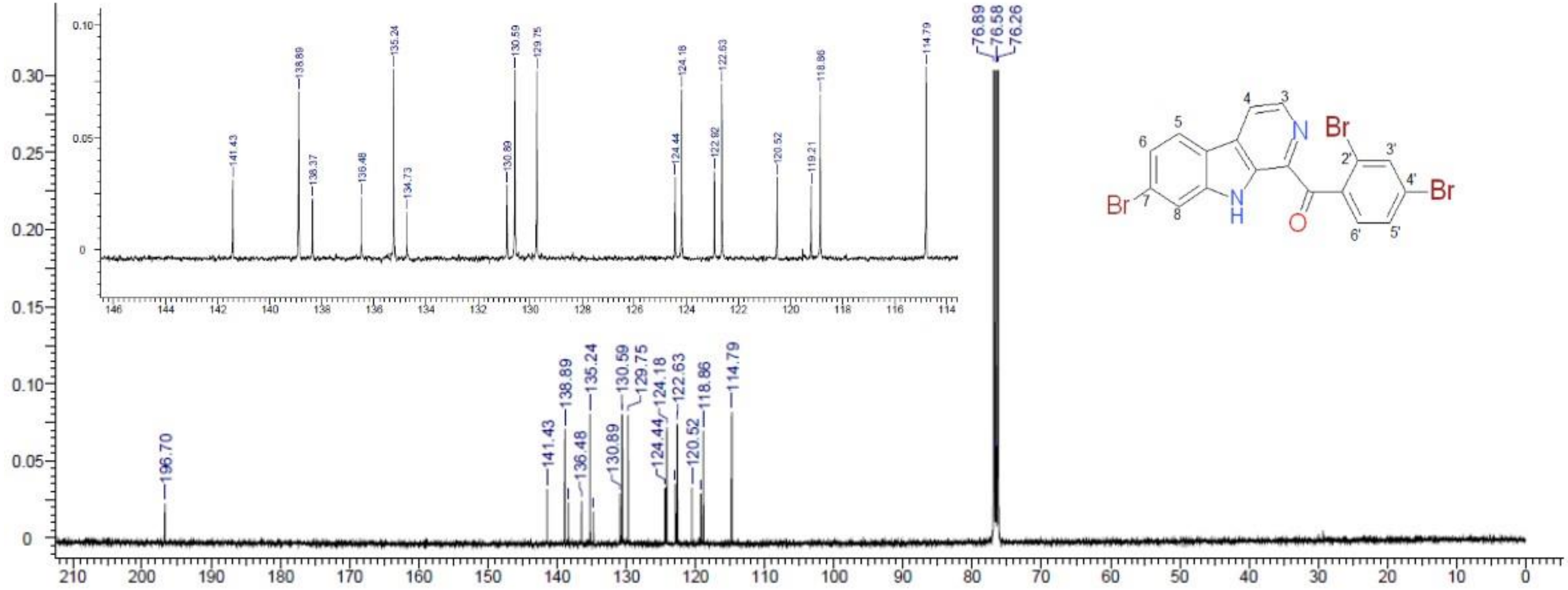

^{1}H NMR spectra of 1-(2’,4’-dibromobenzoyl)-5-bromo-β-carboline

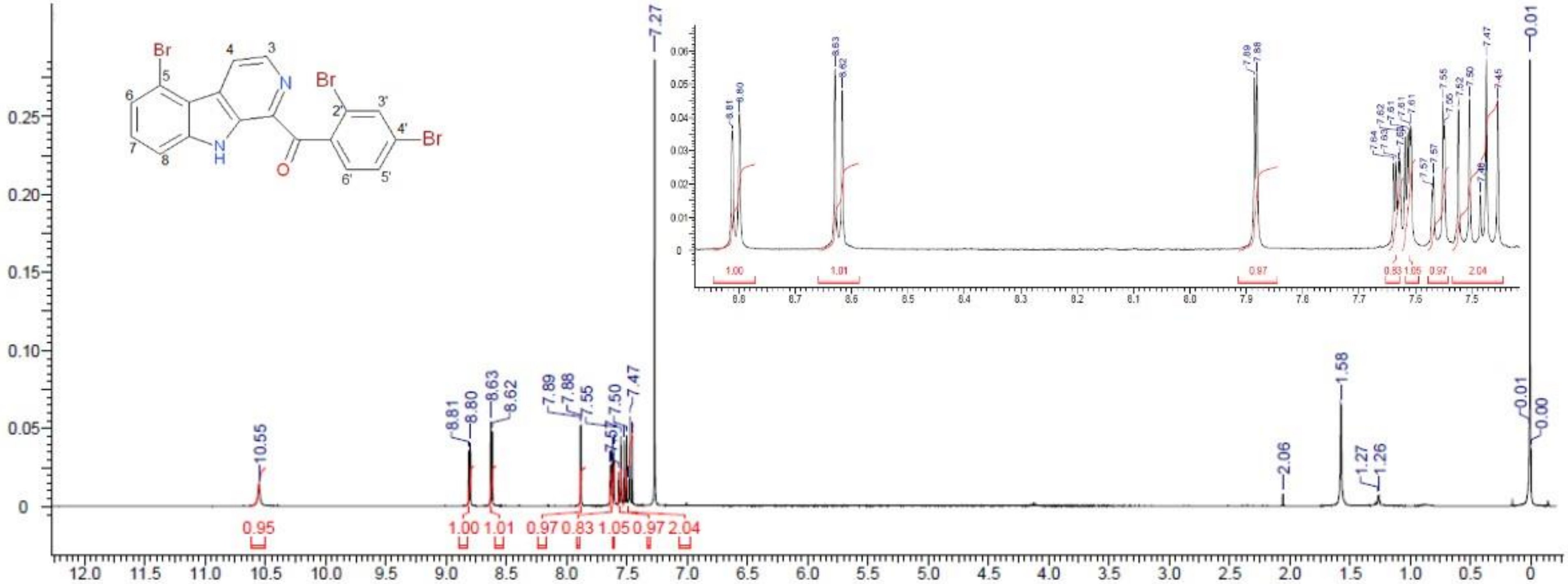

^{13}C NMR spectra of 1-(2’,4’-dibromobenzoyl)-5-bromo-β-carboline

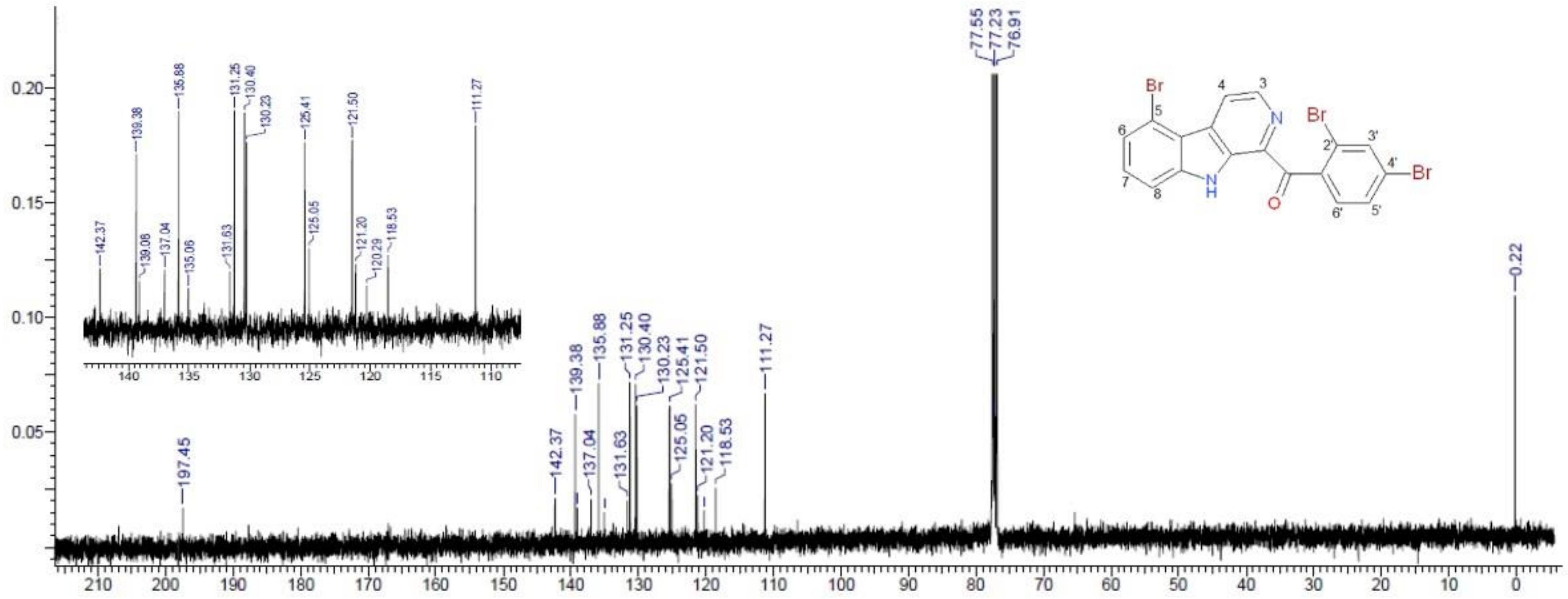

^{1}H NMR spectra of 1-(2'-bromobenzoyl)-3-methyl-β-carboline

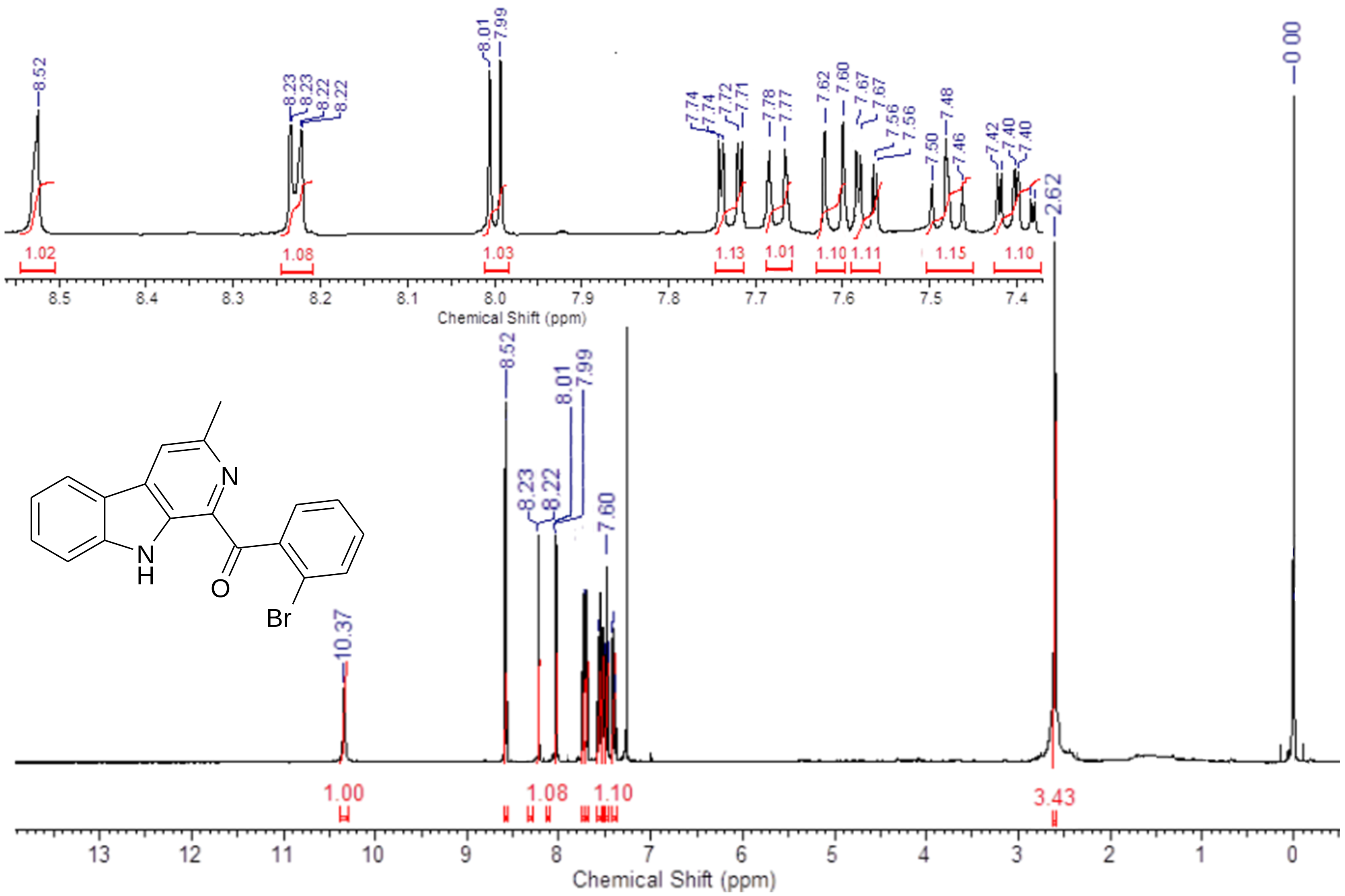

^{13}C NMR spectra of 1-(2'-bromobenzoyl)-3-methyl-β-carboline

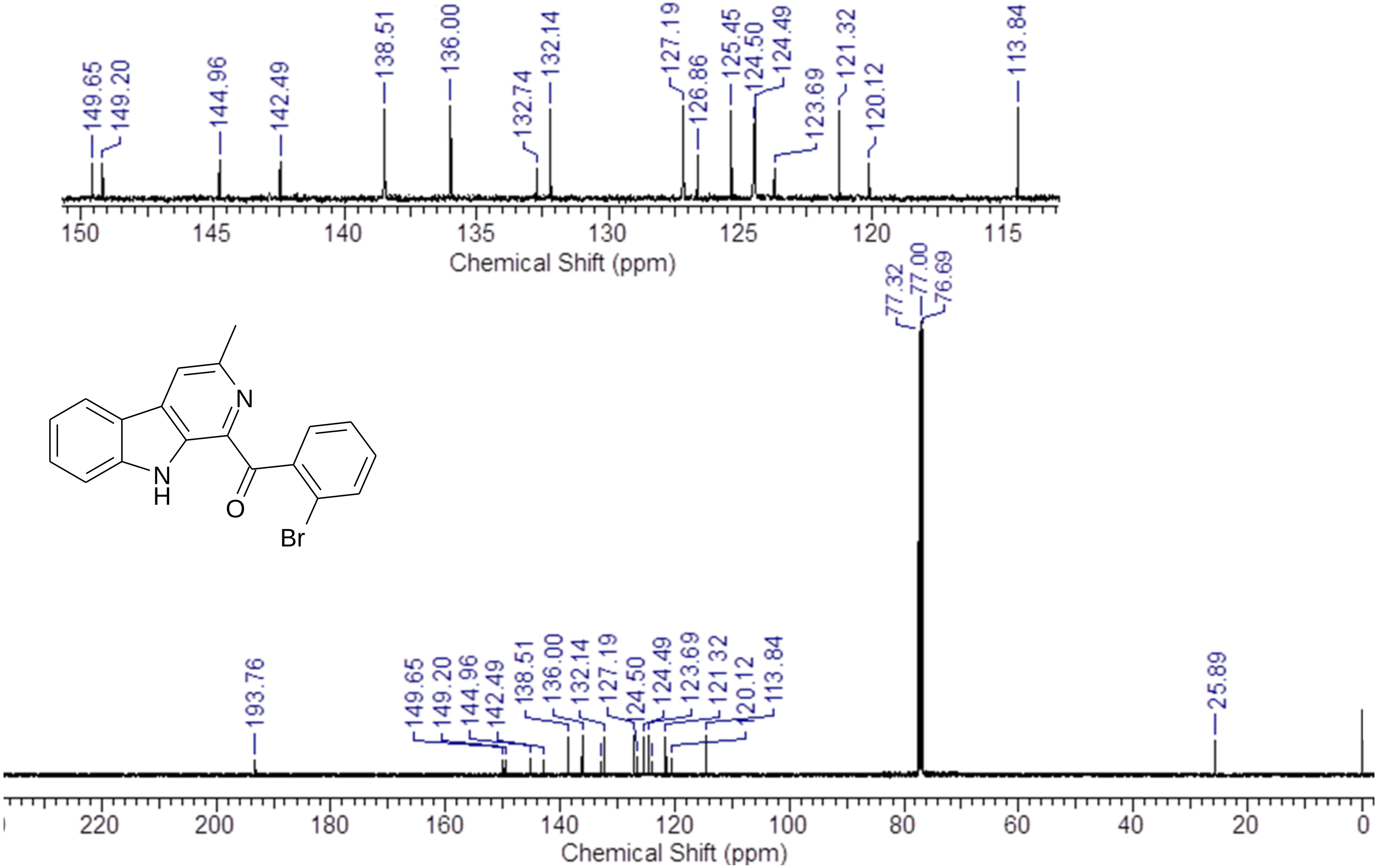

^{1}H NMR spectra of 1-(2'-bromobenzoyl)-3-phenyl-β-carboline

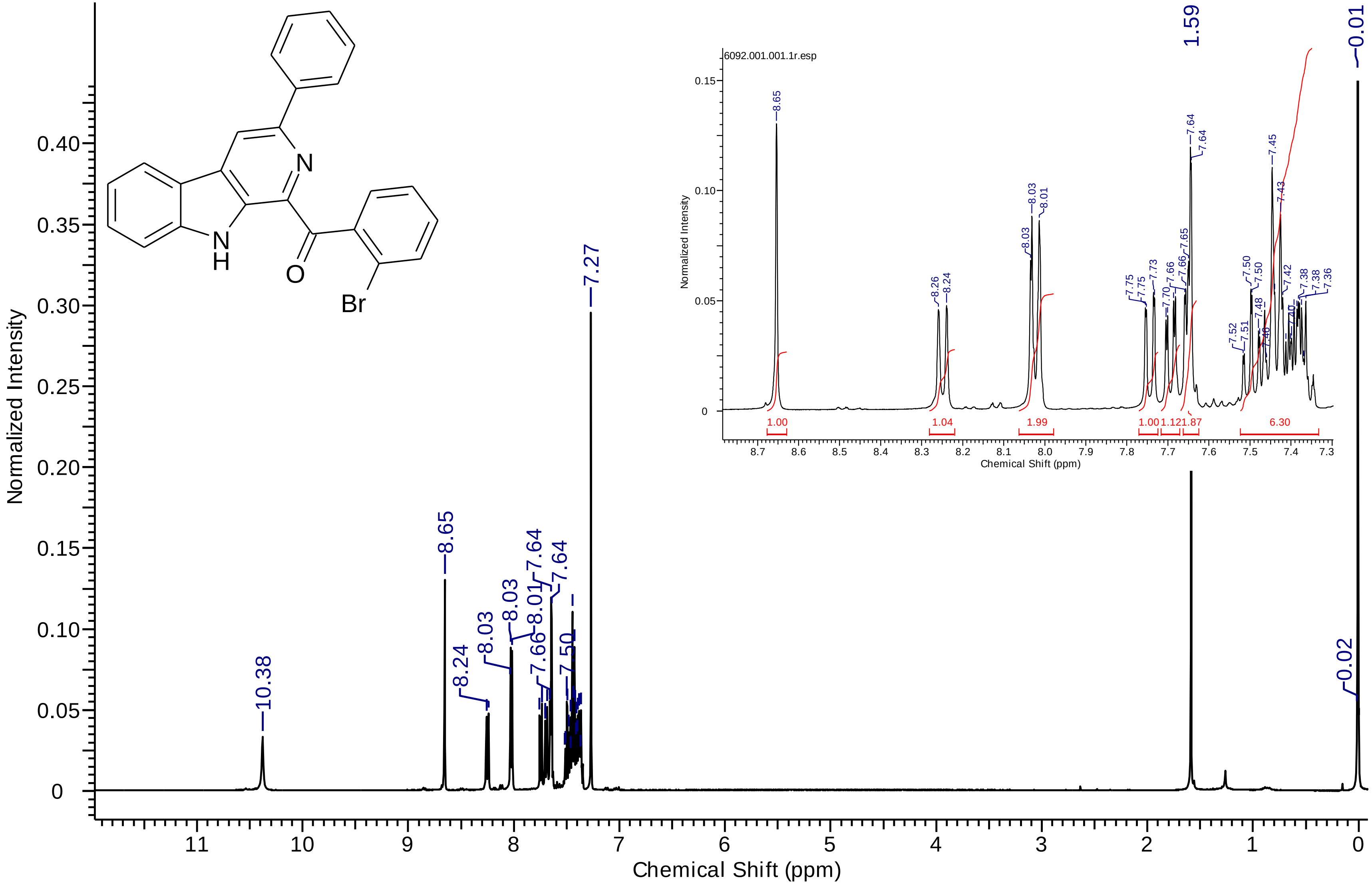

^{13}C NMR spectra of 1-(2’-bromobenzoyl)-3-phenyl-β-carboline

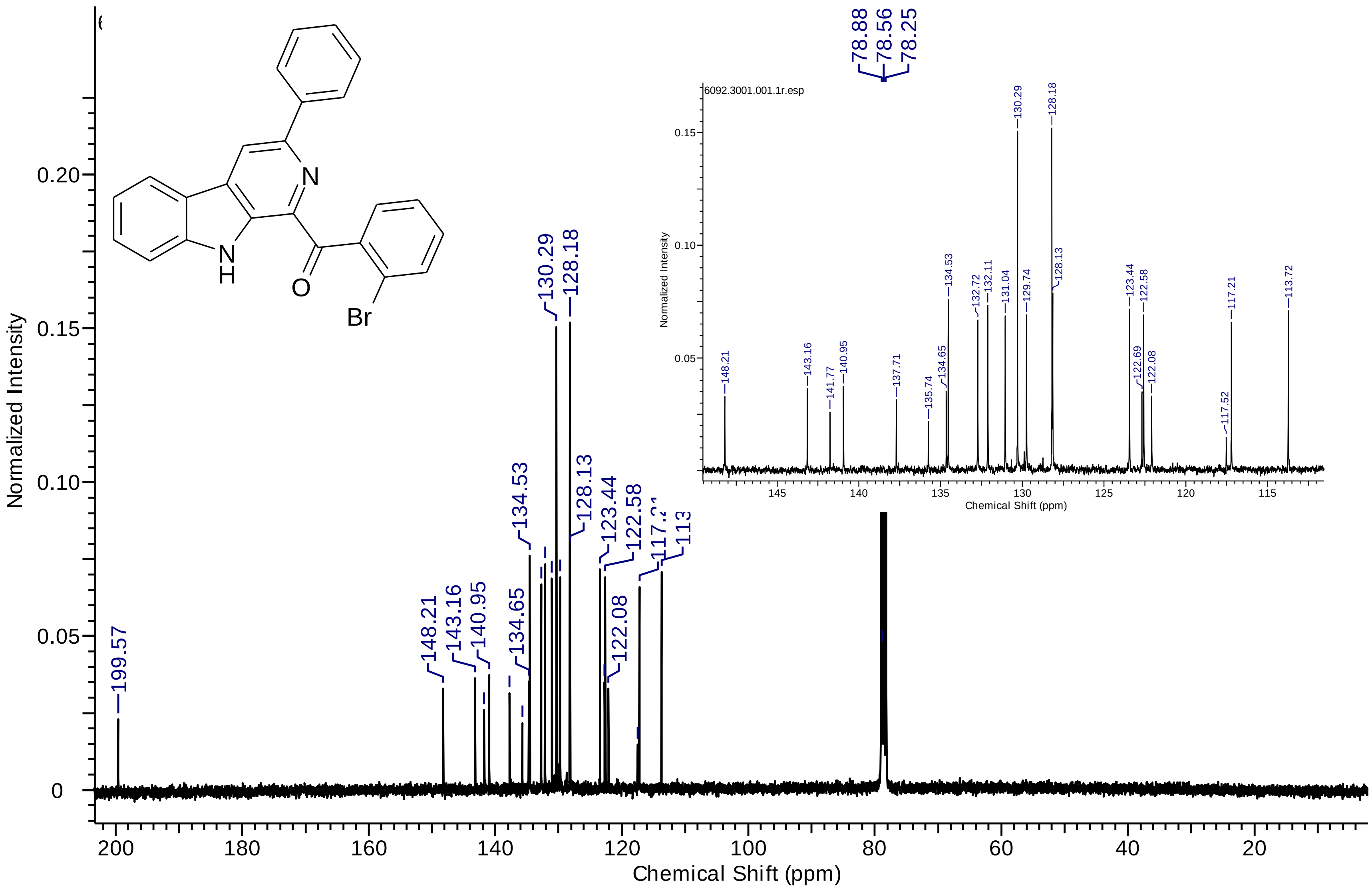

^{1}H NMR spectra of 1-(2’-bromobenzoyl)-6-bromo-β-carboline

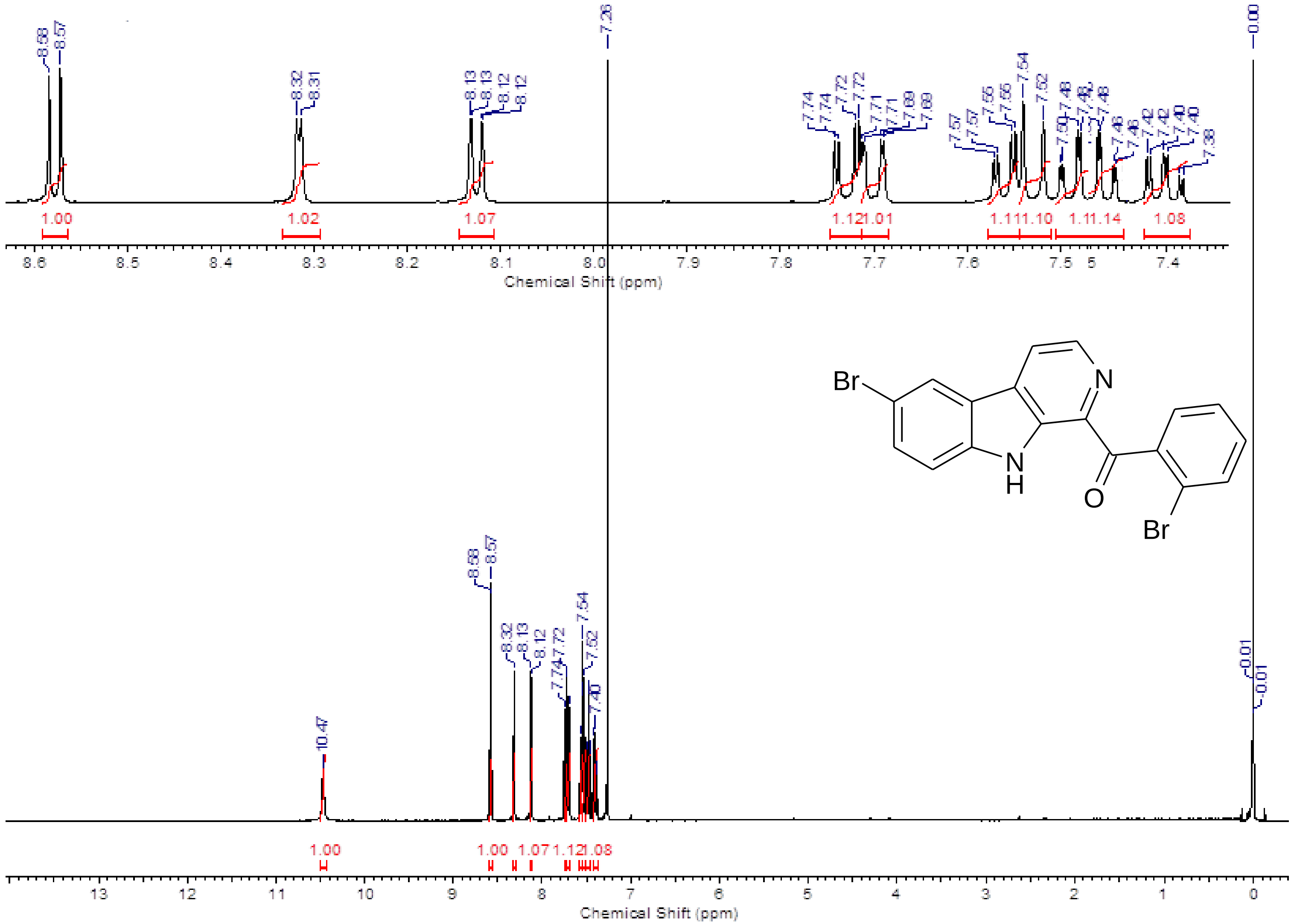

^{13}C NMR spectra of 1-(2'-bromobenzoyl)-6-bromo-β-carboline

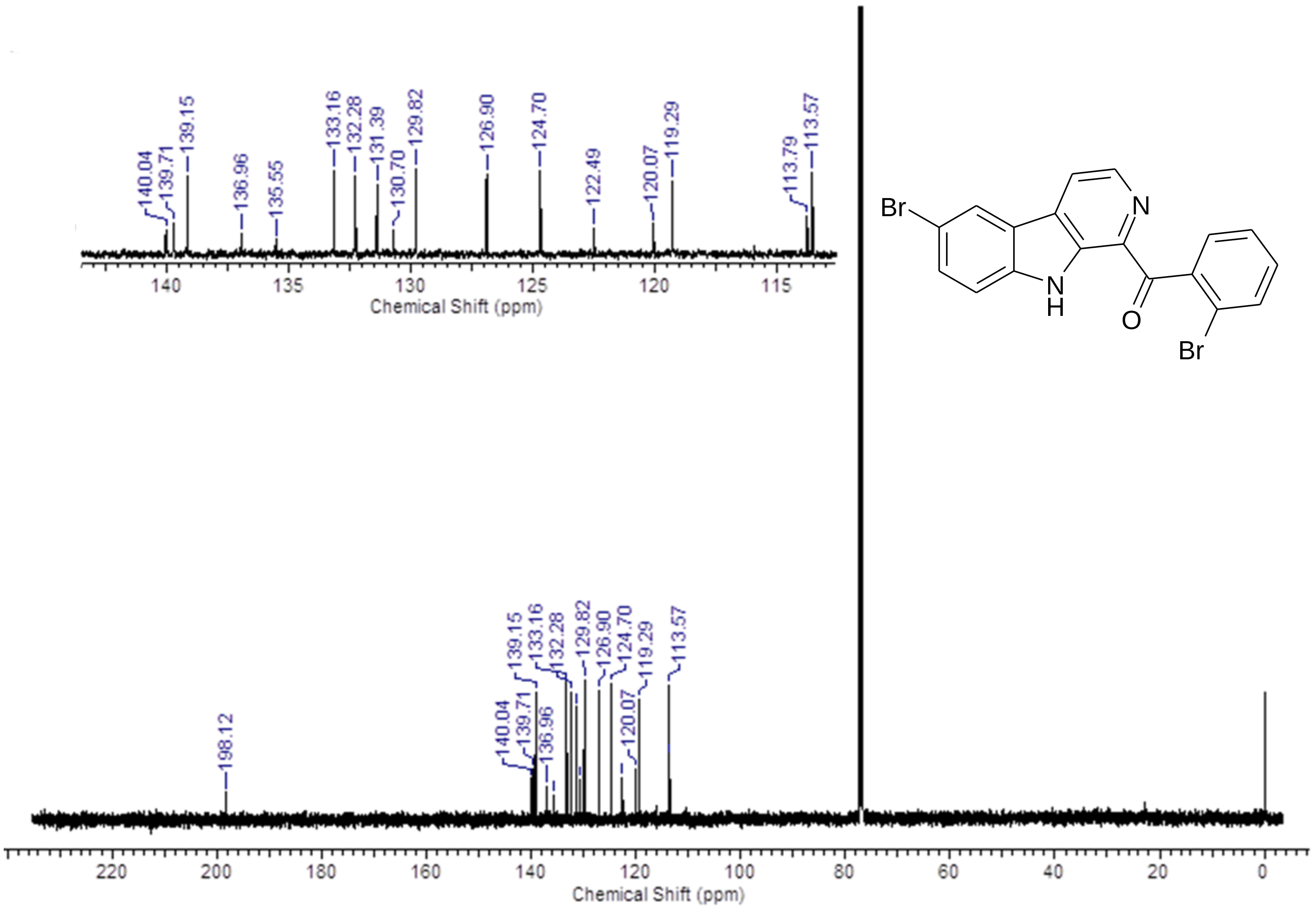

^{1}H NMR spectra of 1-(2’,5’-dibromobenzoyl)-6-bromo-β-carboline

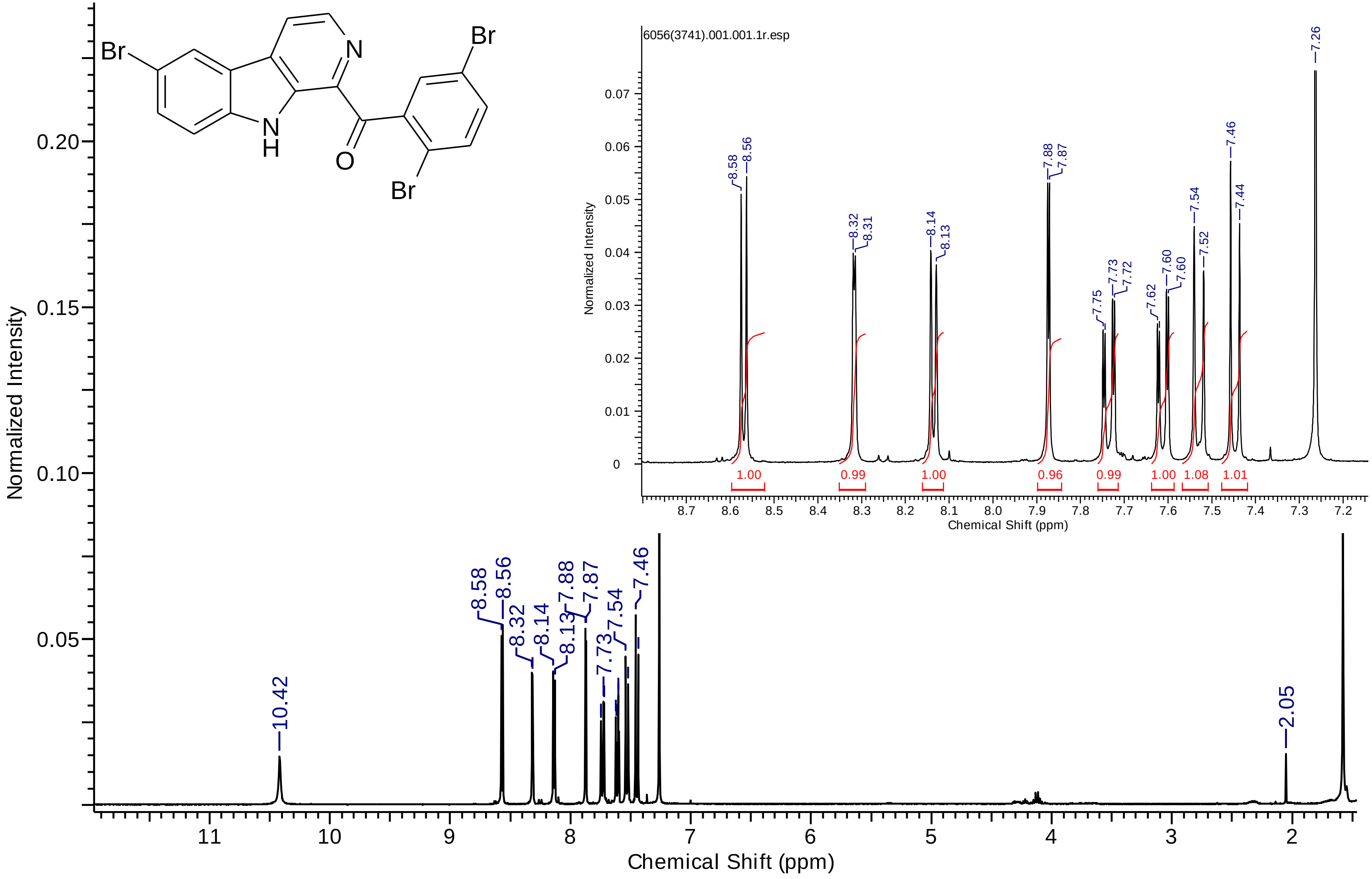

^{13}C NMR spectra of 1-(2’,5’-dibromobenzoyl)-6-bromo-β-carboline

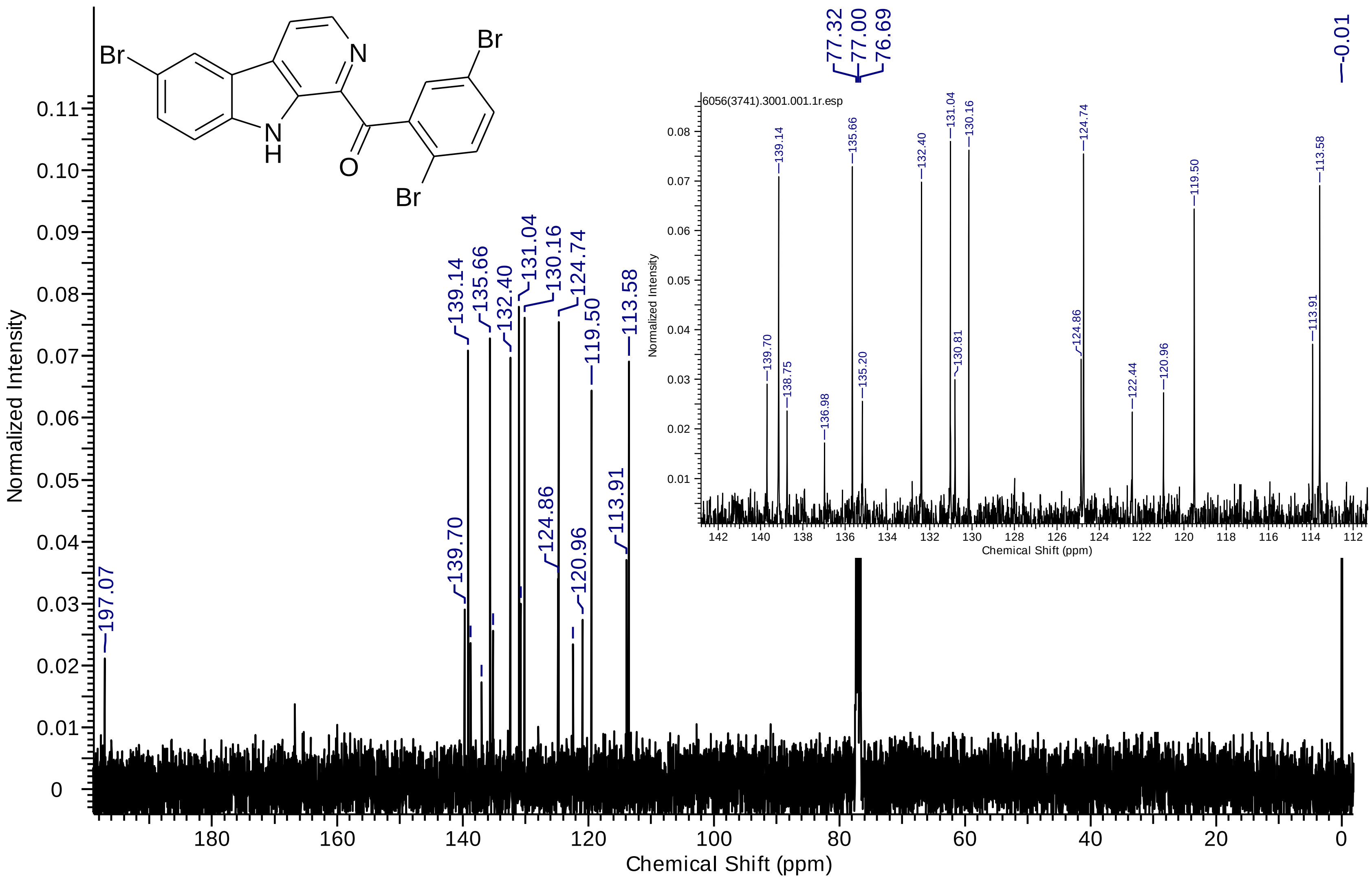

^{1}H NMR spectra of 1-(2’,4’-dibromobenzoyl)-6-bromo-β-carboline

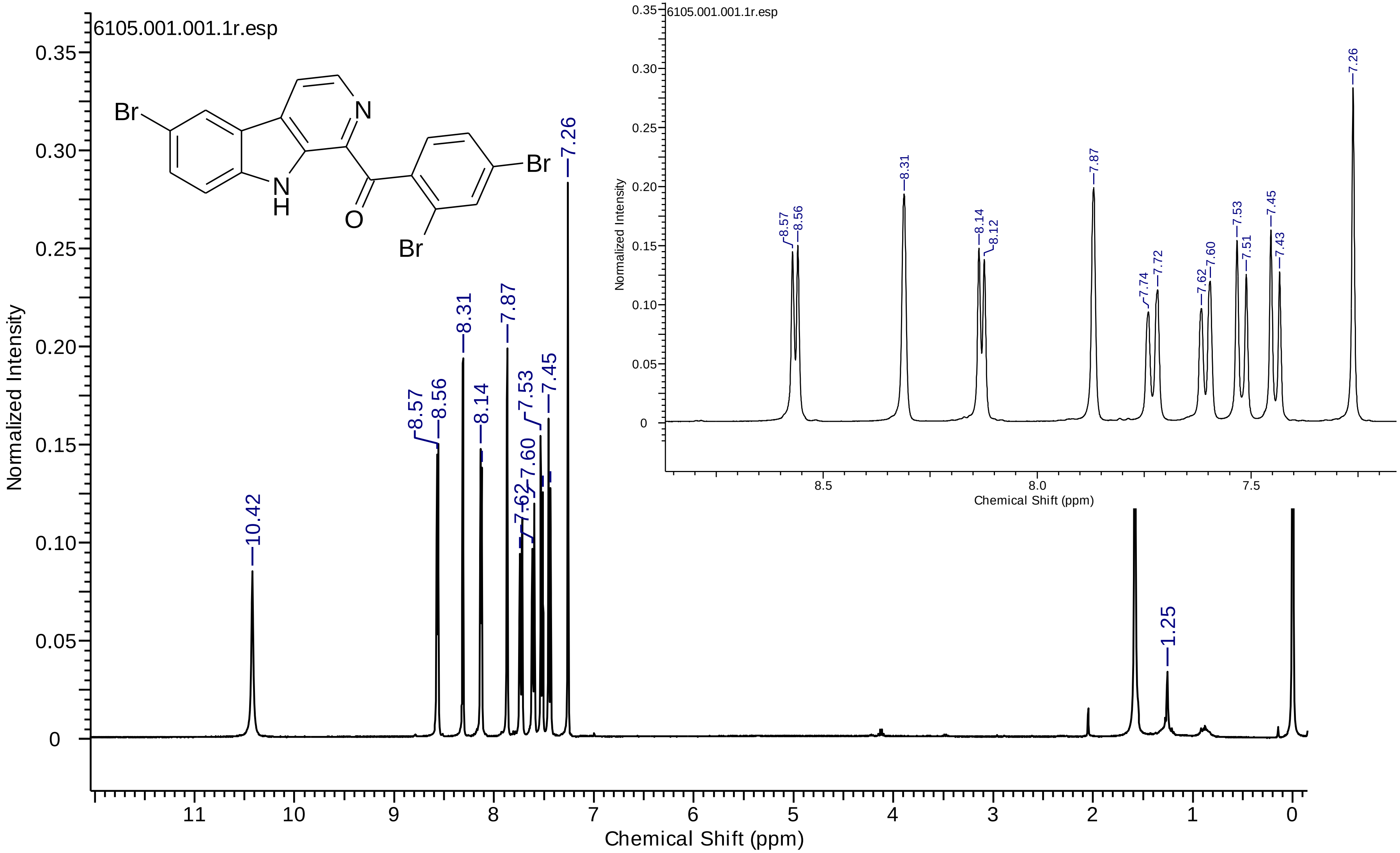

^{13}C NMR spectra of 1-(2',4'-dibromobenzoyl)-6-bromo-β-carboline

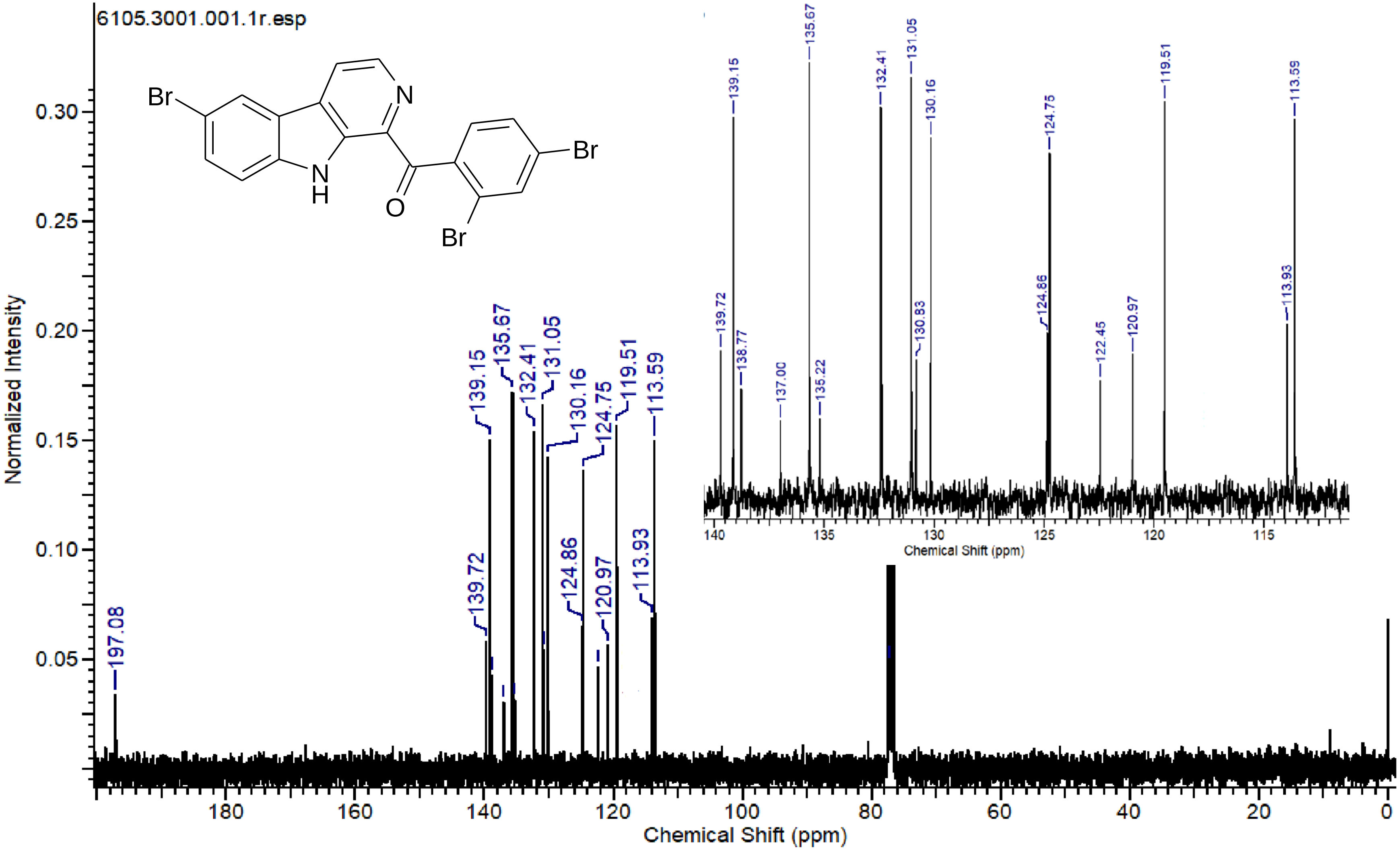

^{1}H NMR spectra of 1-(2’,5’-dibromobenzoyl)-6,8-dibromo-β-carboline

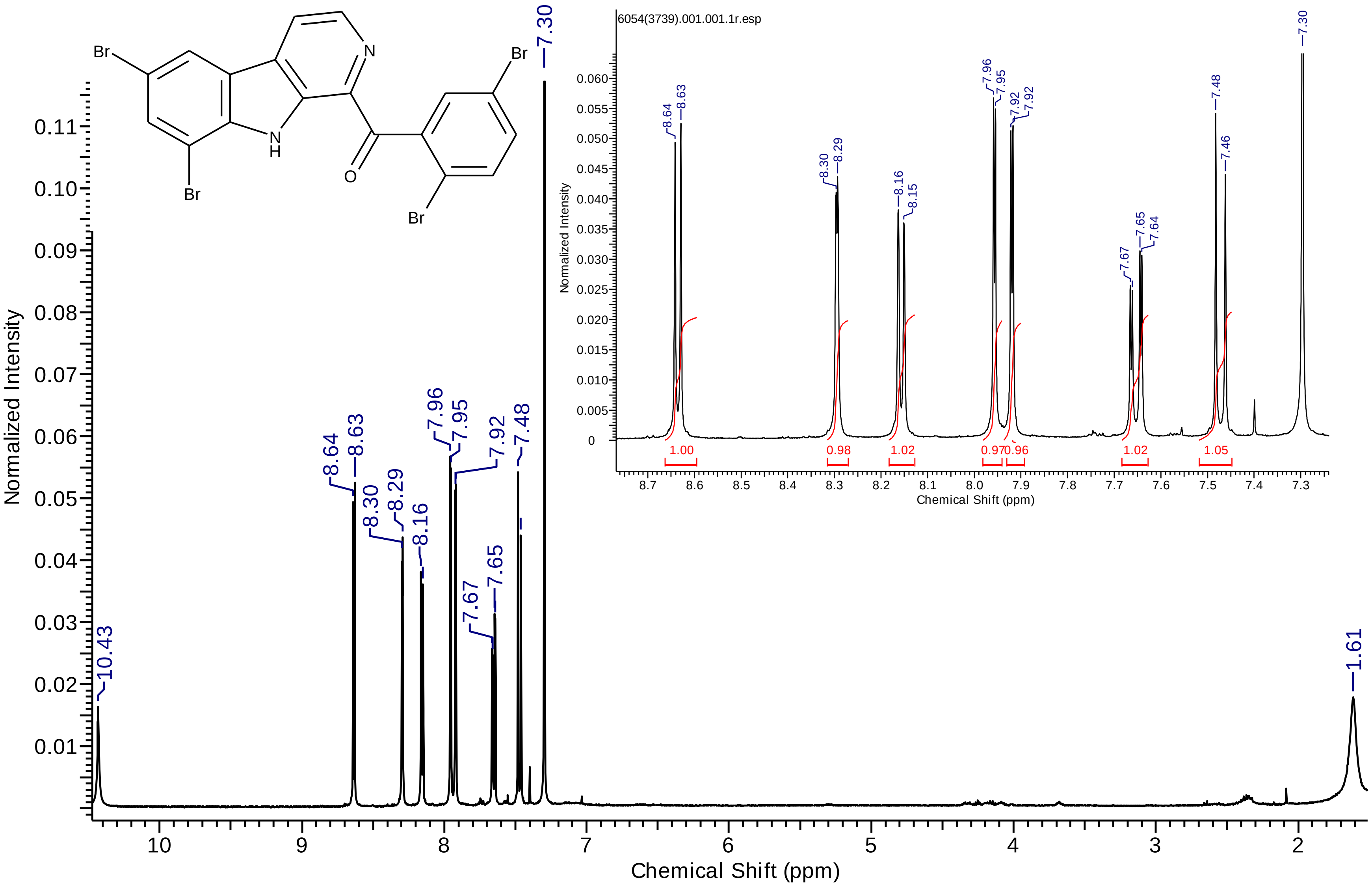

^{13}C NMR spectra of 1-(2',5'-dibromobenzoyl)-6,8-dibromo-β-carboline

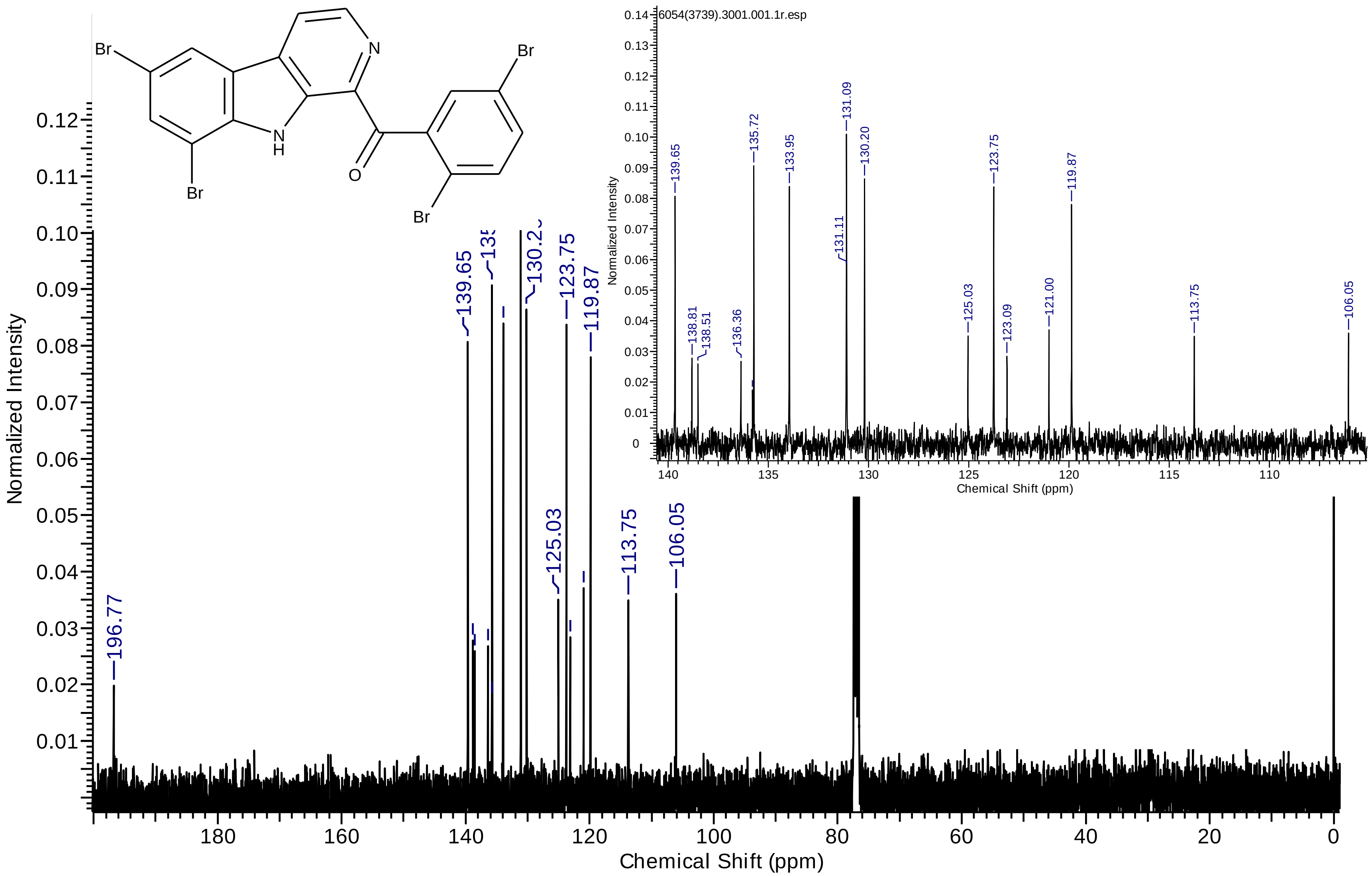

^{1}H NMR spectra of 1-(2'-bromobenzoyl)-6-iodo-β-carboline

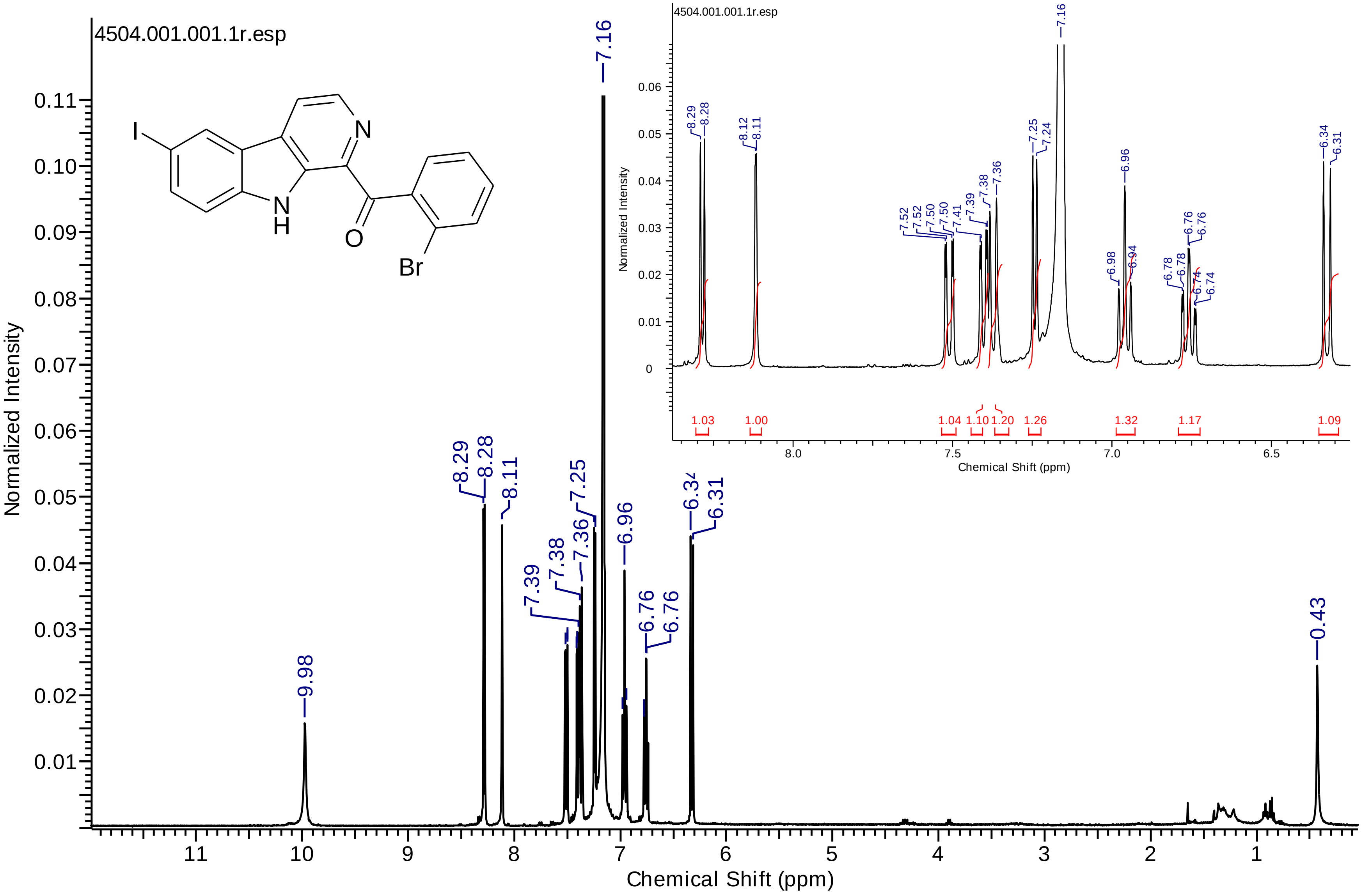

^{13}C NMR spectra of 1-(2'-bromobenzoyl)-6-iodo-β-carboline

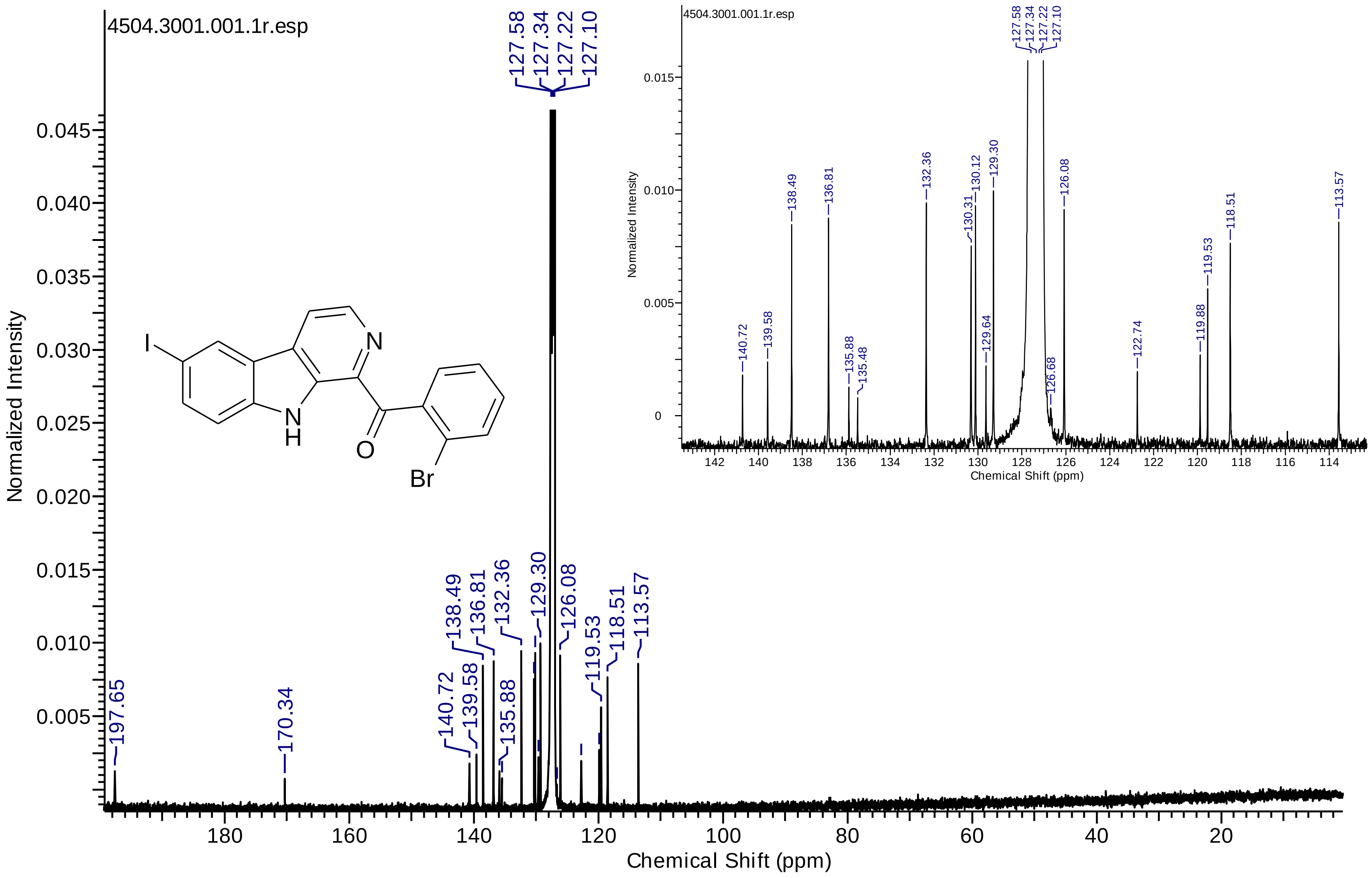

^{1}H NMR spectra of 1-(2',5'-dibromobenzoyl)-6-iodo-β-carboline

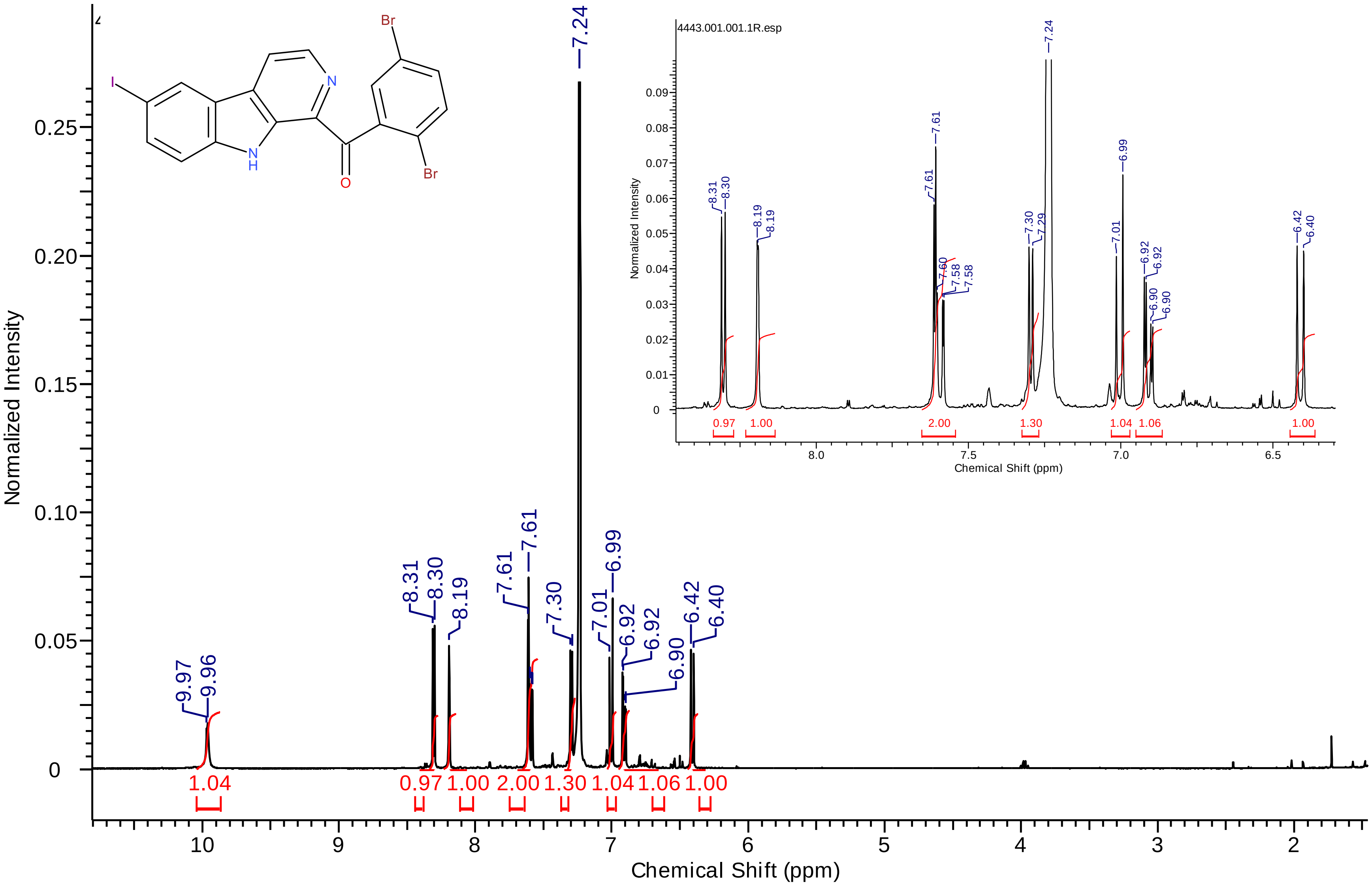

^{13}C NMR spectra of 1-(2',5'-dibromobenzoyl)-6-iodo-β-carboline

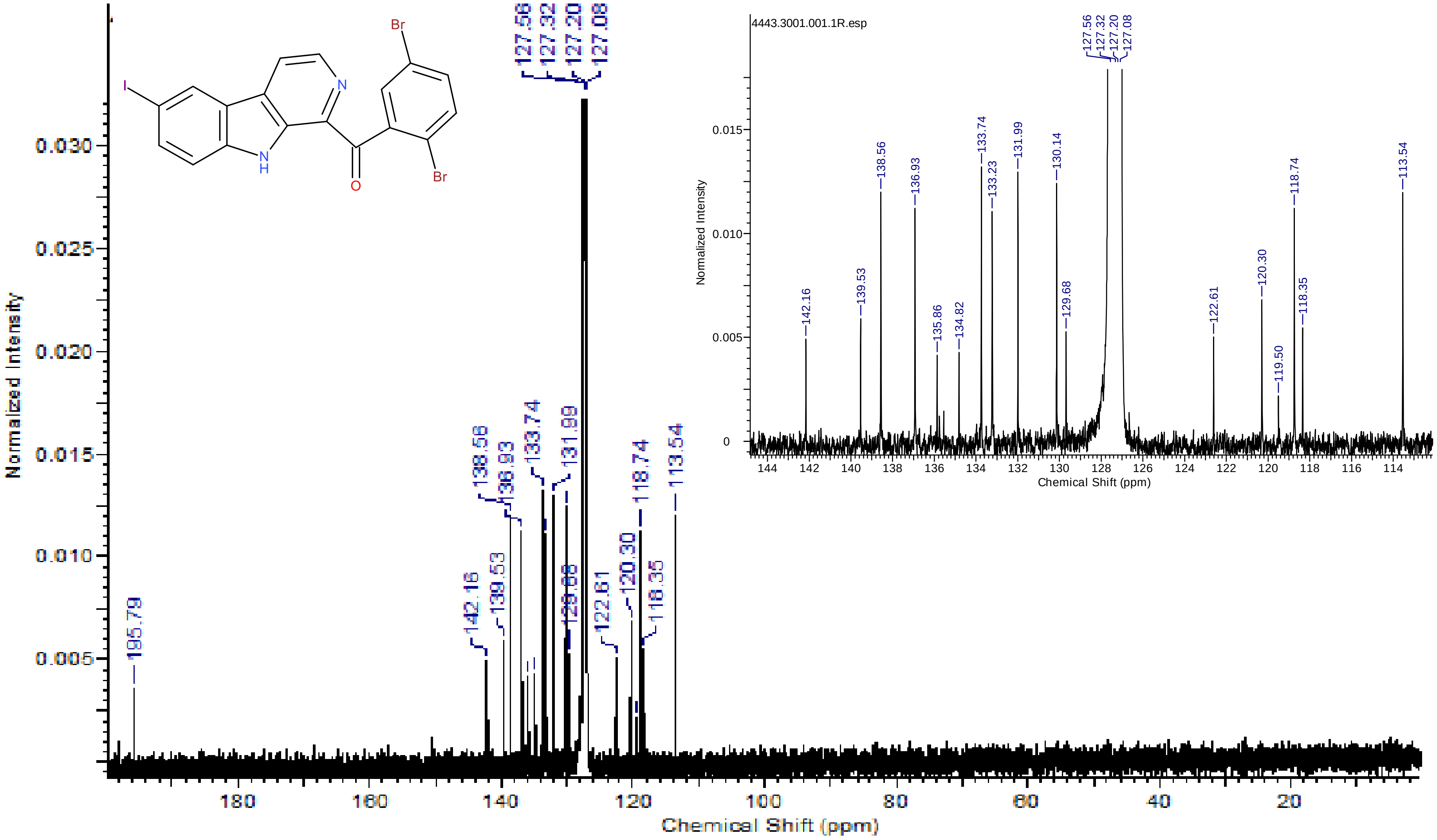

^{1}H NMR spectra of 1-(2',5'-dibromobenzoyl)-6-chloro-β-carboline

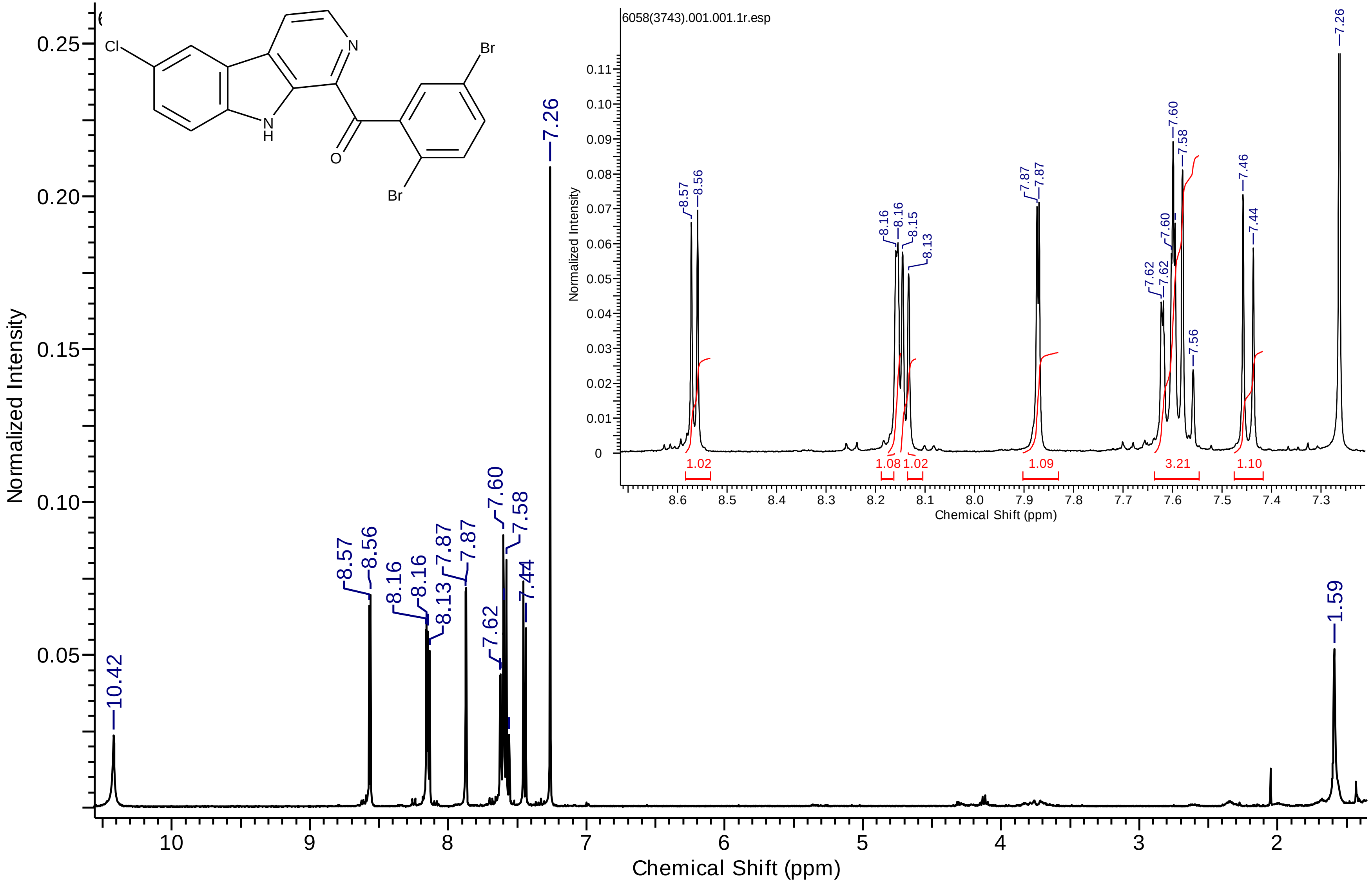

^{13}C NMR spectra of 1-(2’,5’-dibromobenzoyl)-6-chloro-β-carboline

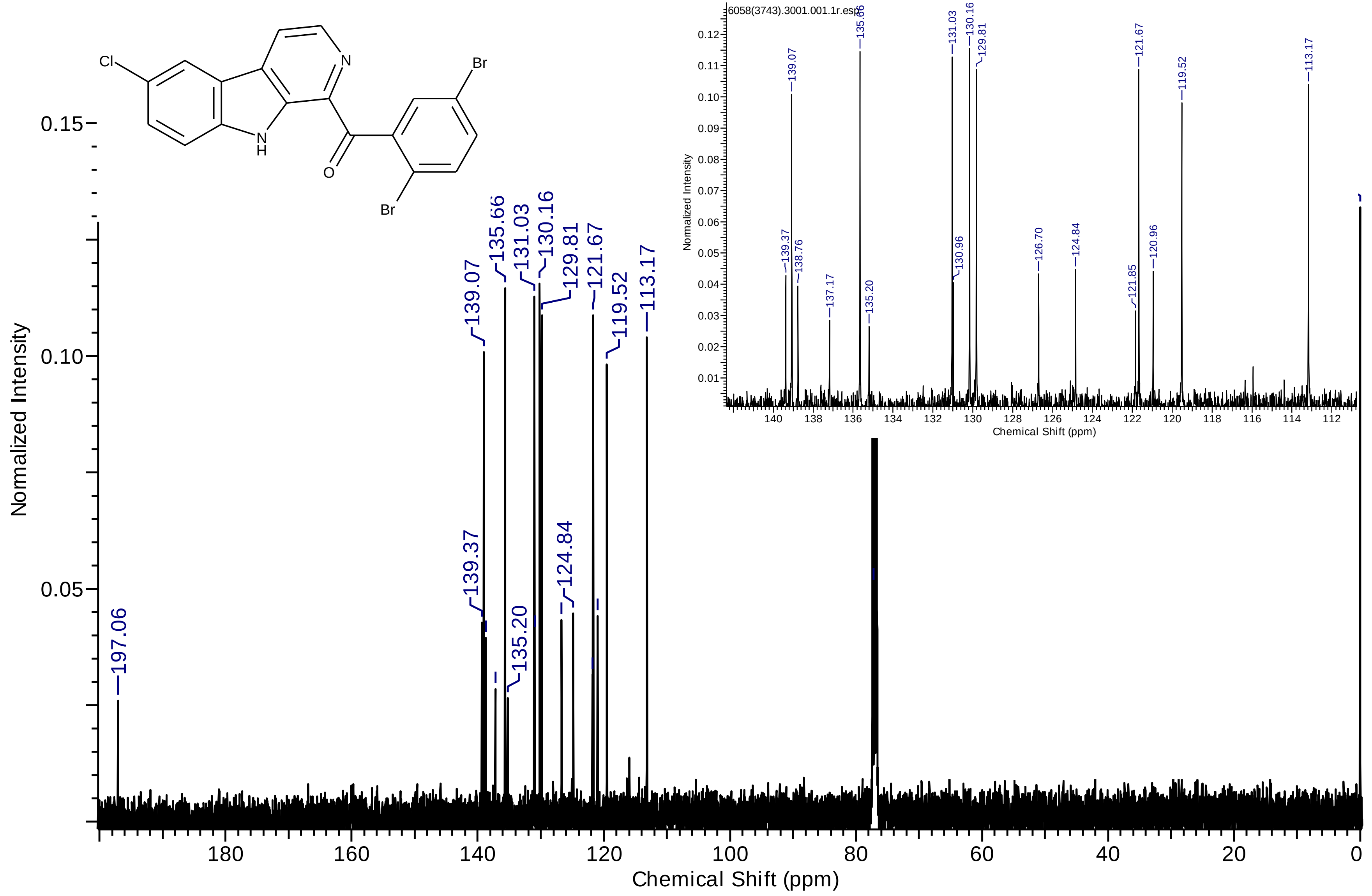

^{1}H NMR spectra of 1-(2',5'-dibromobenzoyl)-6.8-dichloro-β-carboline

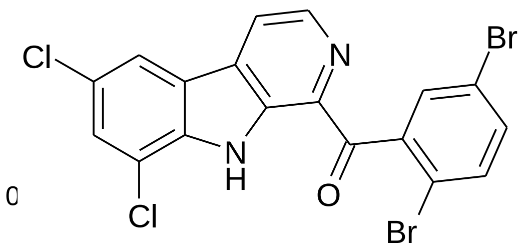

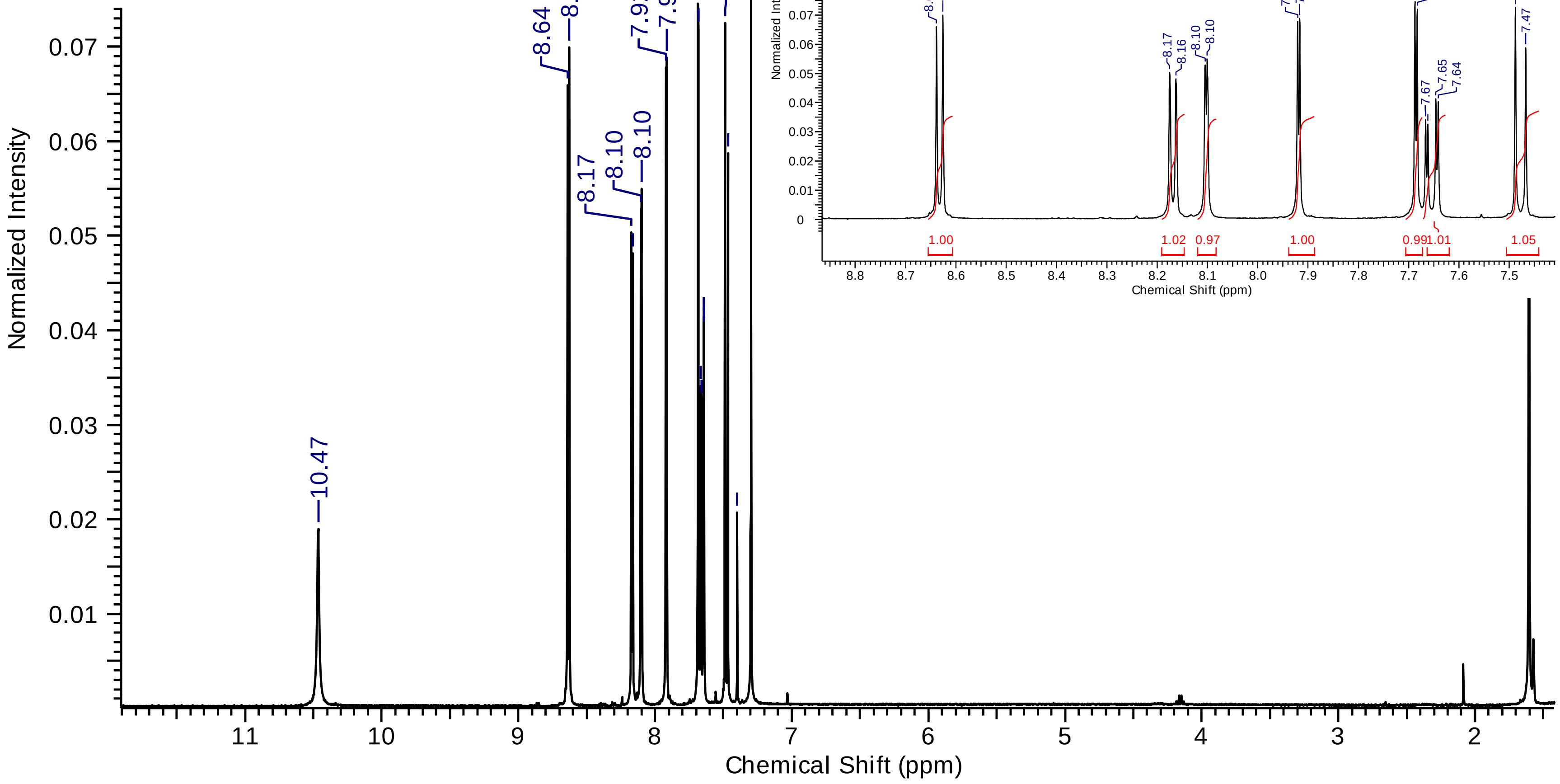

^{13}C NMR spectra of 1-(2'.5'-dibromobenzoyl)-6.8-dichloro-β-carboline

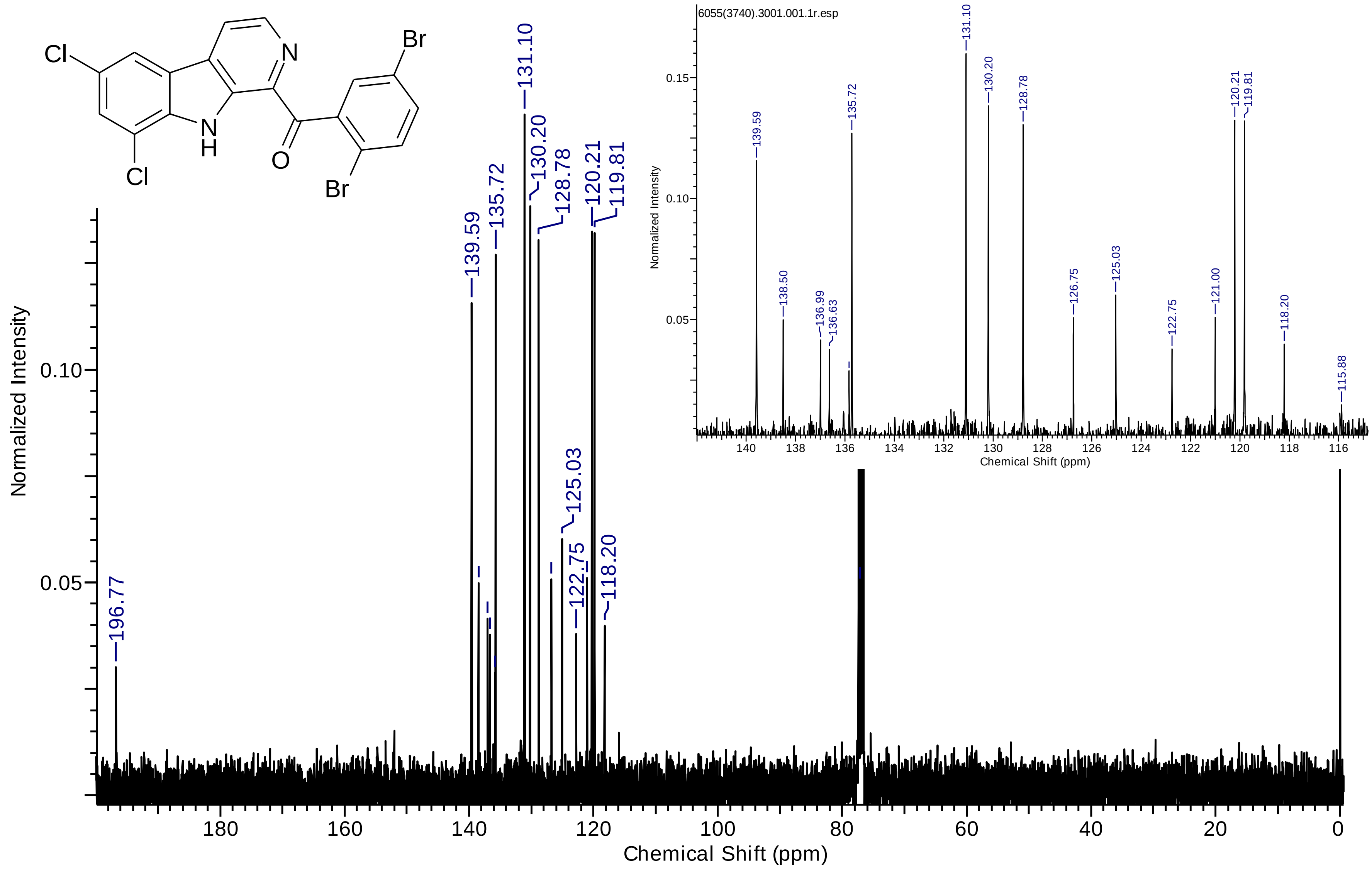

^{1}H NMR spectra of 3-bromofascaplysin

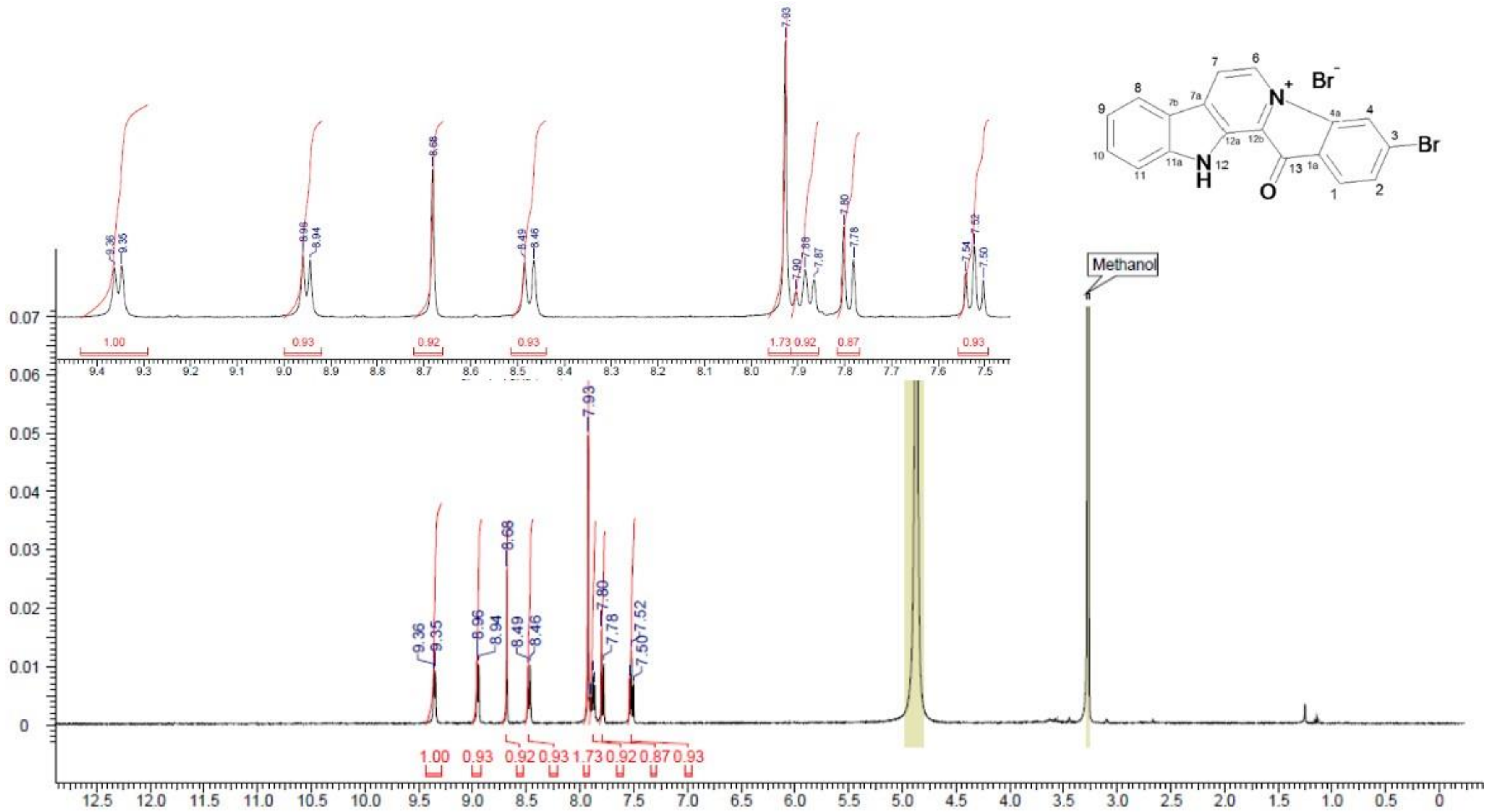

^{13}C NMR spectra of 3-bromofascaplysin

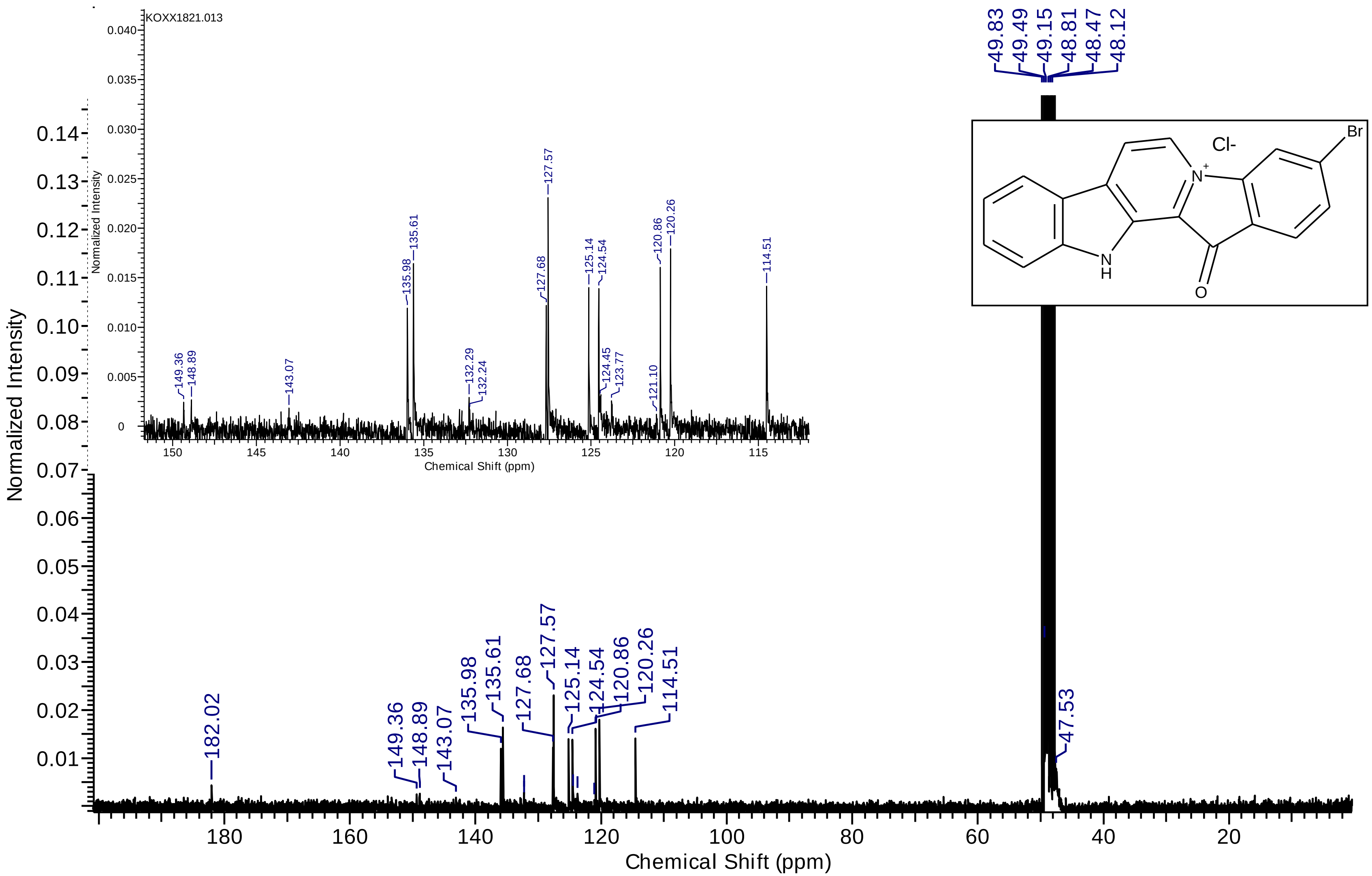

^{1}H NMR spectra of 12,13-dihydro-2-bromo-13-oxopyrido[1,2-*a*:3,4-*b'*]diindol-5-ium chloride

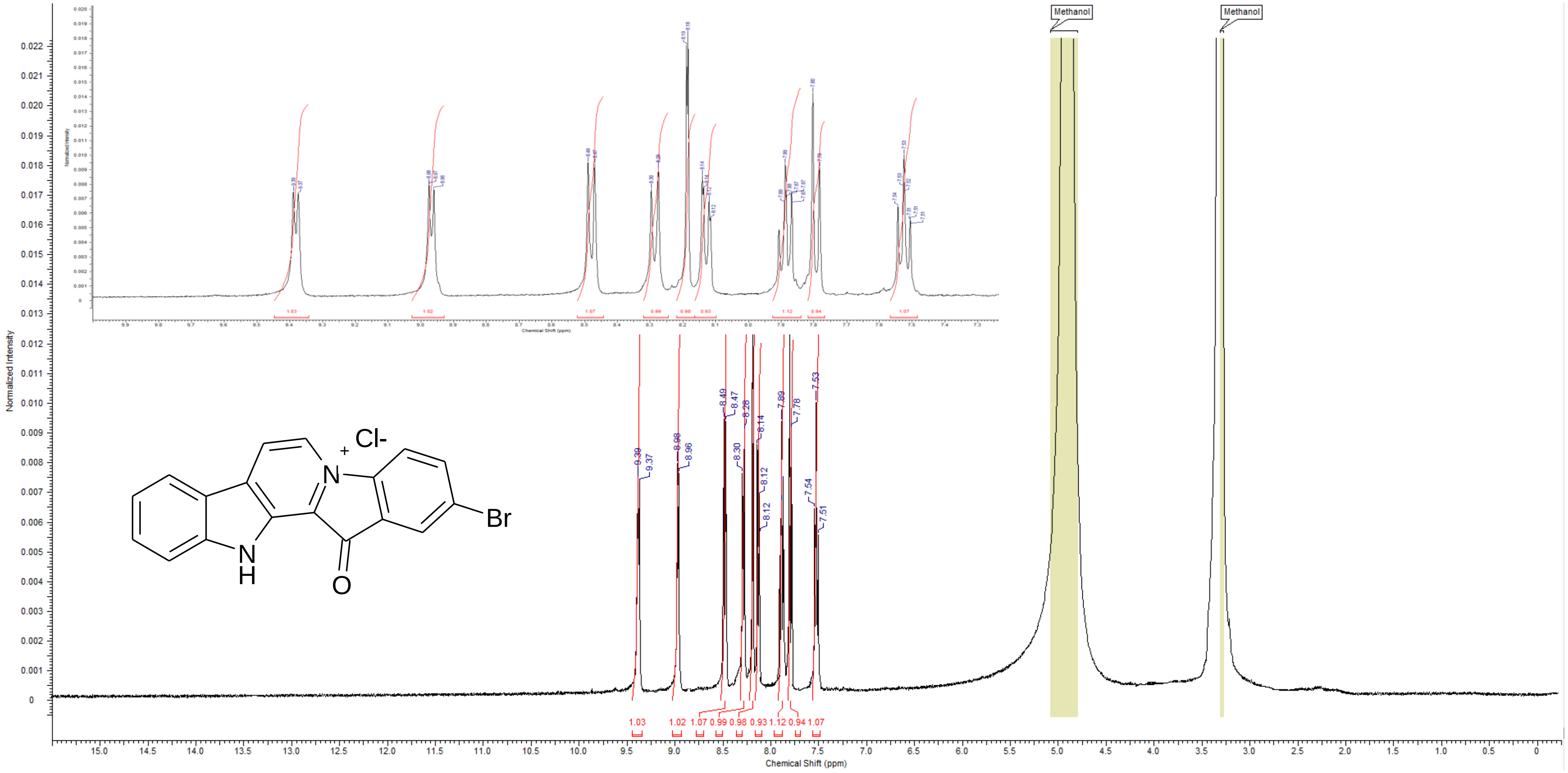

^{13}C NMR spectra of 12,13-dihydro-2-bromo-13-oxopyrido[1,2-*a*:3,4-*b'*]diindol-5-ium chloride

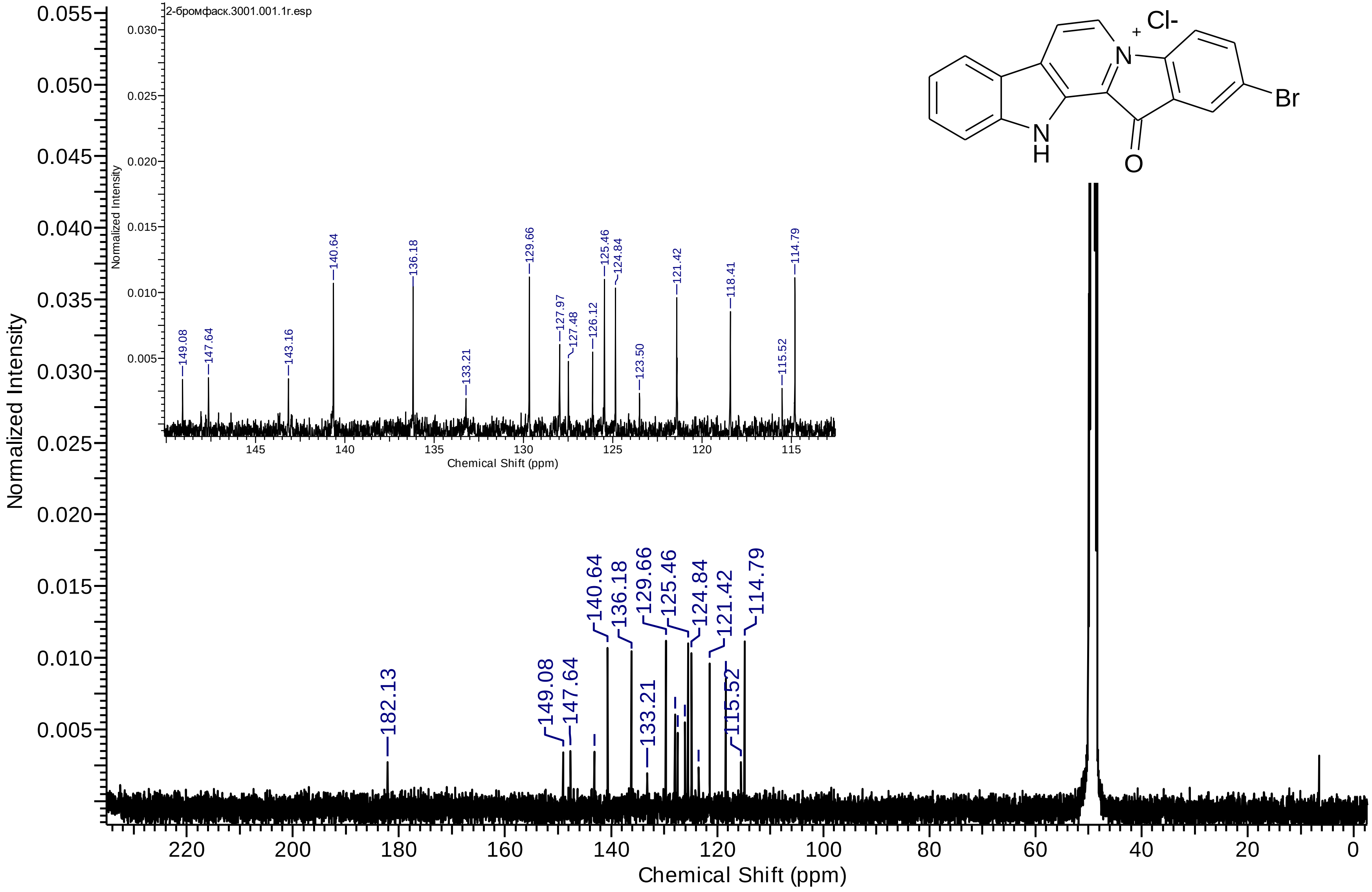

^{1}H NMR spectra of 12,13-dihydro-9-bromo-13-oxopyrido[1,2-*a*:3,4-*b'*]diindol-5-ium chloride

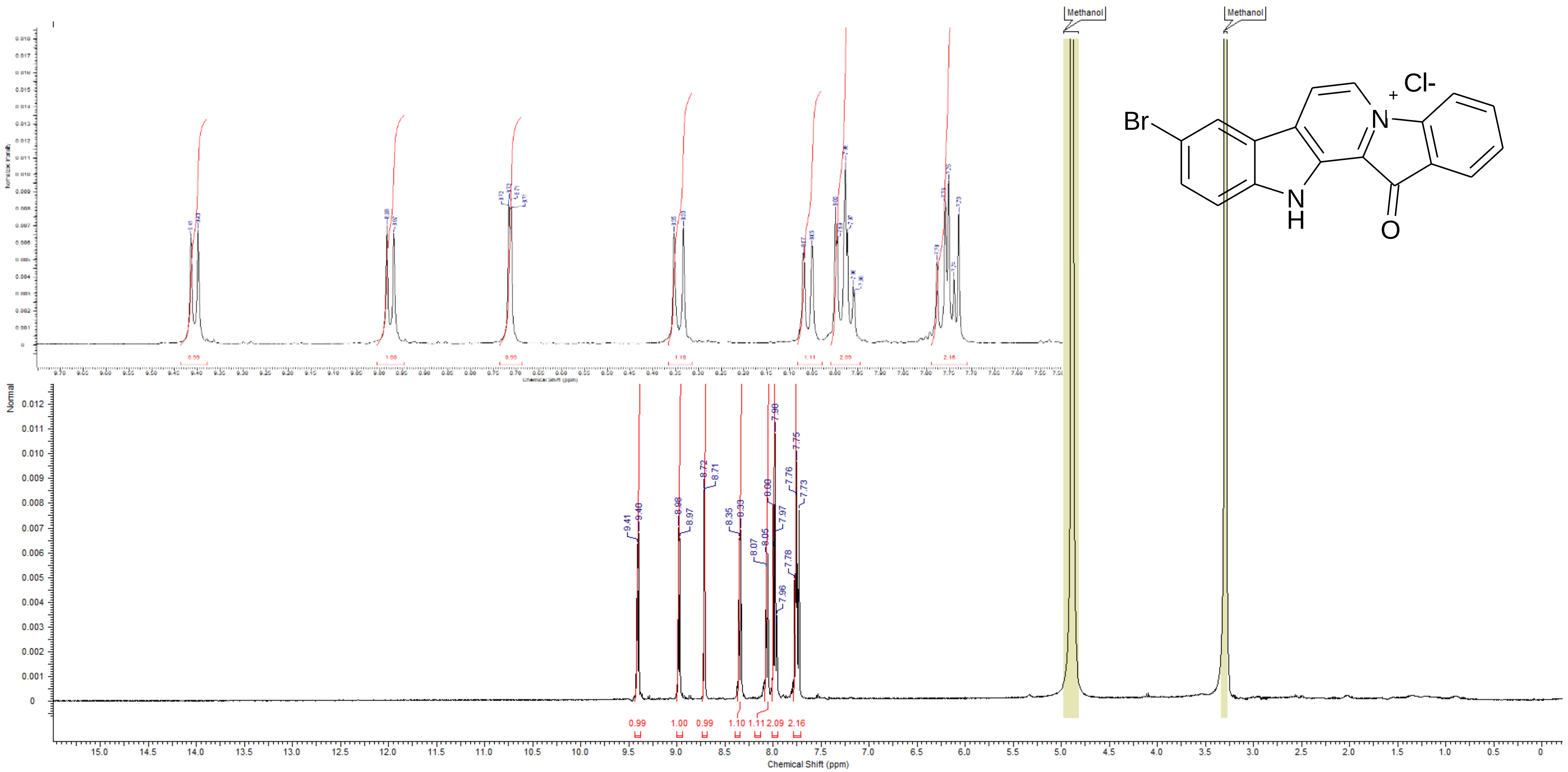

^{13}C NMR spectra of 12,13-dihydro-9-bromo-13-oxopyrido[1,2-*a*:3,4-*b'*]diindol-5-ium chloride

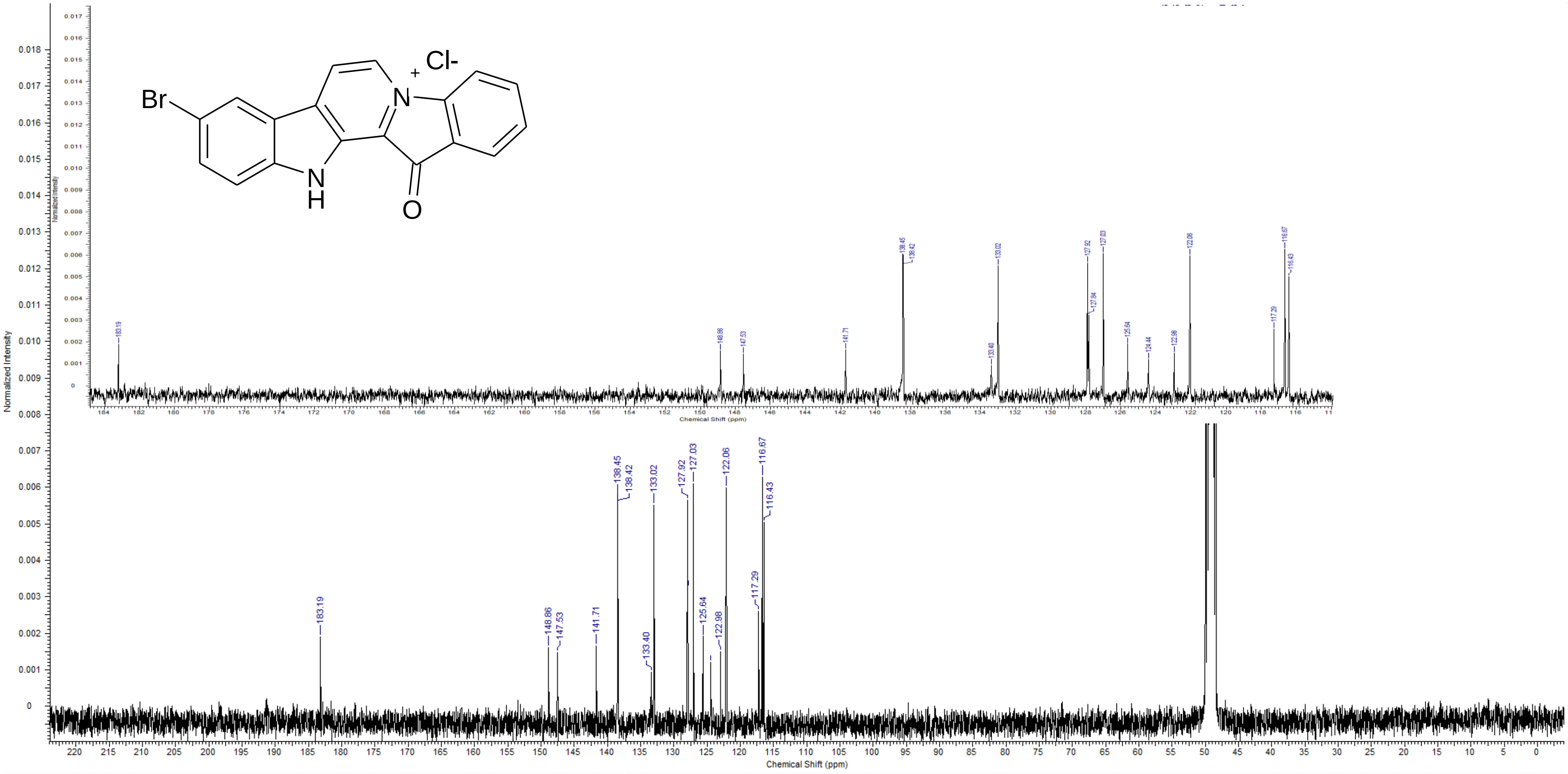

^{1}H NMR spectra of 12,13-dihydro-9-iodo-13-oxopyrido[1,2-*a*:3,4-*b'*]diindol-5-ium chloride

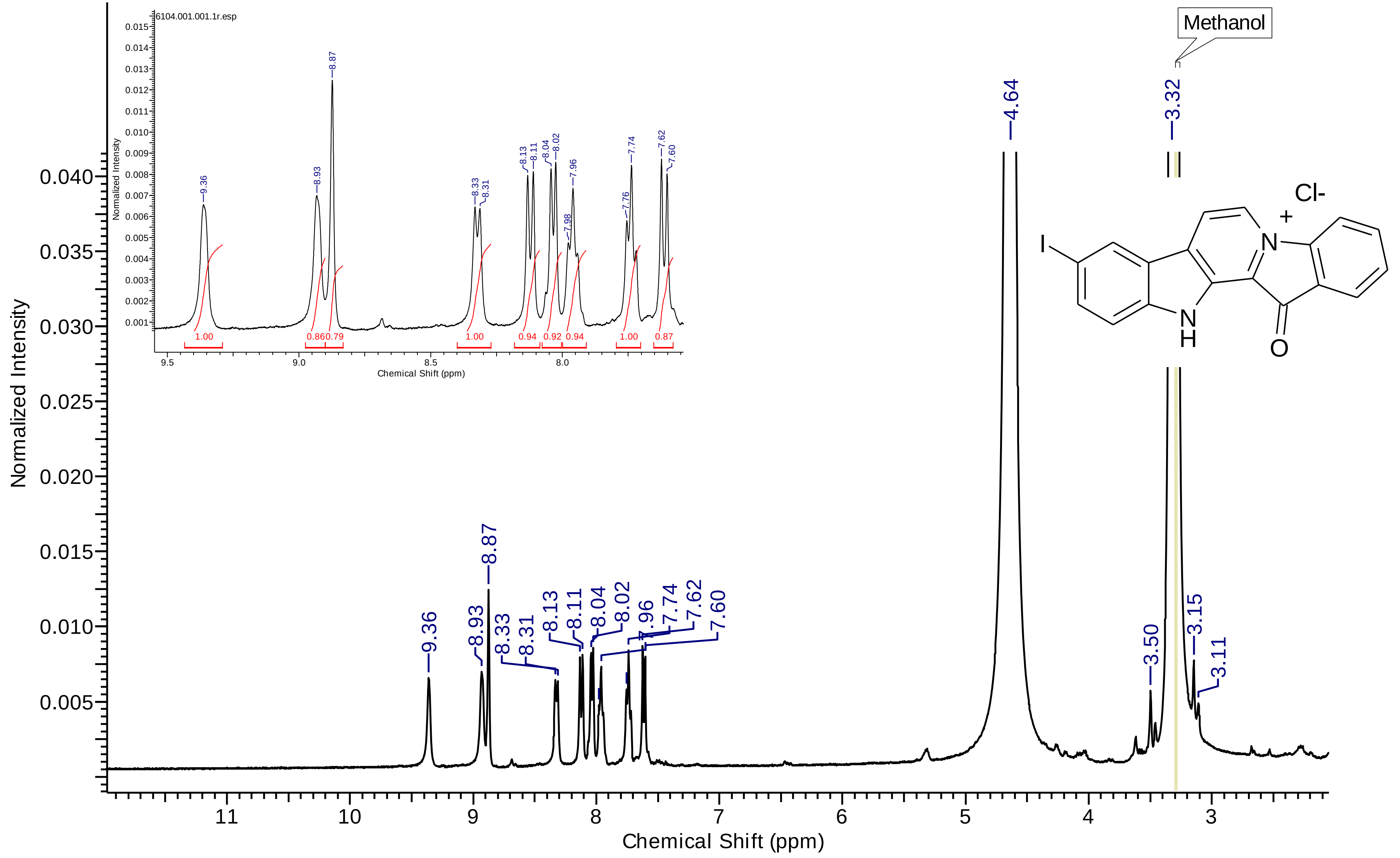

^{13}C NMR spectra of of 12,13-dihydro-9-iodo-13-oxopyrido[1,2-*a*:3,4-*b'*]diindol-5-ium chloride

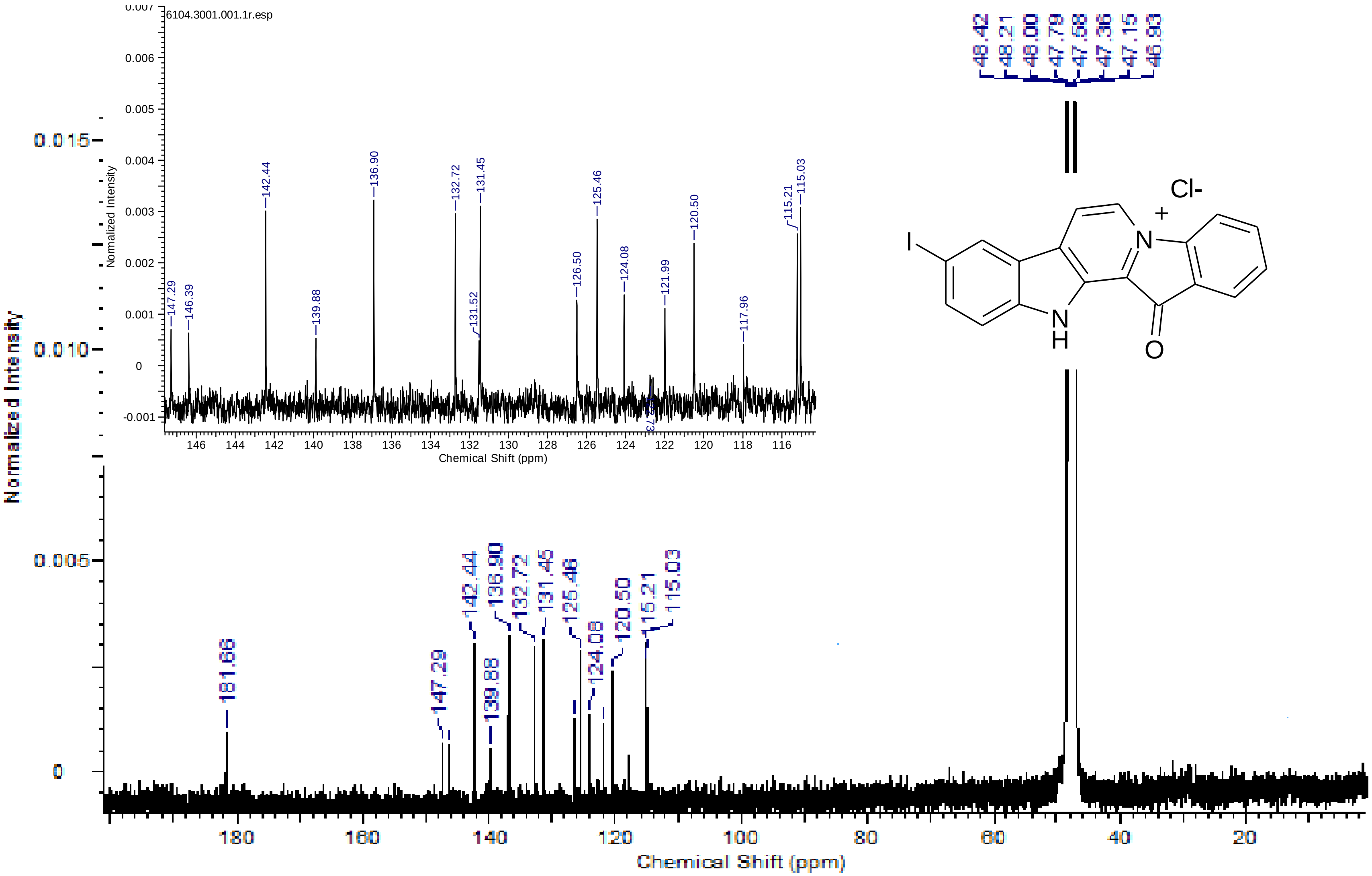

^{1}H NMR spectra of 3,10-dibromofascaplysin

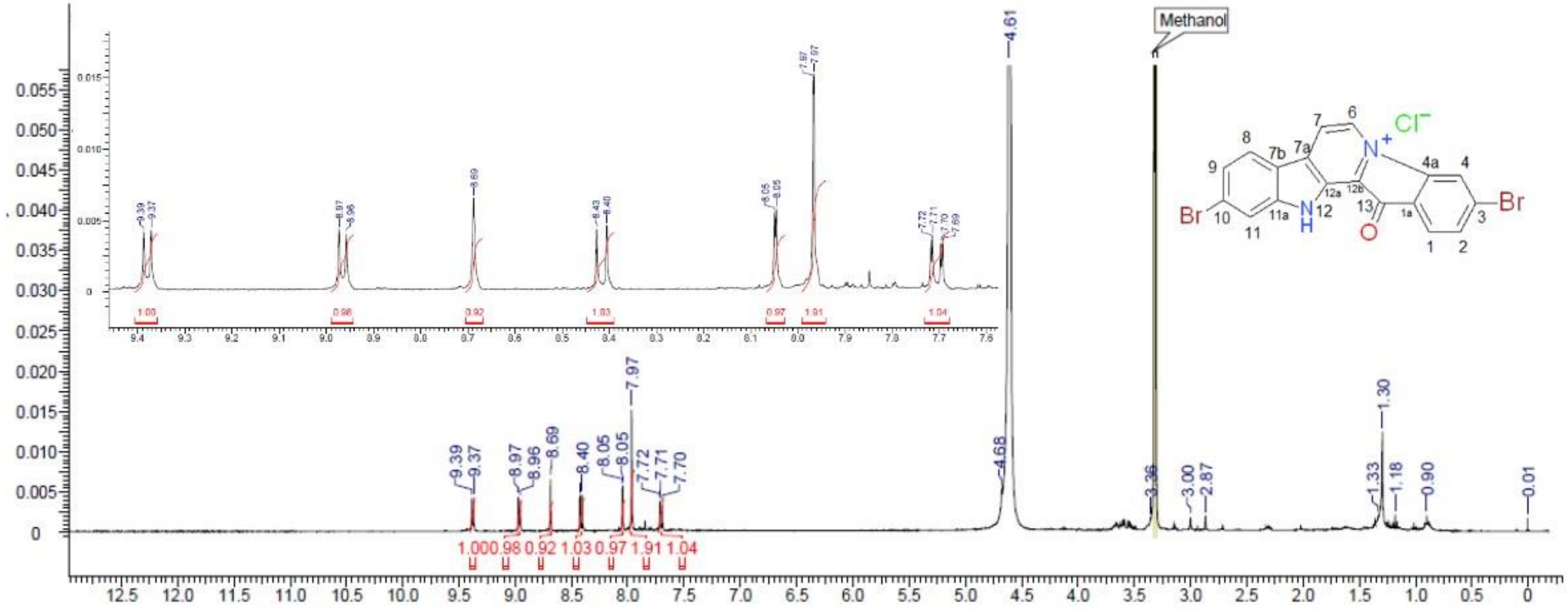

^{13}C NMR spectra of 3,10-dibromofascaplysin

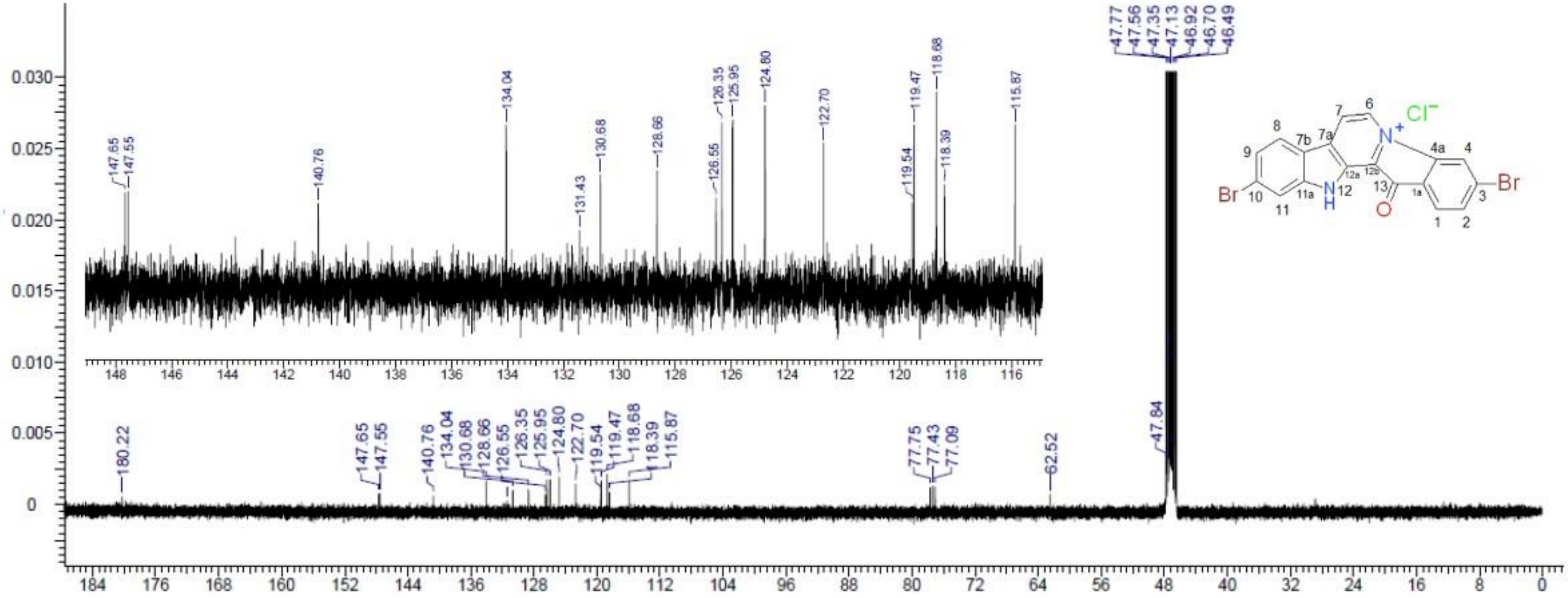

^{1}H NMR spectra of 12,13-dihydro-3,8-dibromo-13-oxopyrido[1,2-*a*:3,4-*b'*]diindol-5-ium chloride

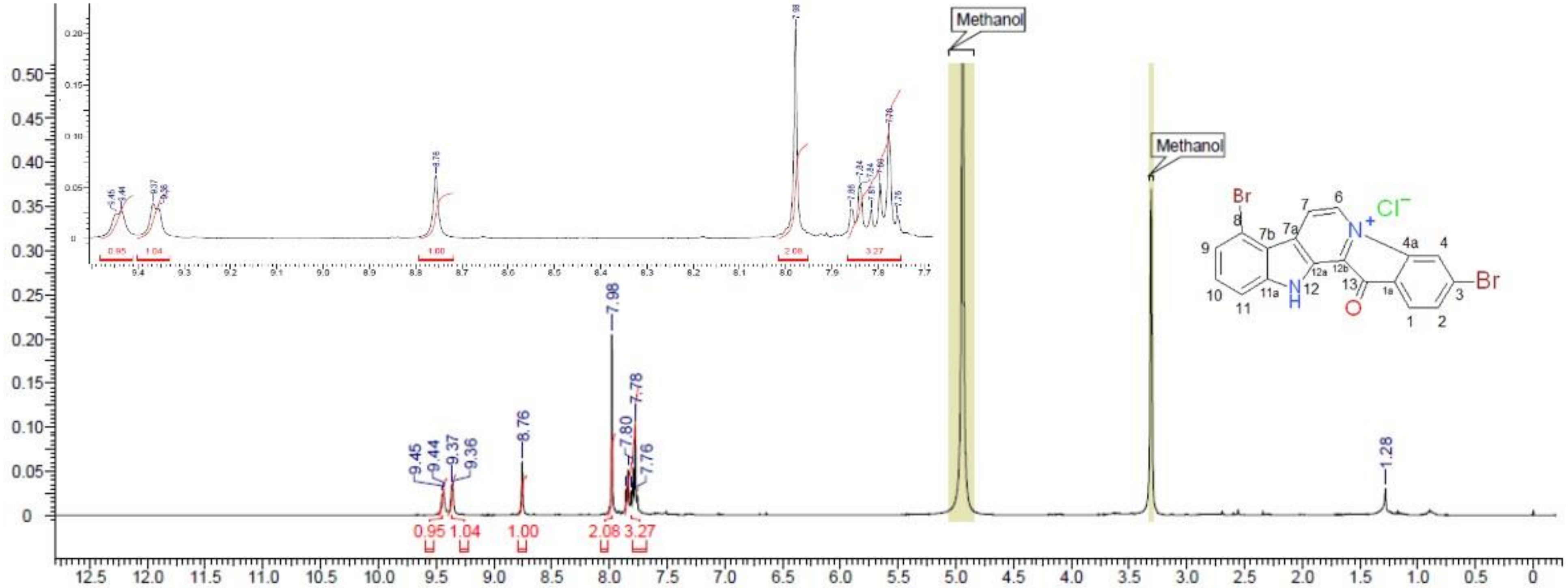

^{13}C NMR spectra of 12,13-dihydro-3,8-dibromo-13-oxopyrido[1,2-*a*:3,4-*b'*]diindol-5-ium chloride

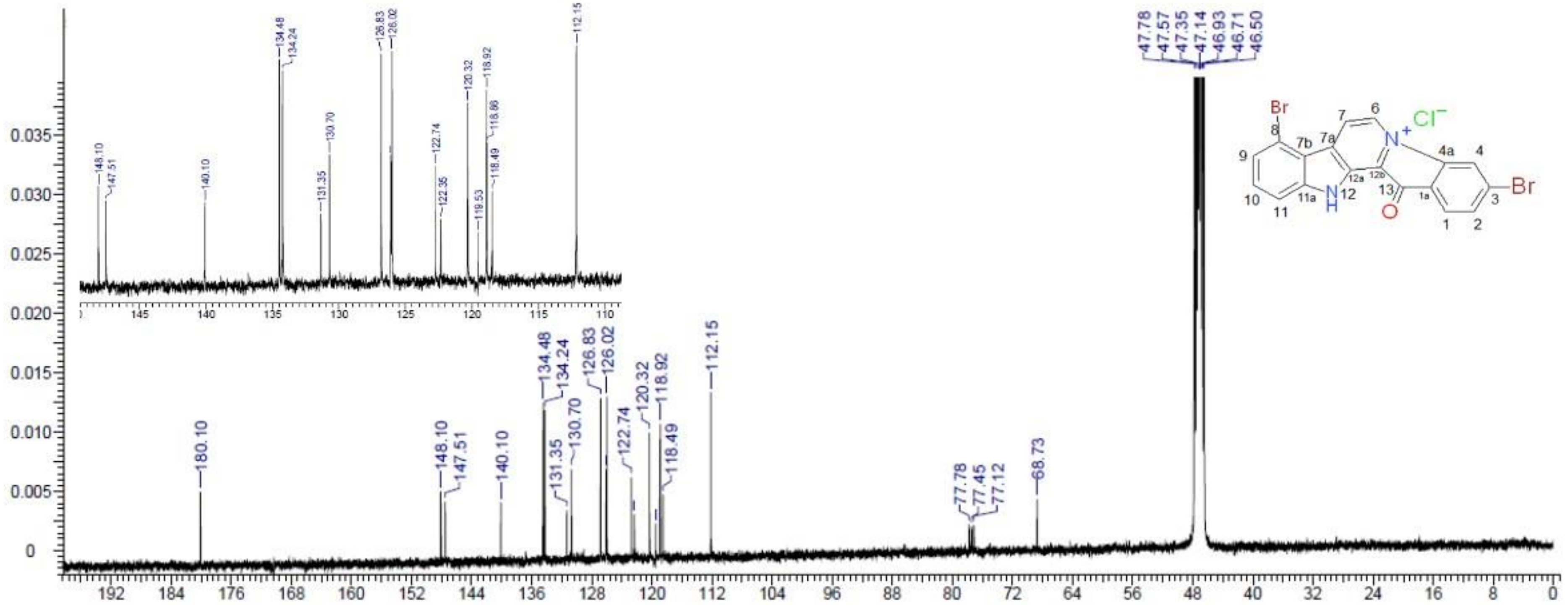

^{1}H NMR spectra of 12,13-dihydro-2,9-dibromo-13-oxopyrido[1,2-*a*:3,4-*b'*]diindol-5-ium chloride

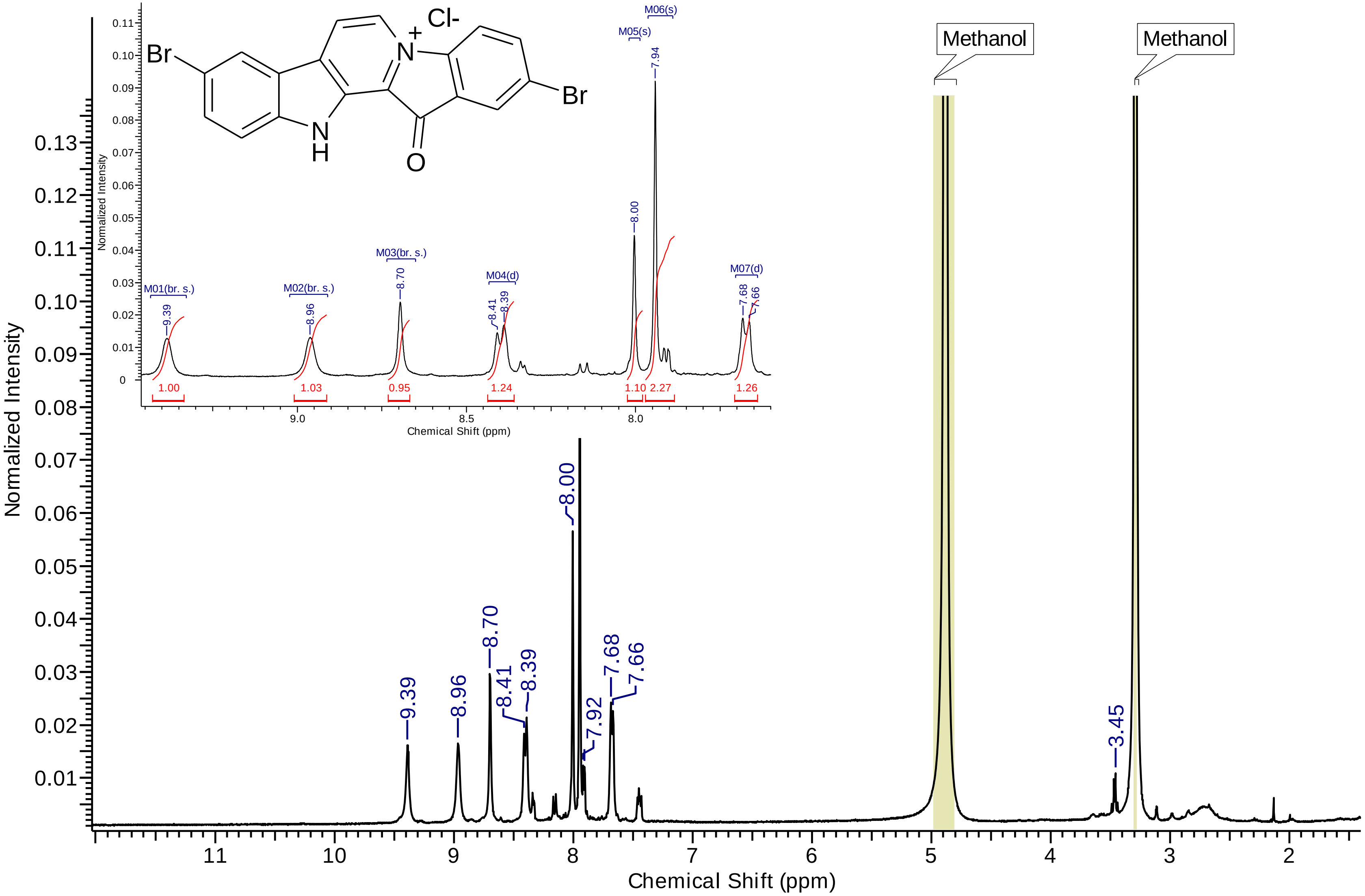

^{13}C NMR spectra of 12,13-dihydro-2,9-dibromo-13-oxopyrido[1,2-*a*:3,4-*b'*]diindol-5-ium chloride

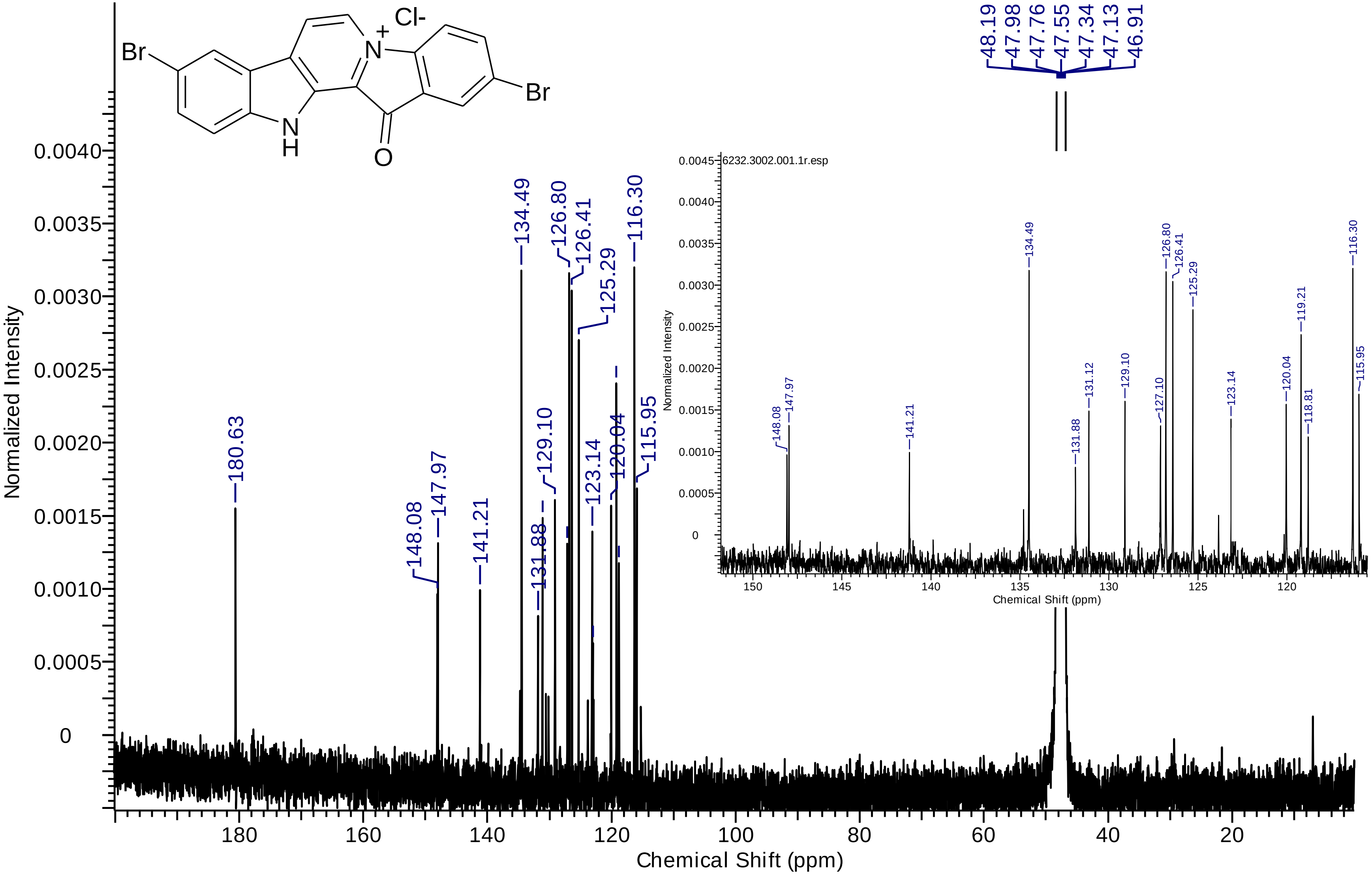

^{1}H NMR spectra of 12,13-dihydro-3,9-dibromo-13-oxopyrido[1,2-*a*:3,4-*b'*]diindol-5-ium chloride

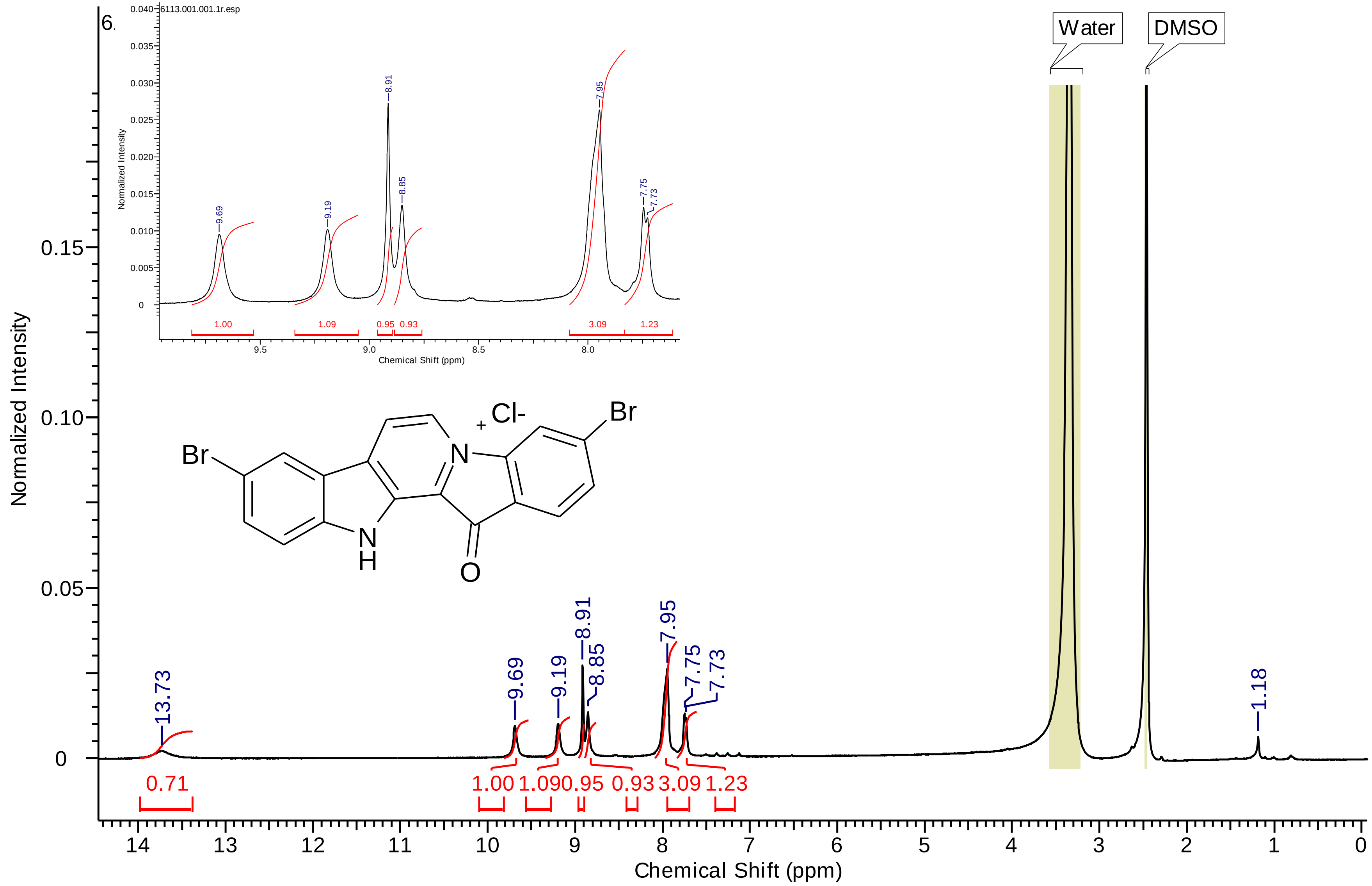

^{13}C NMR spectra of 12,13-dihydro-3,9-dibromo-13-oxopyrido[1,2-*a*:3,4-*b'*]diindol-5-ium chloride

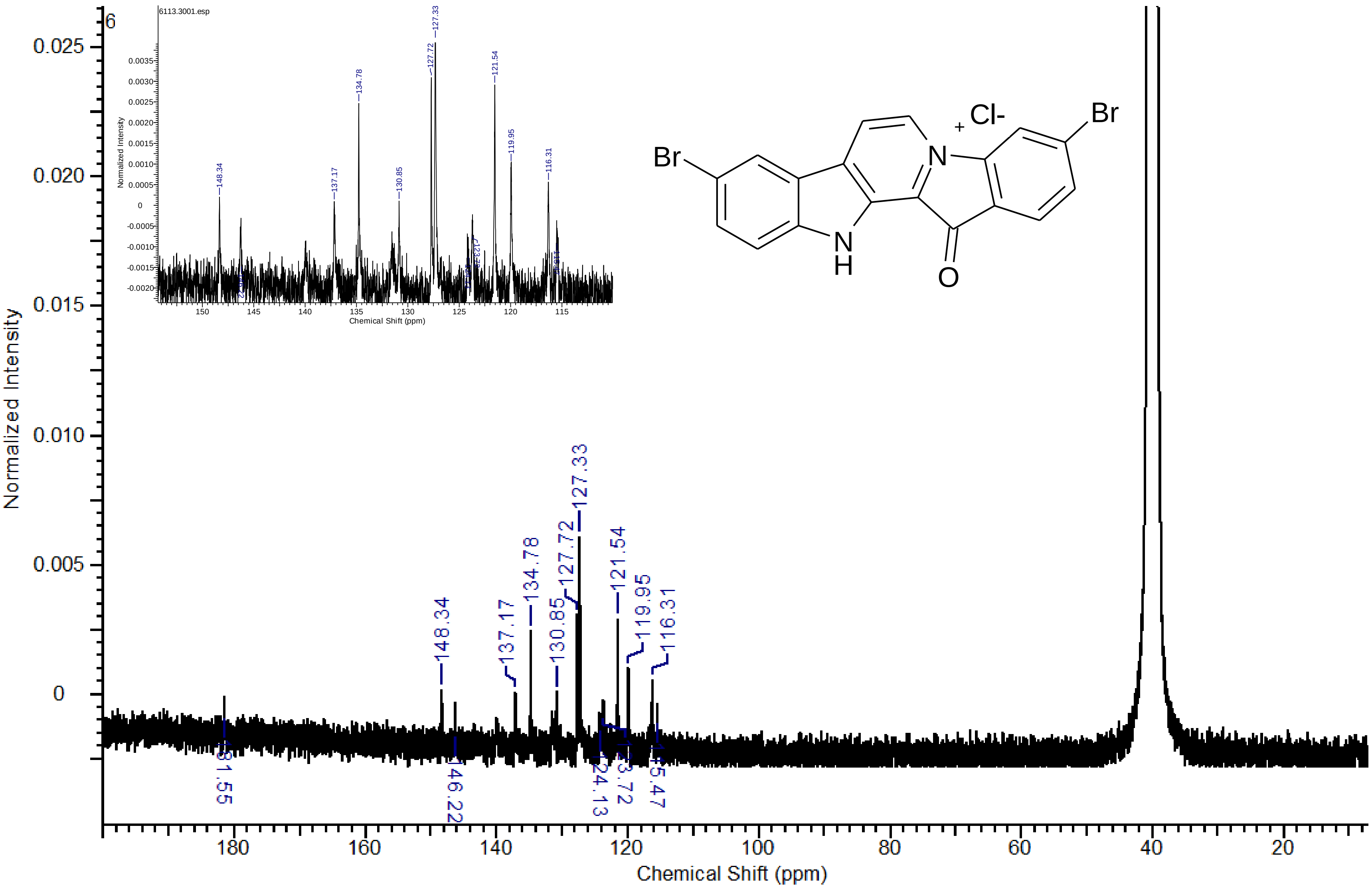

^{1}H NMR spectra of 12,13-dihydro-2,9,11-tribromo-13-oxopyrido[1,2-*a*:3,4-*b'*]diindol-5-ium chloride

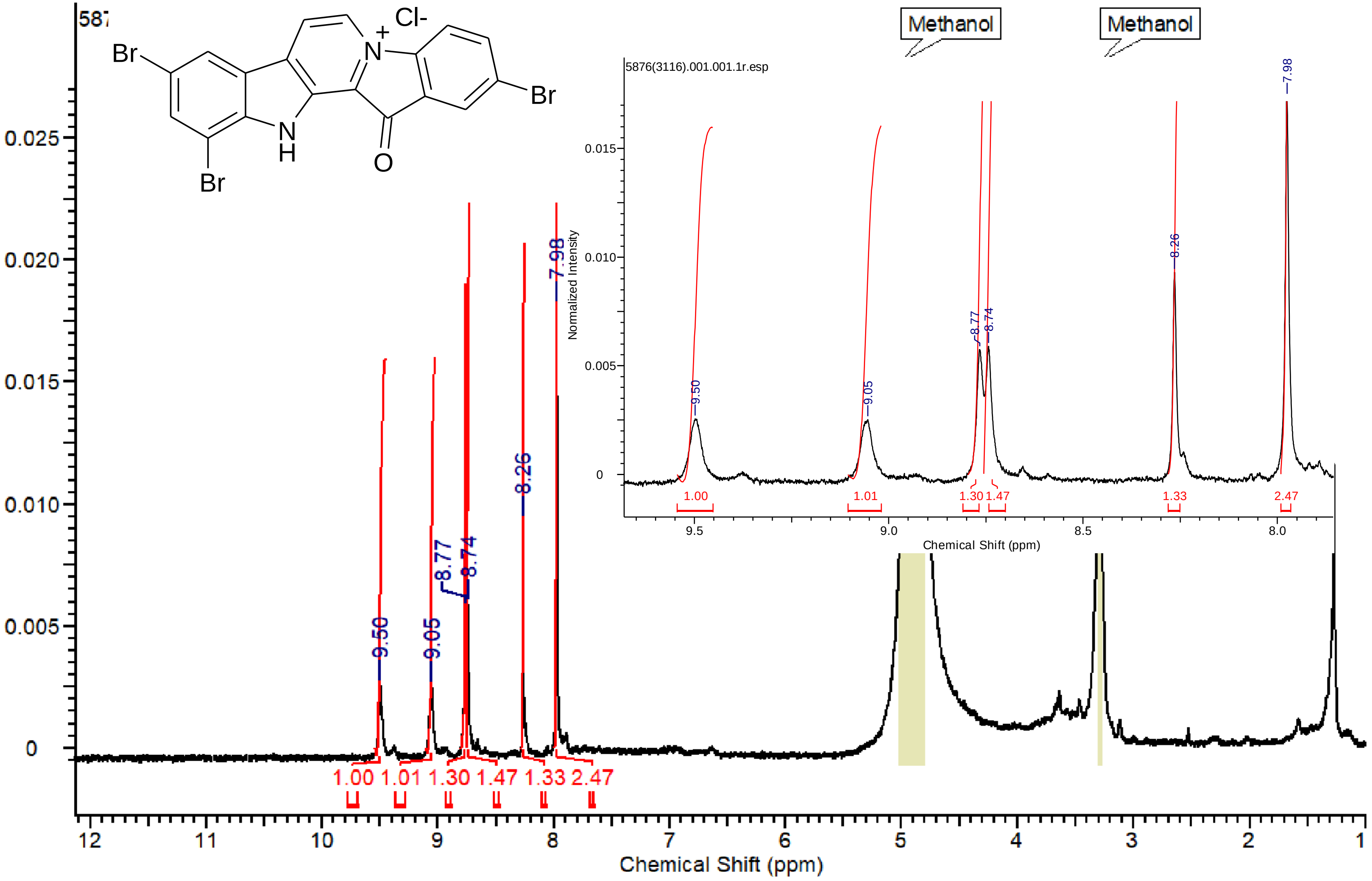

^{13}C NMR spectra of 12,13-dihydro-2,9,11-tribromo-13-oxopyrido[1,2-*a*:3,4-*b'*]diindol-5-ium chloride

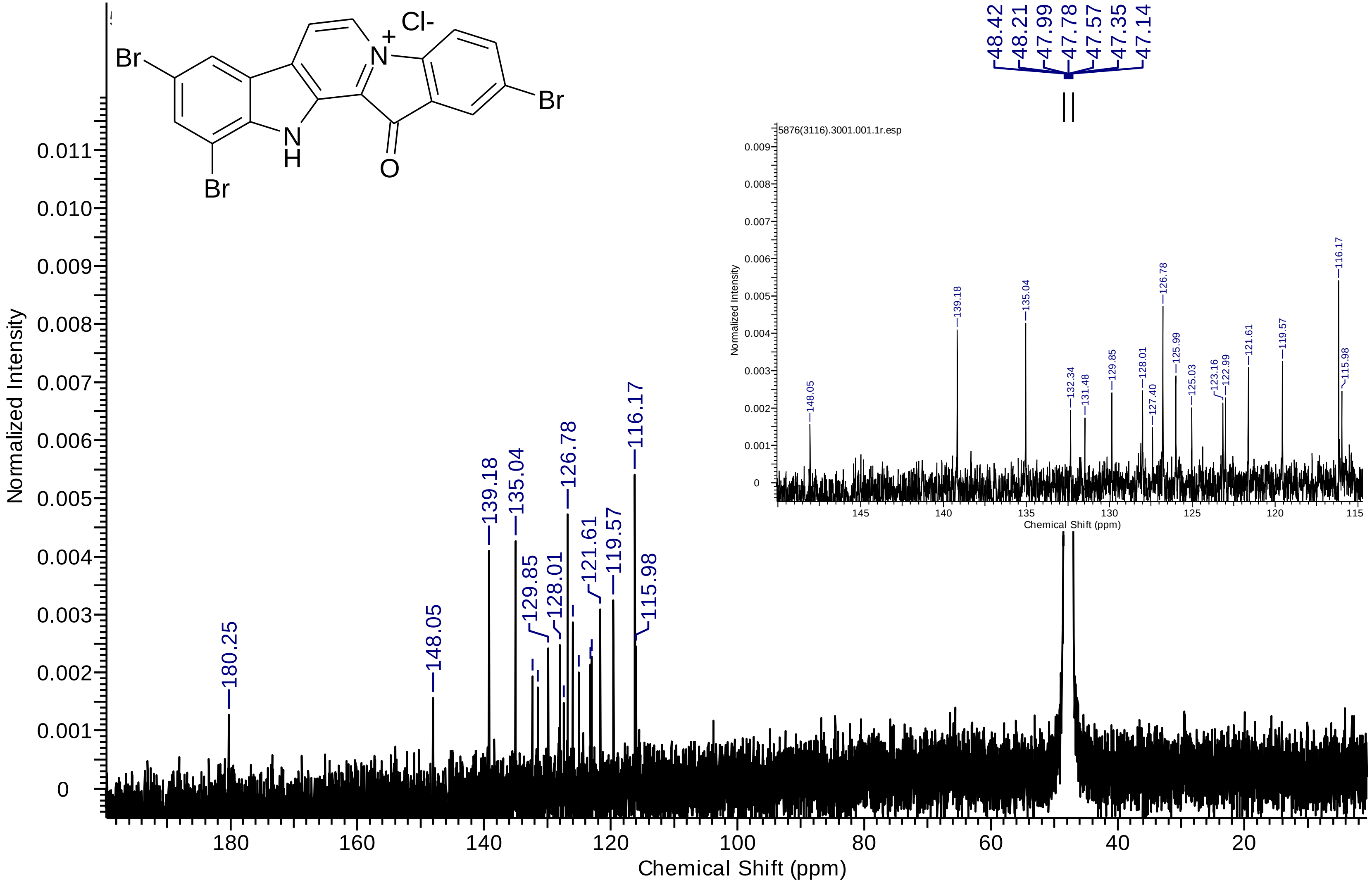

^{1}H NMR spectra of 12,13-dihydro-2-bromo-9-iodo-13-oxopyrido[1,2-*a*:3,4-*b*’]diindol-5-ium chloride

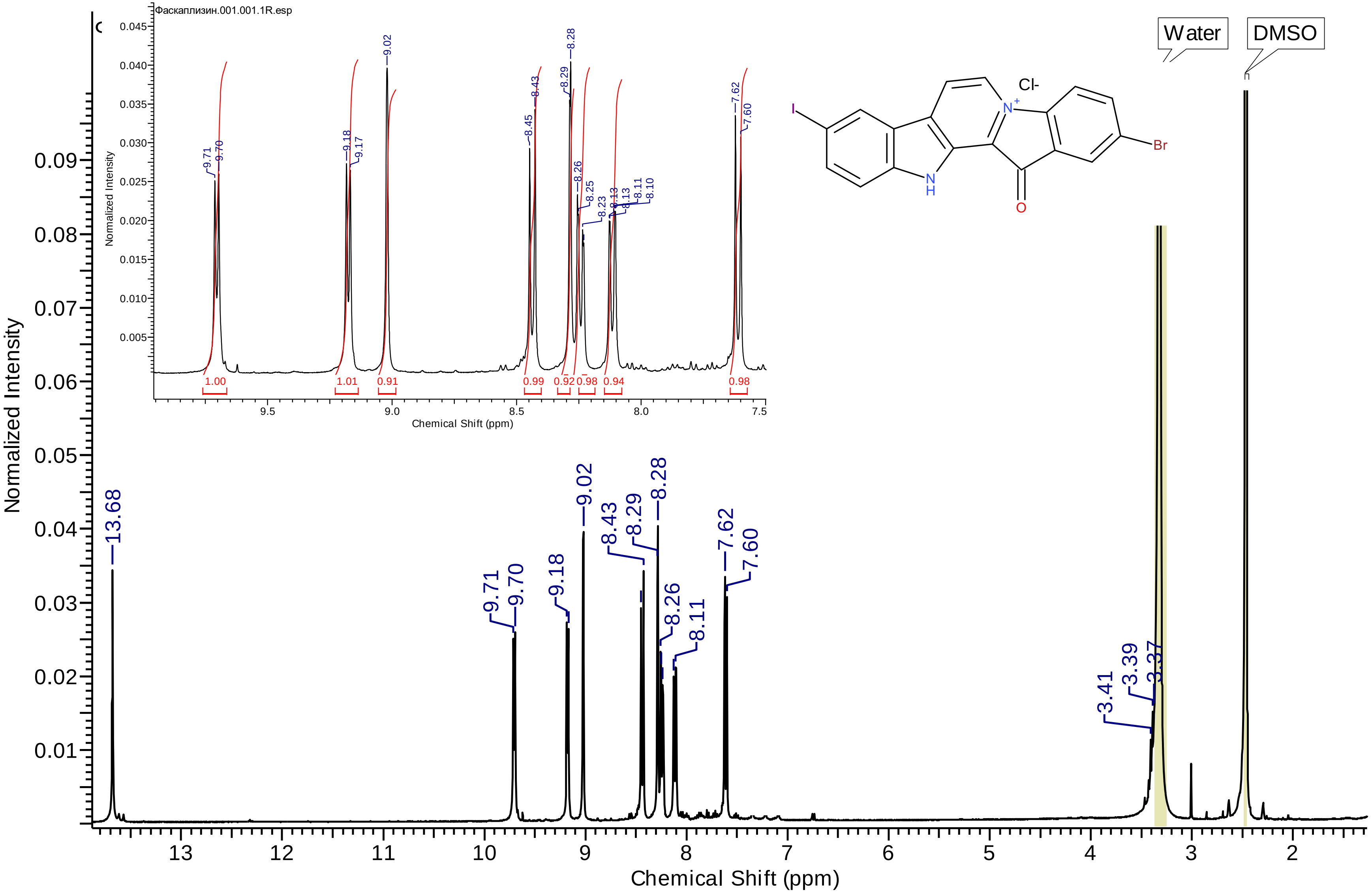

^{13}C NMR spectra of 12,13-dihydro-2-bromo-9-iodo-13-oxopyrido[1,2-*a*:3,4-*b'*]diindol-5-ium chloride

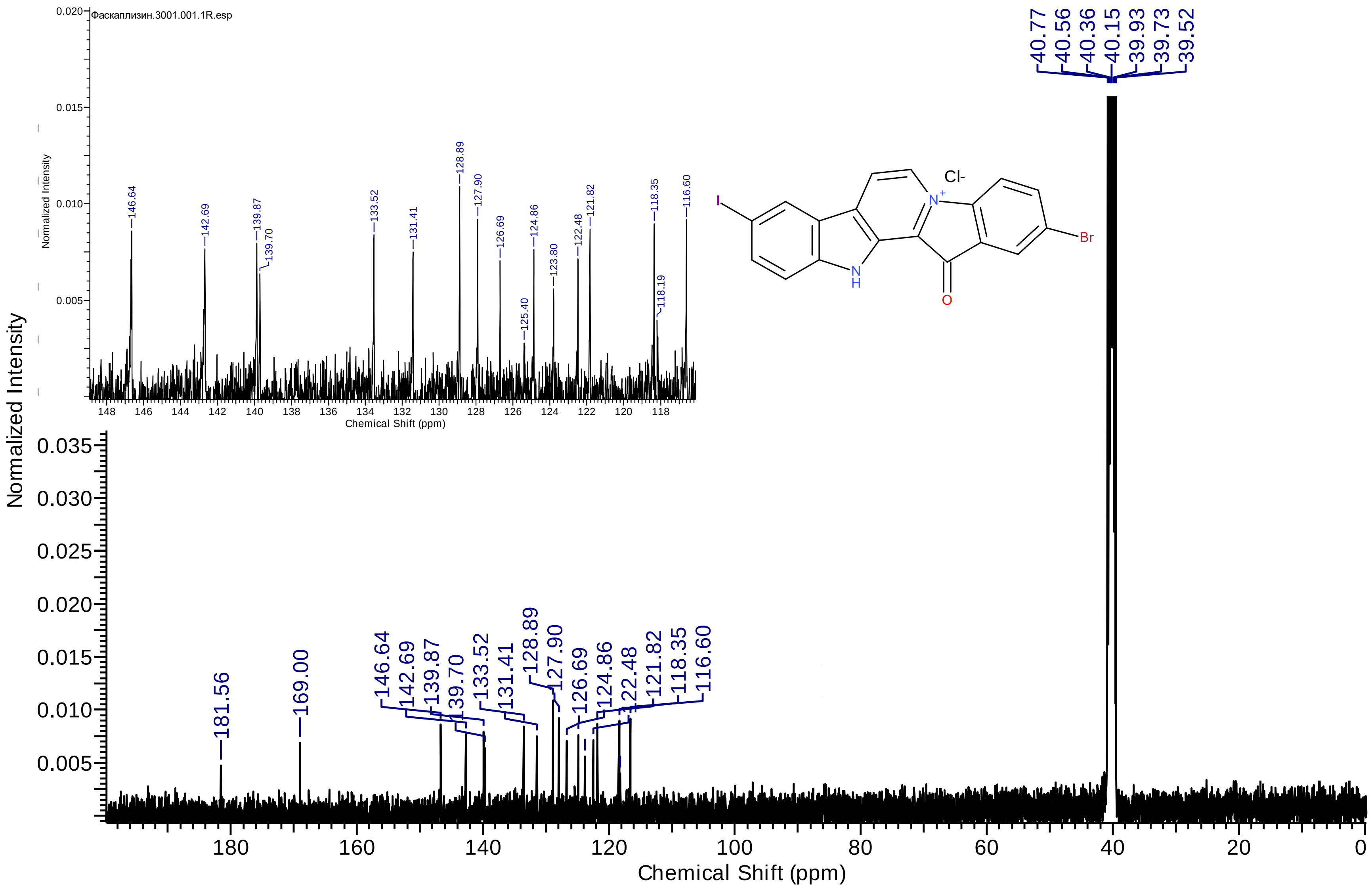

^{1}H NMR spectra of 12,13-dihydro-2-bromo-9-chloro-13-oxopyrido[1,2-*a*:3,4-*b'*]diindol-5-ium chloride

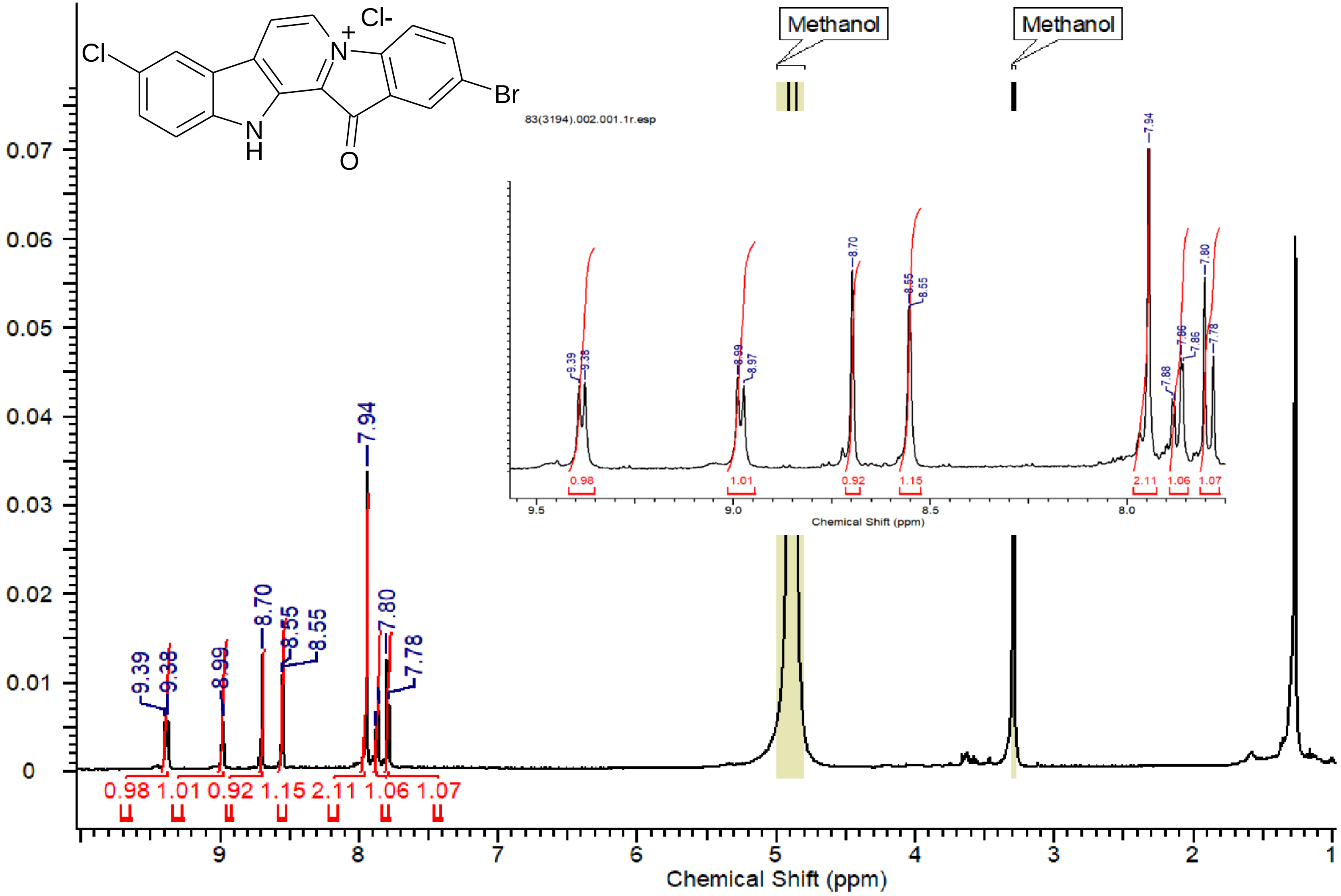

^{13}C NMR spectra of 12,13-dihydro-2-bromo-9-chloro-13-oxopyrido[1,2-*a*:3,4-*b'*]diindol-5-ium chloride

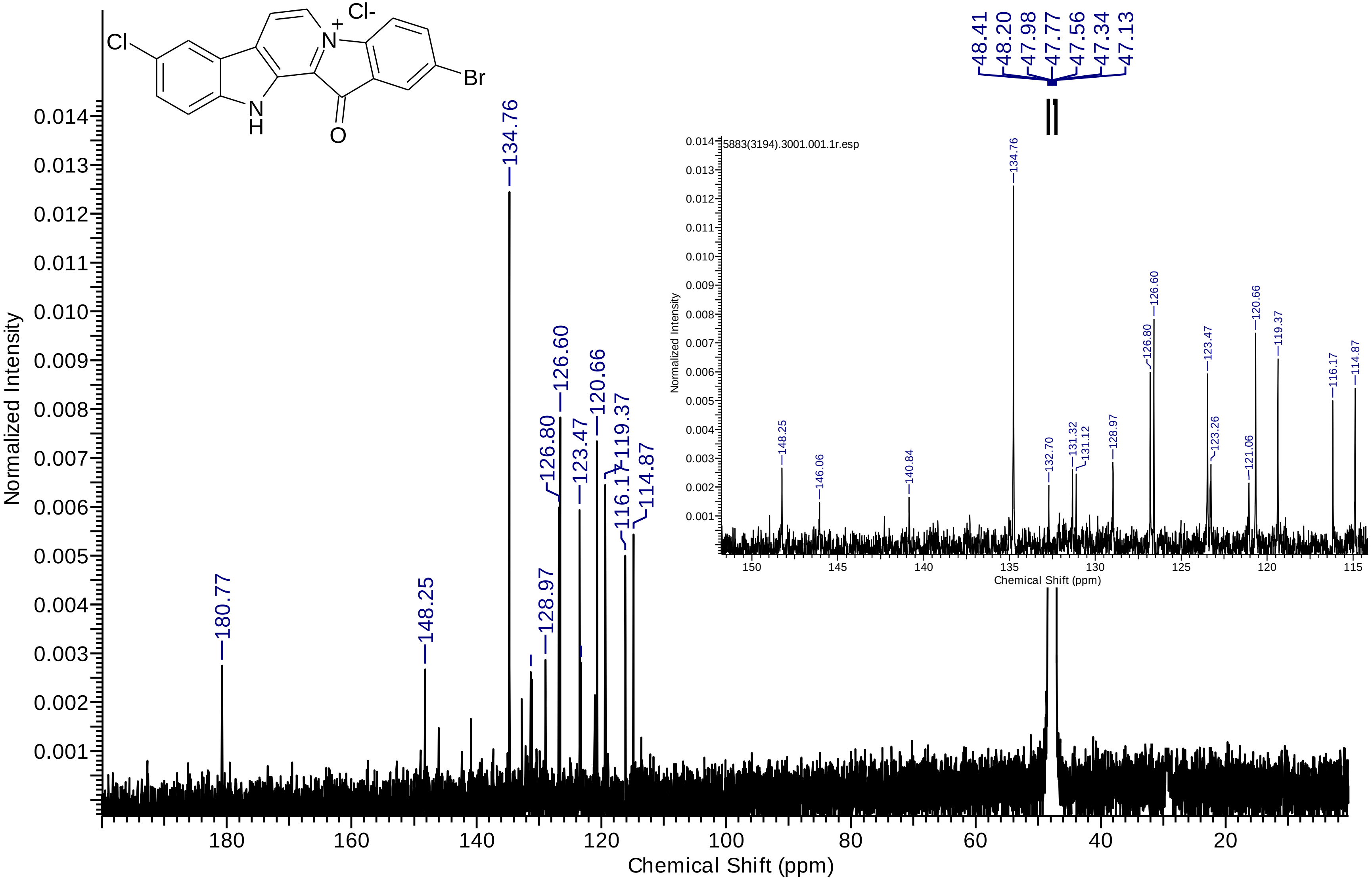

^{1}H NMR spectra of 12,13-dihydro-2-bromo-9,11-dichloro-13-oxopyrido[1,2-*a*:3,4-*b'*]diindol-5-ium chloride

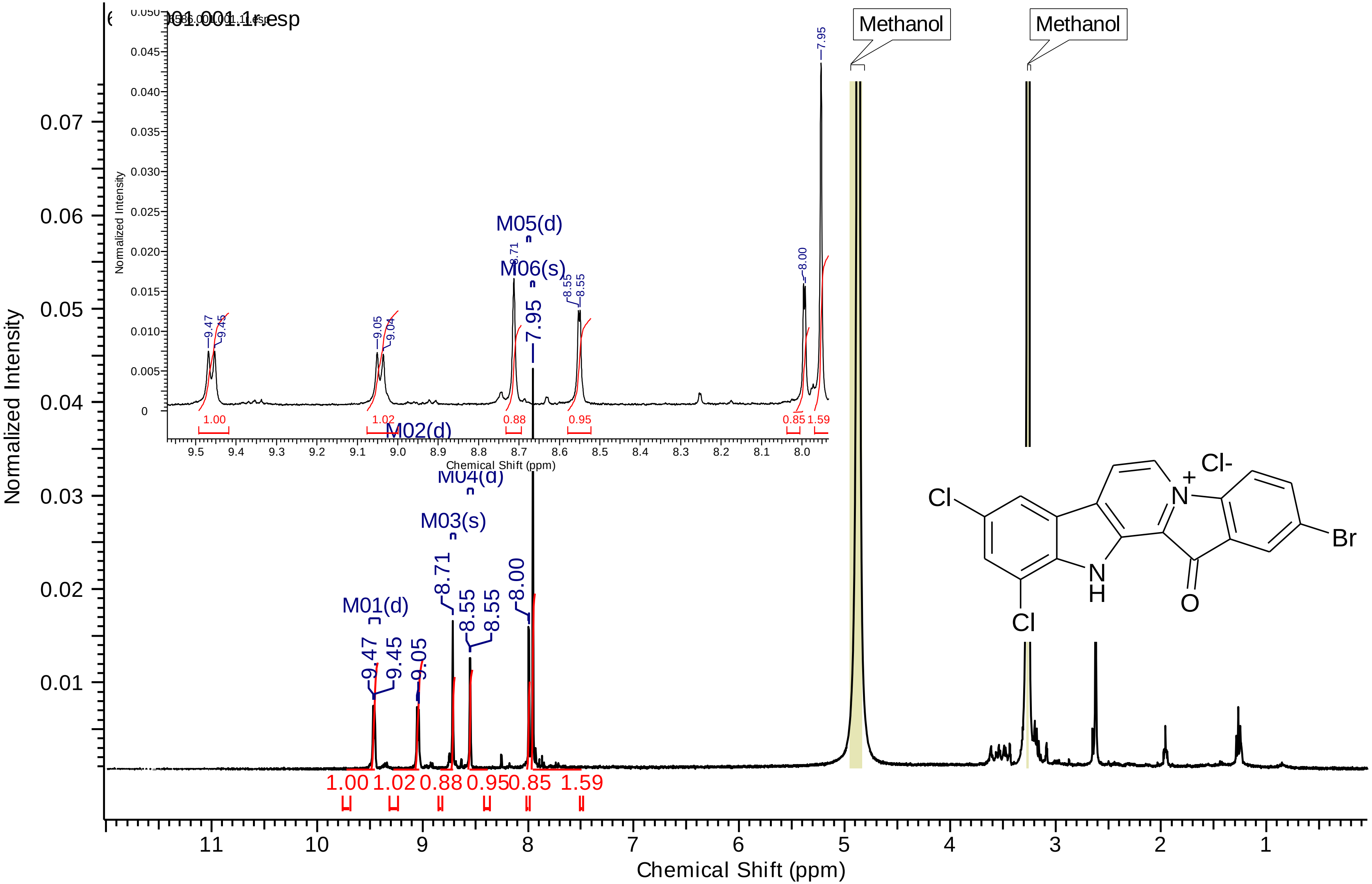

^{13}C NMR spectra of 12,13-dihydro-2-bromo-9,11-dichloro-13-oxopyrido[1,2-*a*:3,4-*b'*]diindol-5-ium chloride

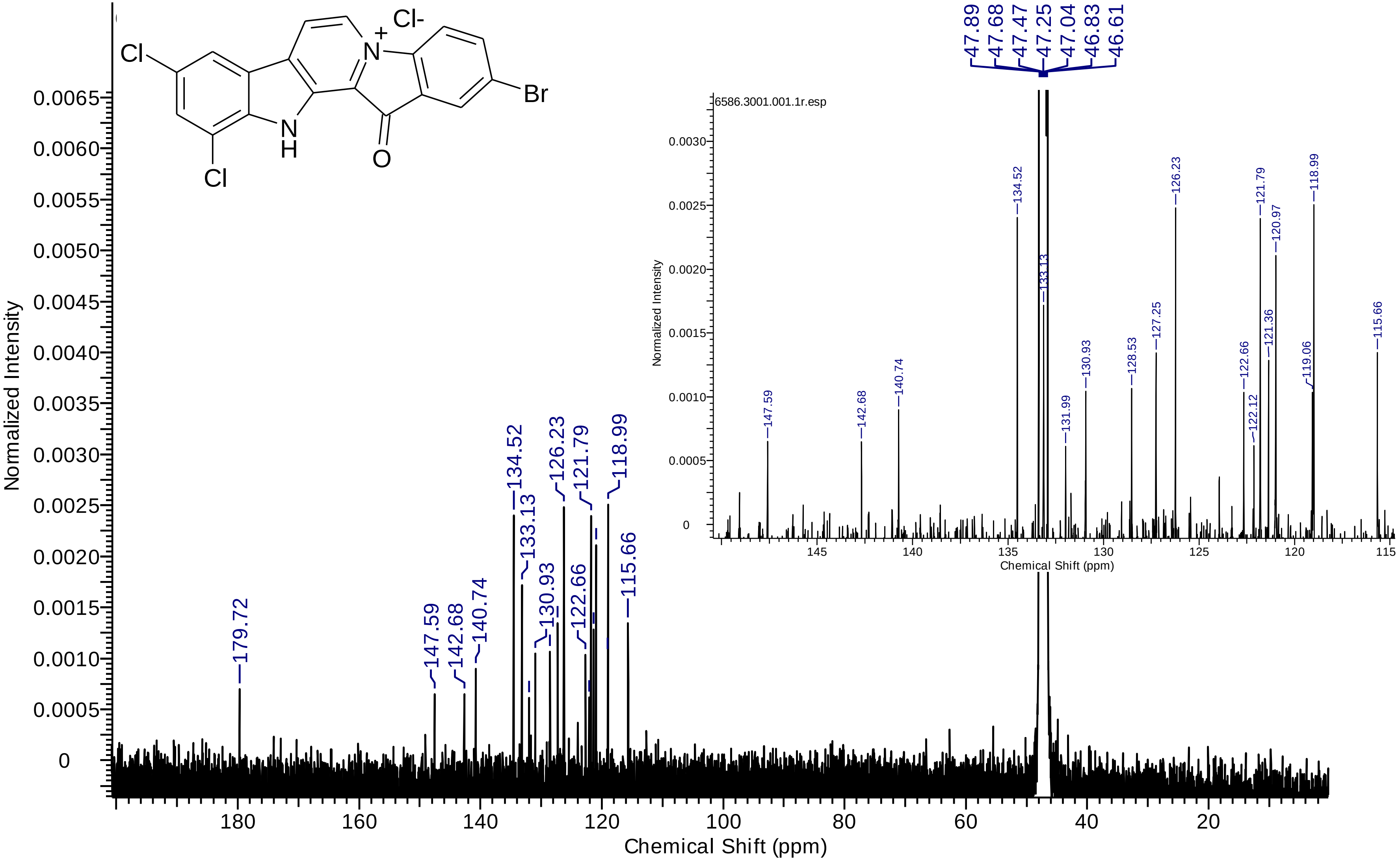

^{1}H NMR spectra of 7-phenyl-6,13-dioxopyrido[1,2-*a*:3,4-*b'*]diindole

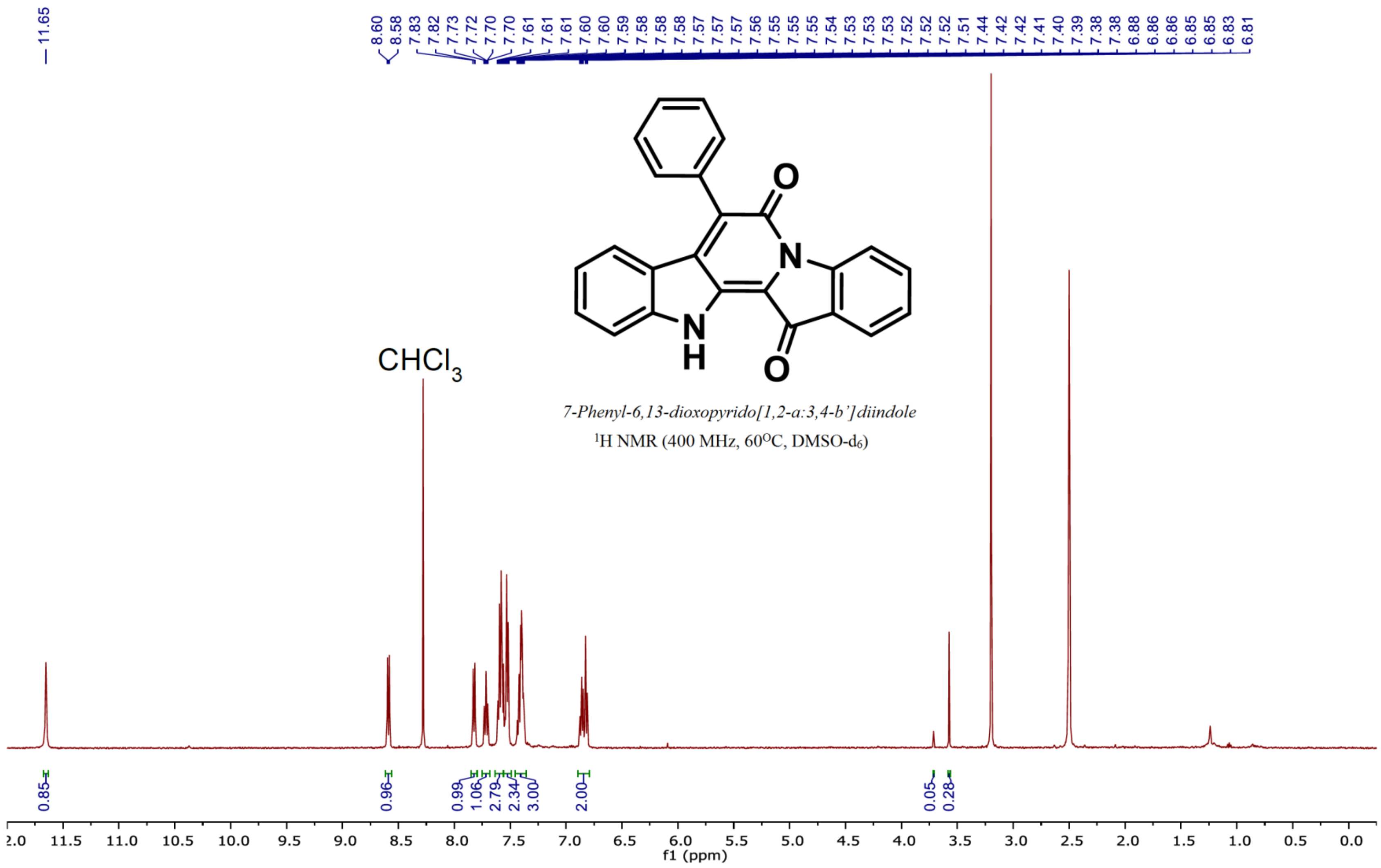

^{13}C NMR spectra of 7-phenyl-6,13-dioxopyrido[1,2-*a*:3,4-*b'*]diindole

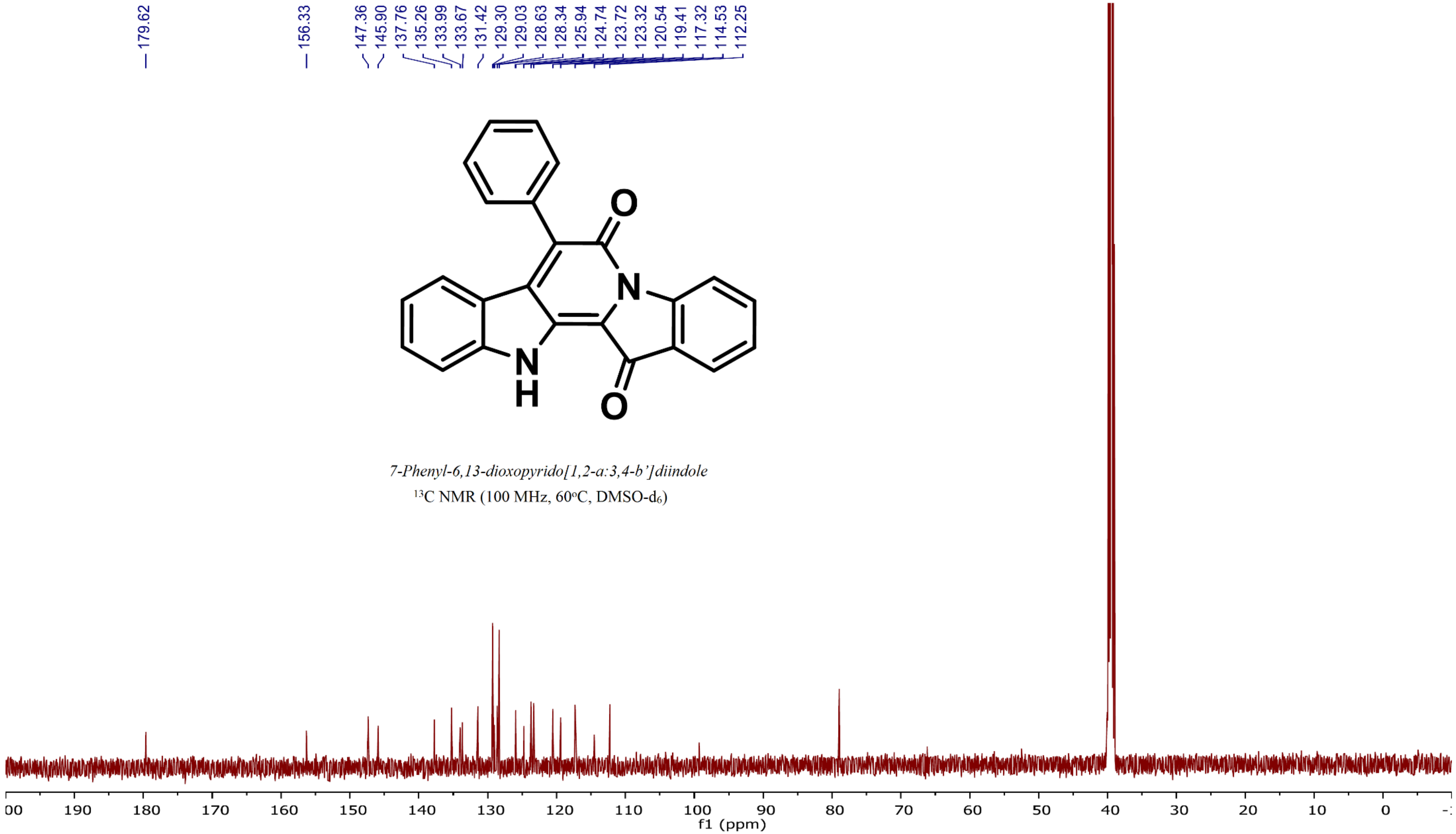

^{1}H NMR spectra of 7-ethyl-6,13-dioxopyrido[1,2-*a*:3,4-*b'*]diindole

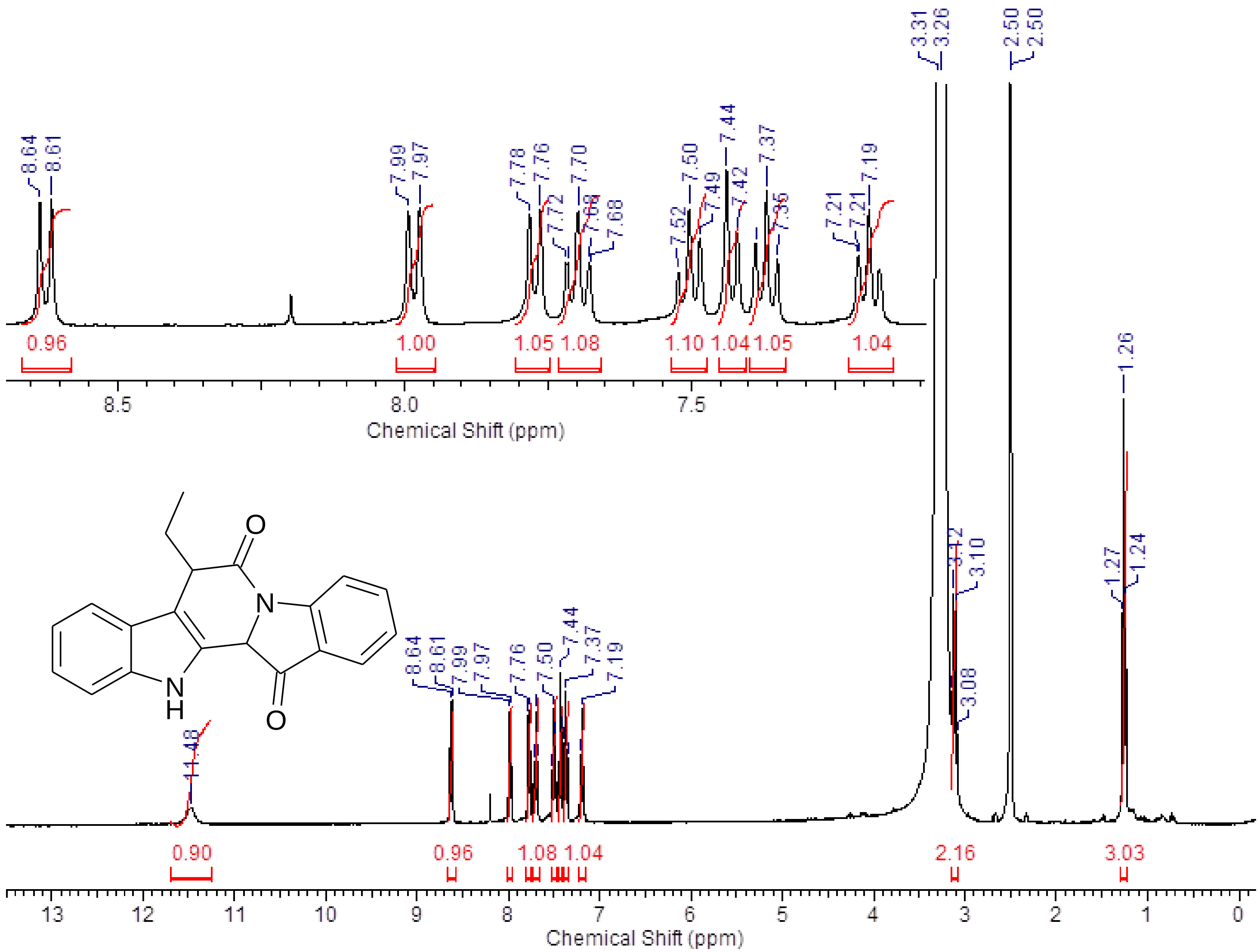

^{13}C NMR spectra of 7-ethyl-6,13-dioxopyrido[1,2-*a*:3,4-*b'*]diindole

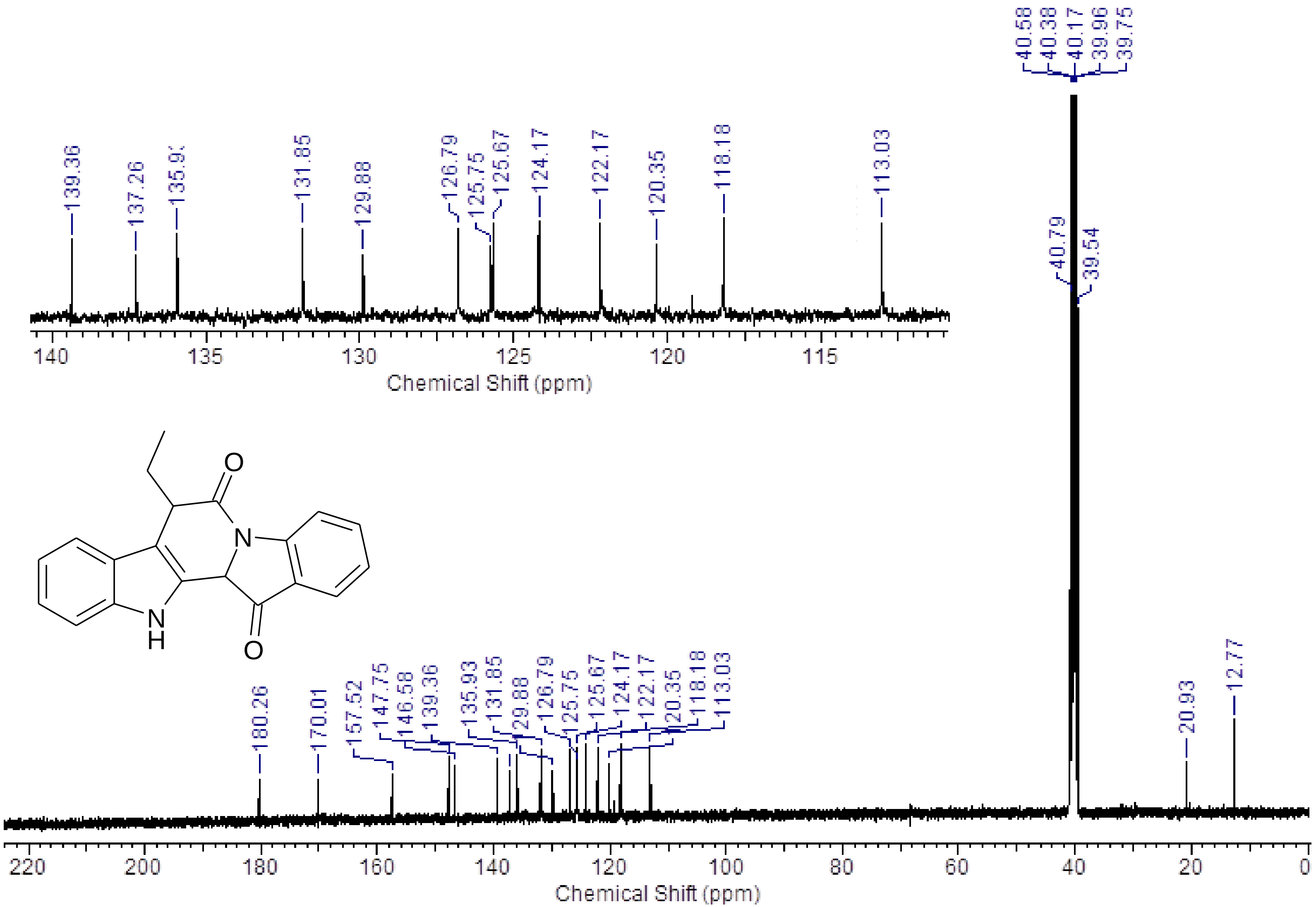

^{1}H NMR spectra of 7-methyl-6,13-dioxopyrido[1,2-*a*:3,4-*b'*]diindole

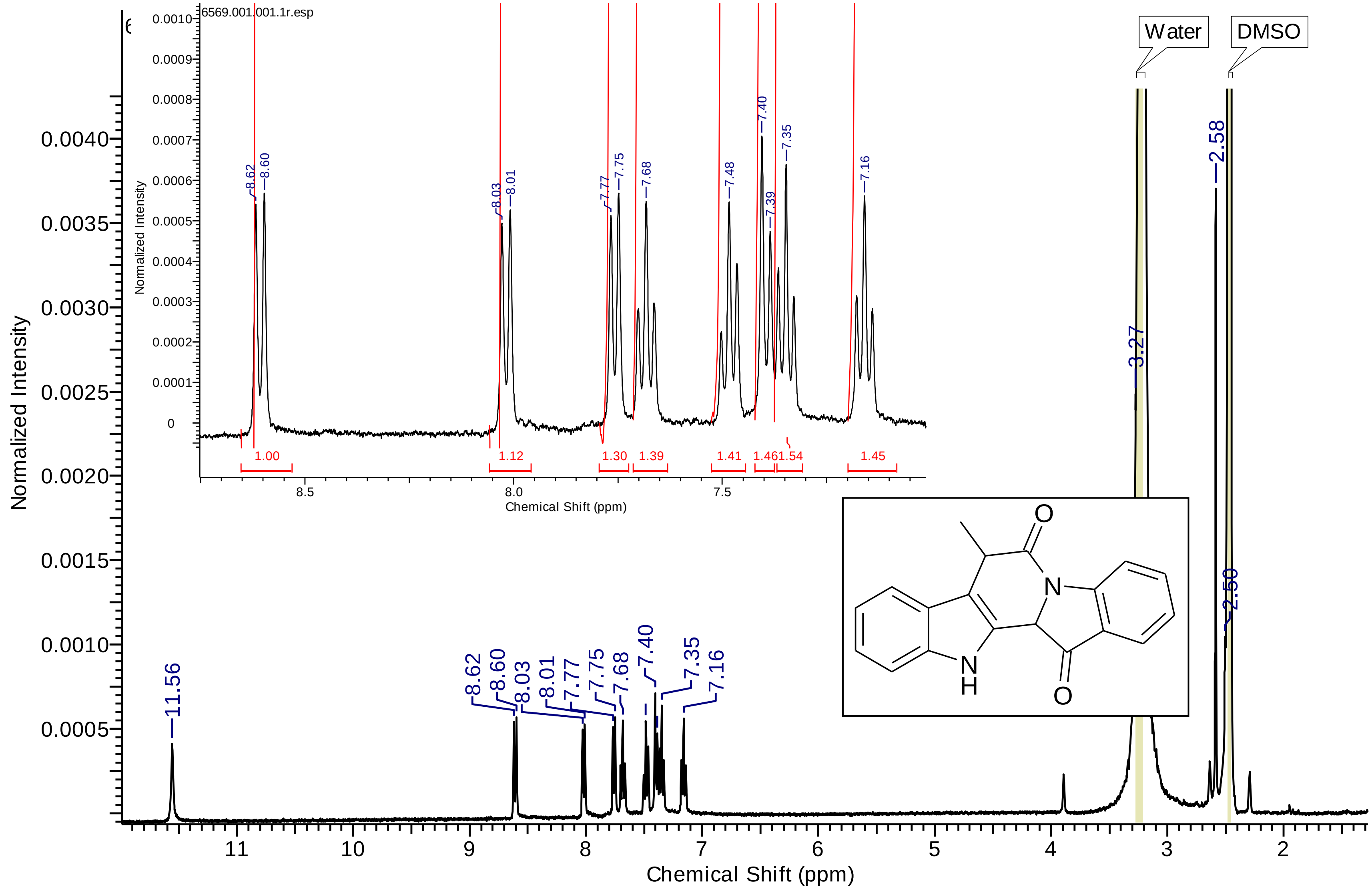

^{13}C NMR spectra of 7-methyl-6,13-dioxopyrido[1,2-*a*:3,4-*b'*]diindole

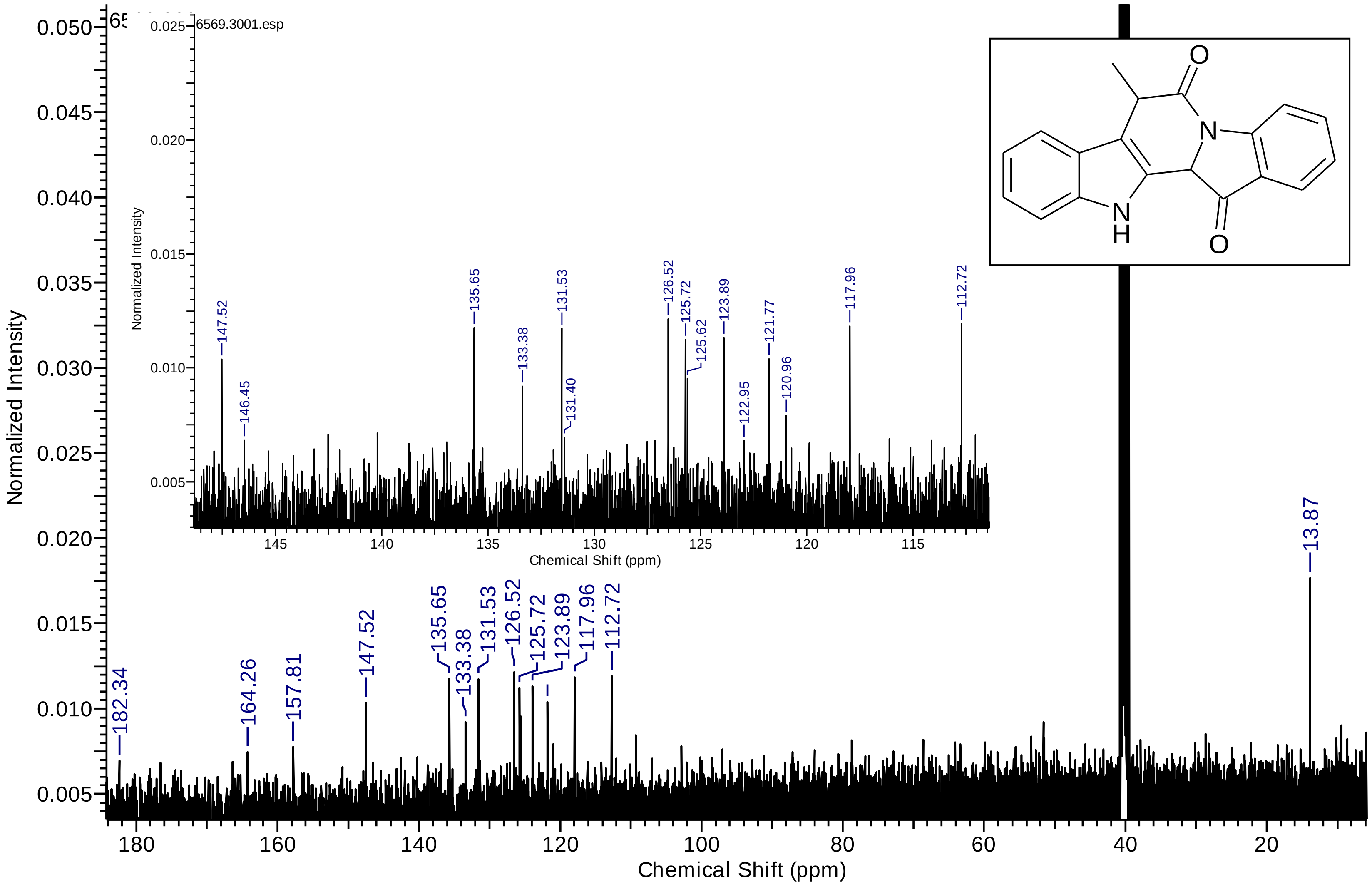

^{1}H NMR spectra of 12,13-dihydro-7-phenyl-13-oxopyrido[1,2-*a*:3,4-*b'*]diindol-5-ium chloride (16)

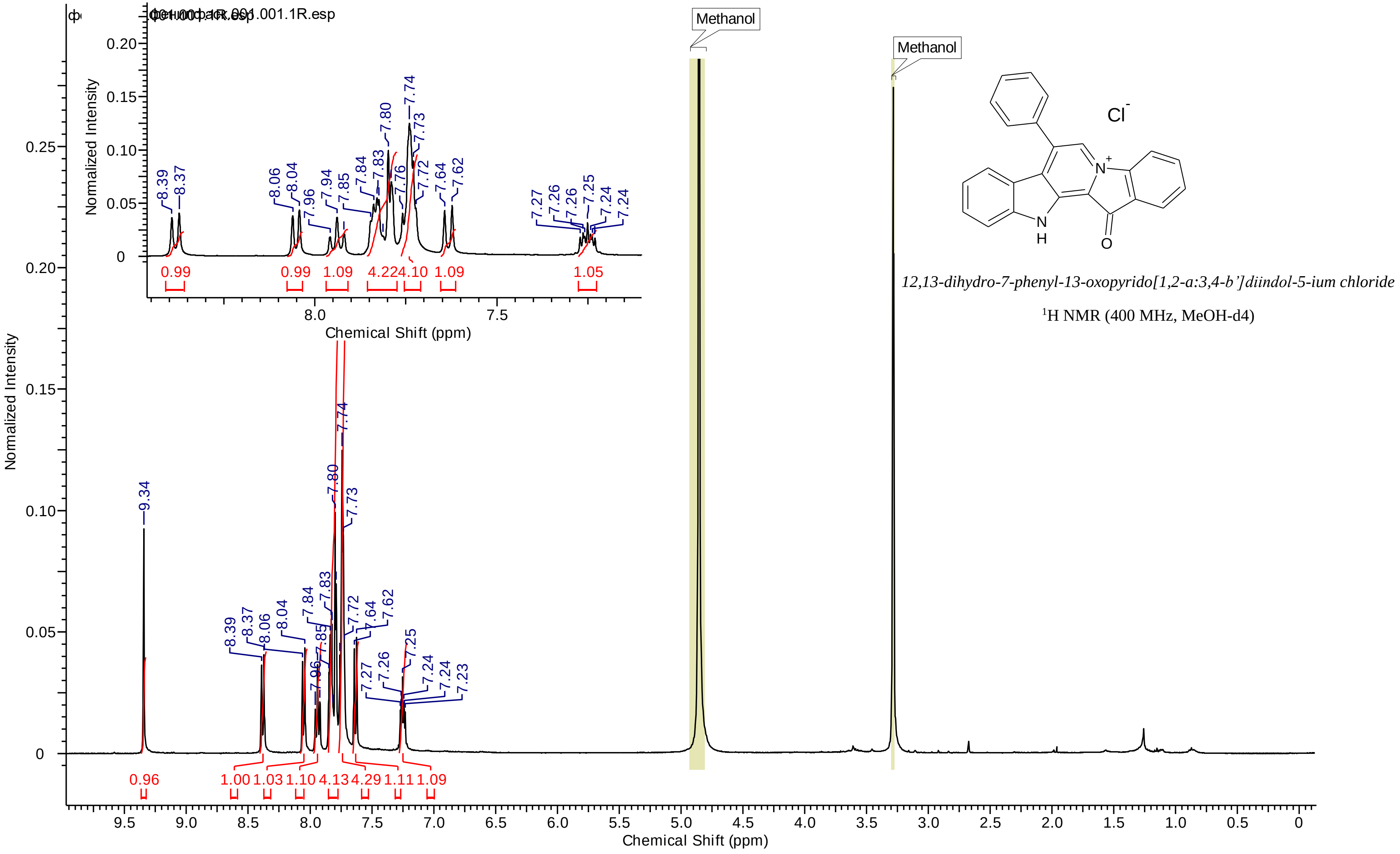

^{13}C NMR spectra of 12,13-dihydro-7-phenyl-13-oxopyrido[1,2-*a*:3,4-*b'*]diindol-5-ium chloride (16)

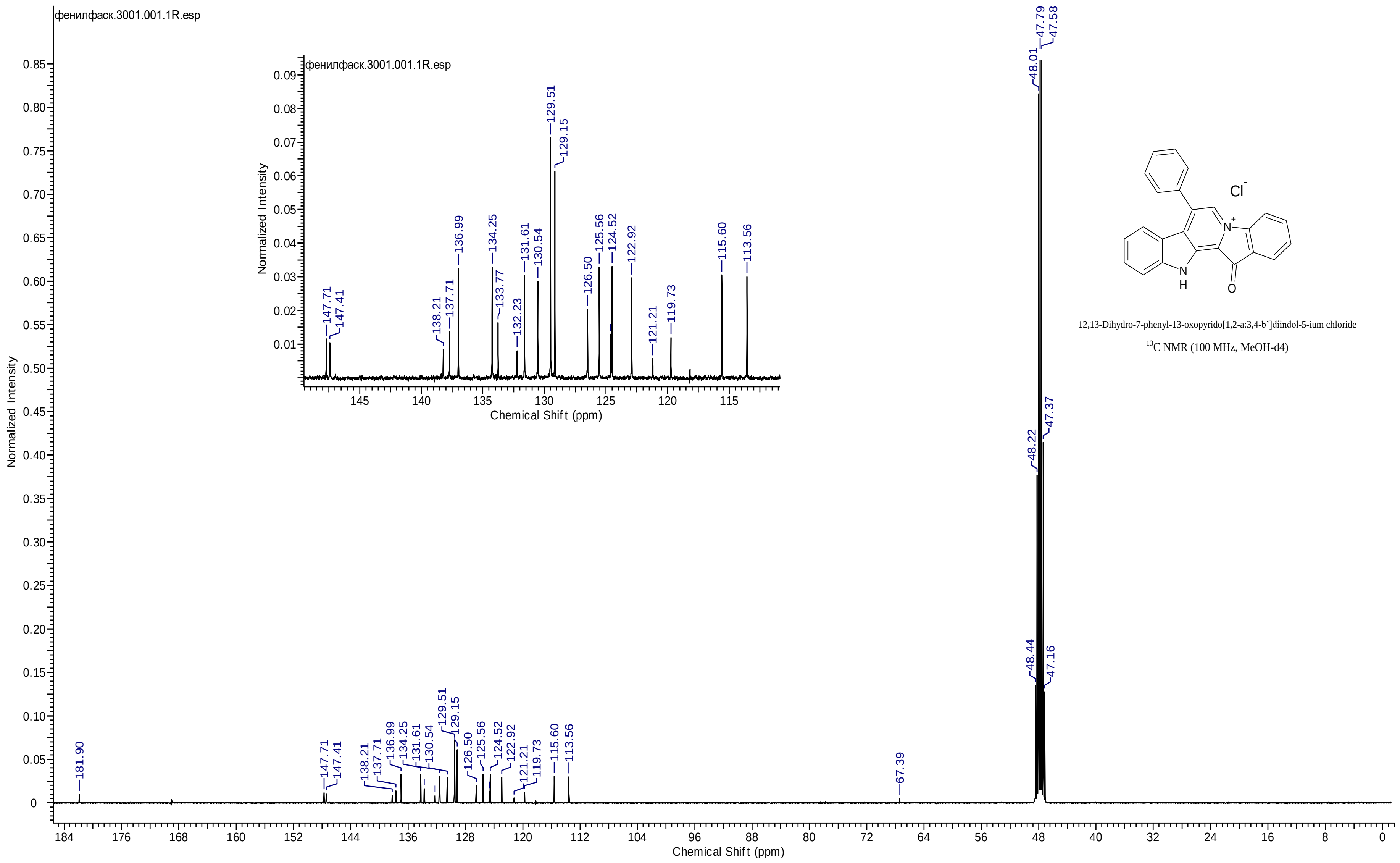

^{1}H NMR spectra of 12,13-dihydro-7-ethyl-13-oxopyrido[1,2-*a*:3,4-*b'*]diindol-5-ium chloride (17)

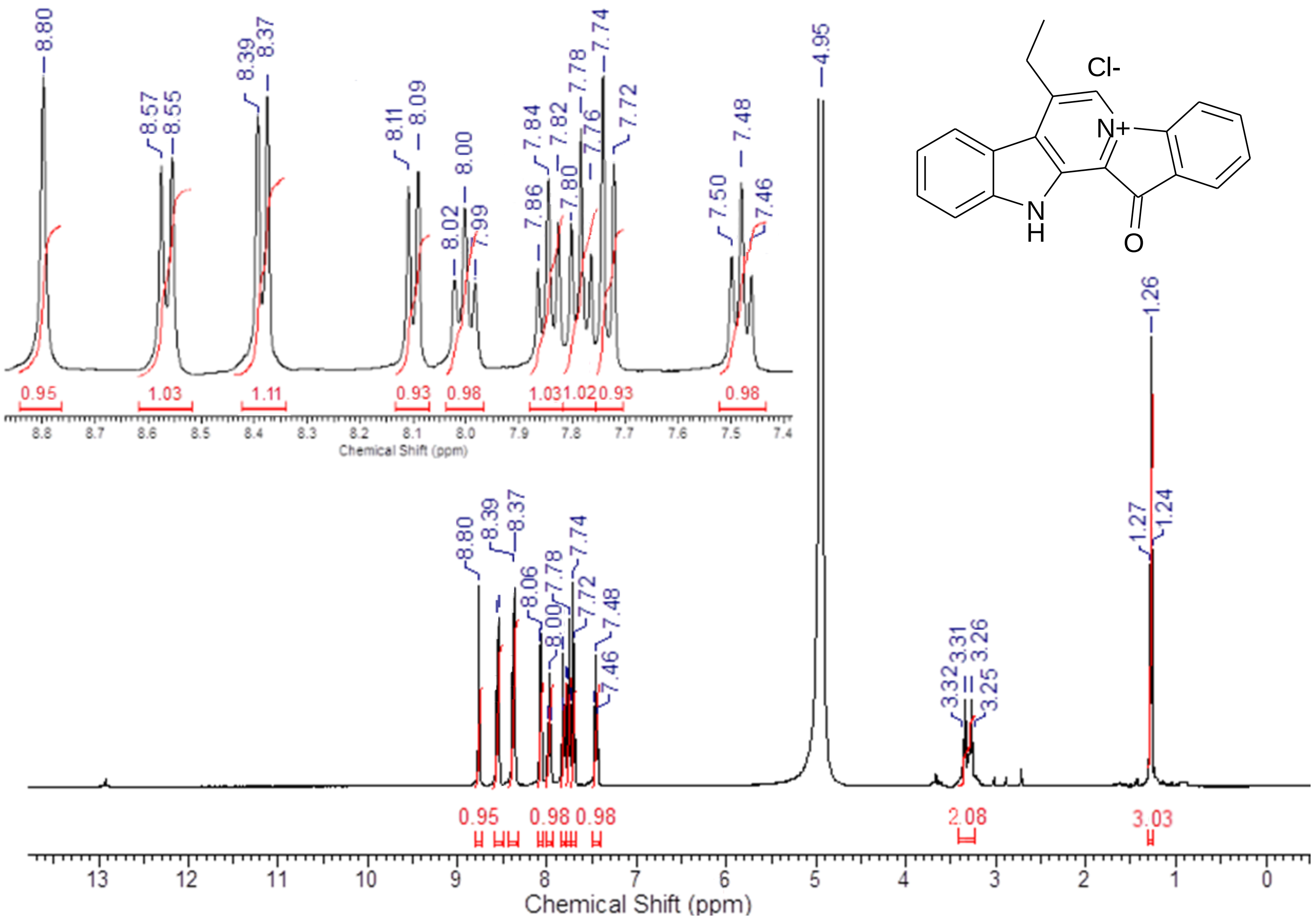

^{13}C NMR spectra of 12,13-dihydro-7-ethyl-13-oxopyrido[1,2-*a*:3,4-*b'*]diindol-5-ium chloride (17)

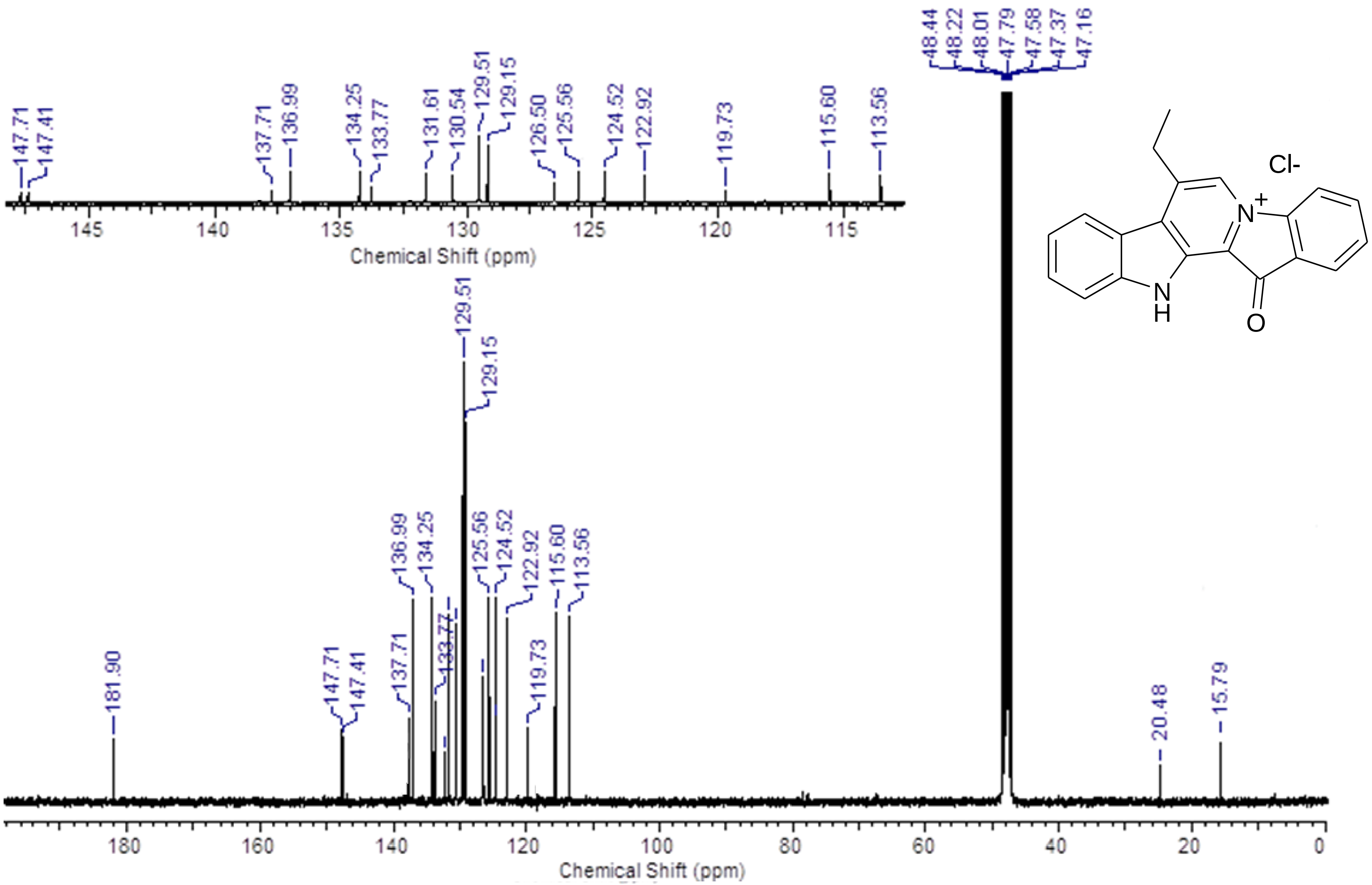

^{1}H NMR spectra of 12,13-dihydro-7-methyl-13-oxopyrido[1,2-*a*:3,4-*b'*]diindol-5-ium chloride

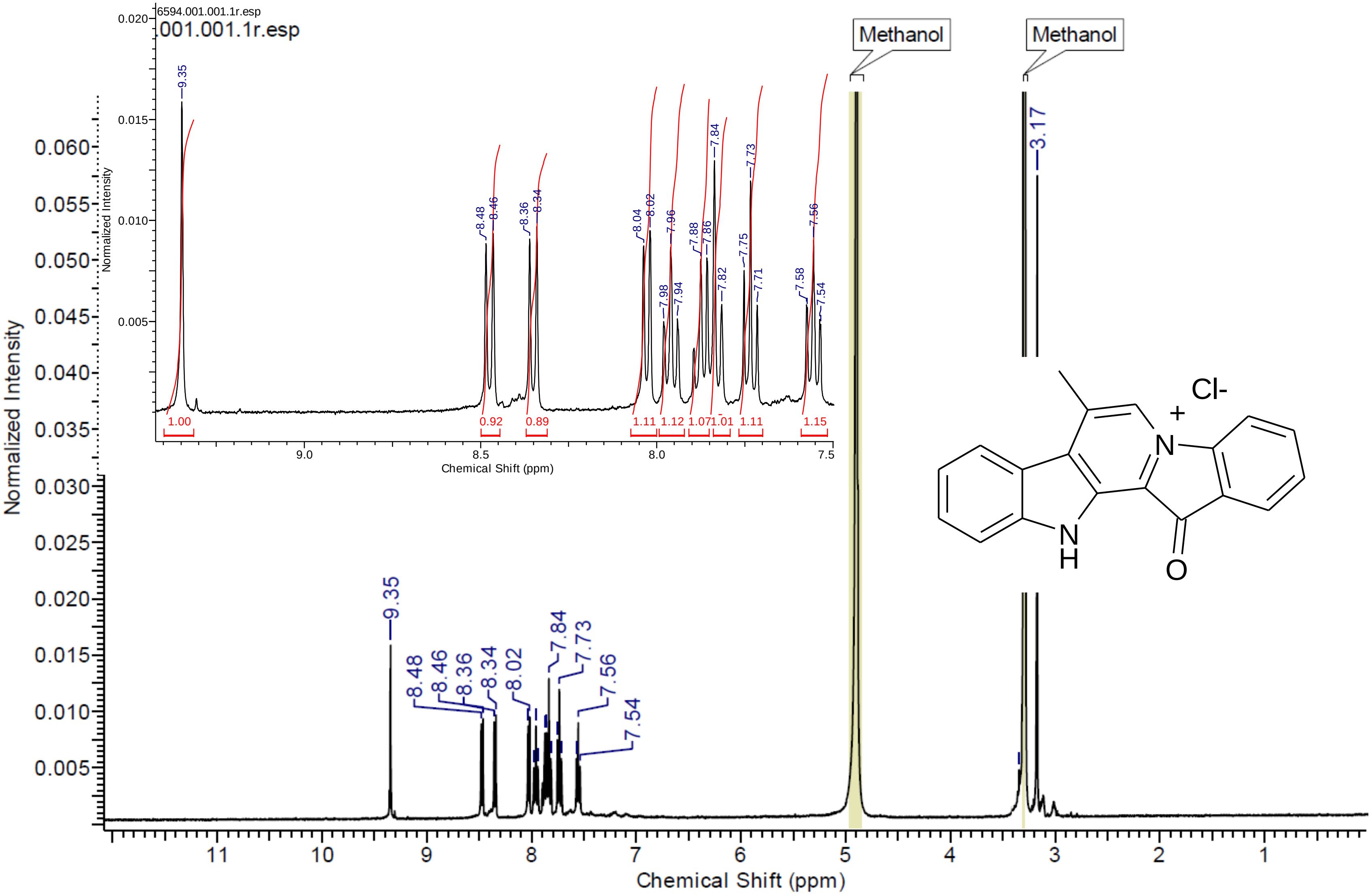

^{13}C NMR spectra of 12,13-dihydro-7-methyl-13-oxopyrido[1,2-*a*:3,4-*b'*]diindol-5-ium chloride

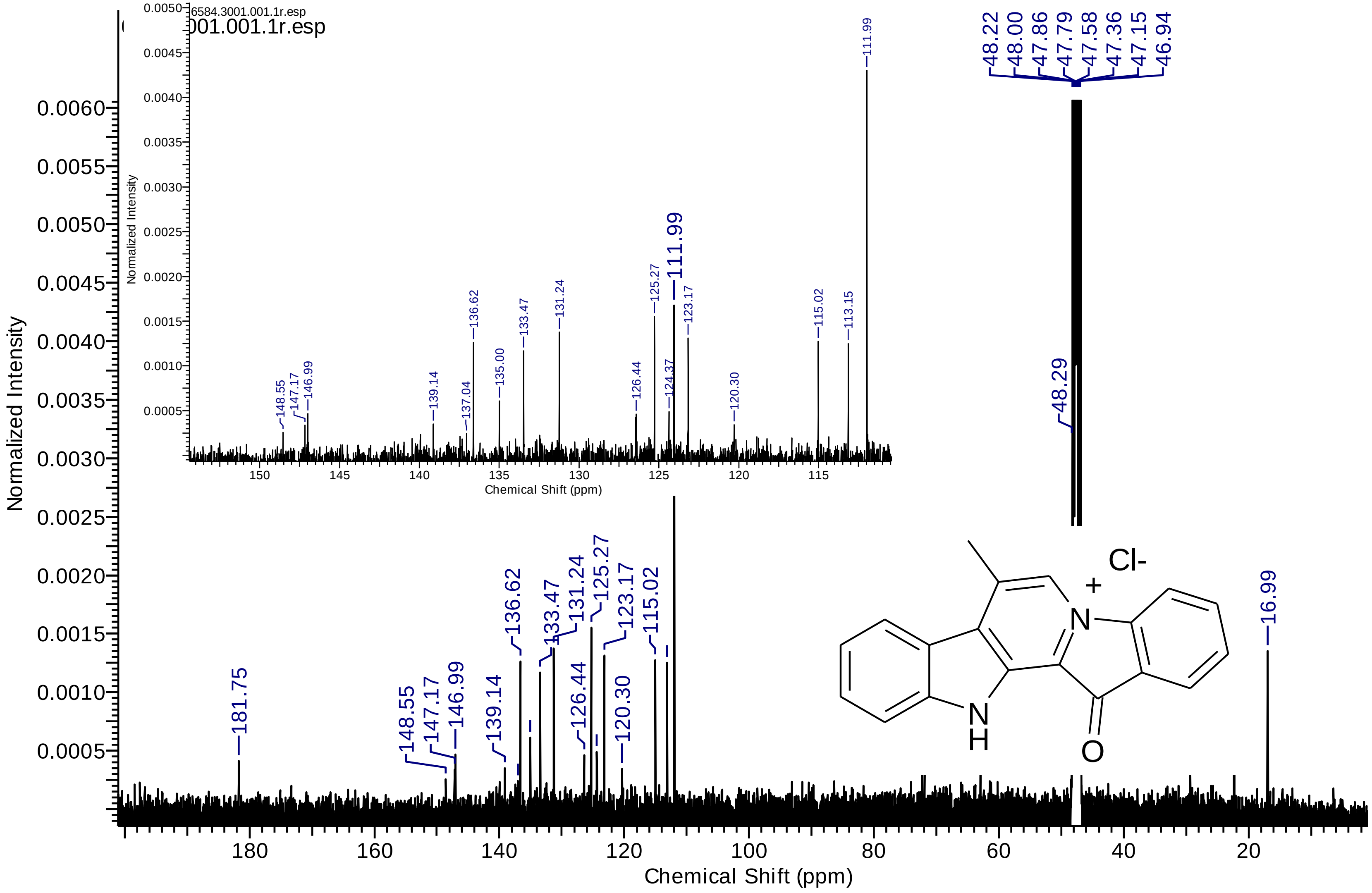

^{1}H NMR spectra of 12,13-dihydro-6-methyl-13-oxopyrido[1,2-*a*:3,4-*b'*]diindol-5-ium chloride

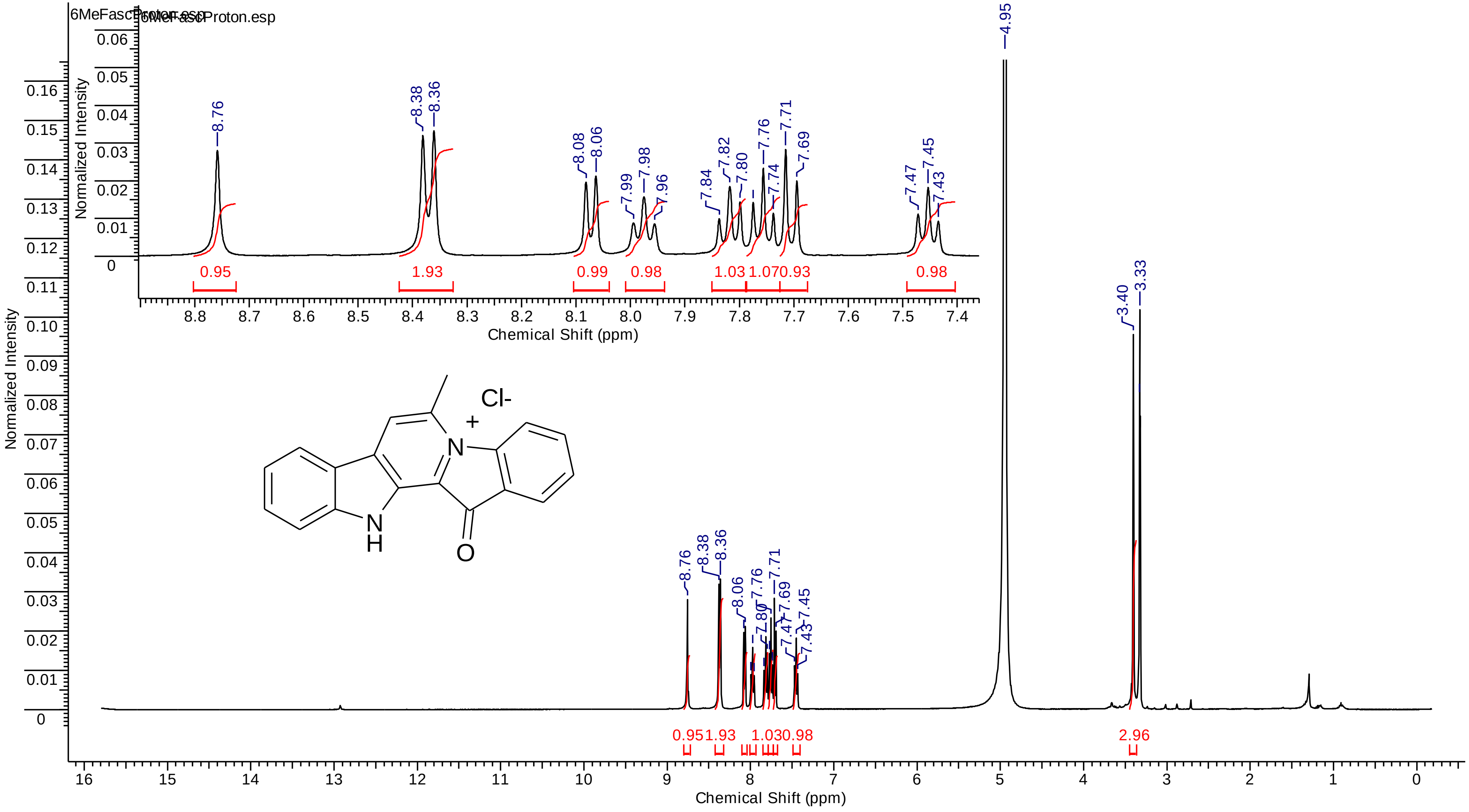

^{13}C NMR spectra of 12,13-dihydro-6-methyl-13-oxopyrido[1,2-*a*:3,4-*b'*]diindol-5-ium chloride

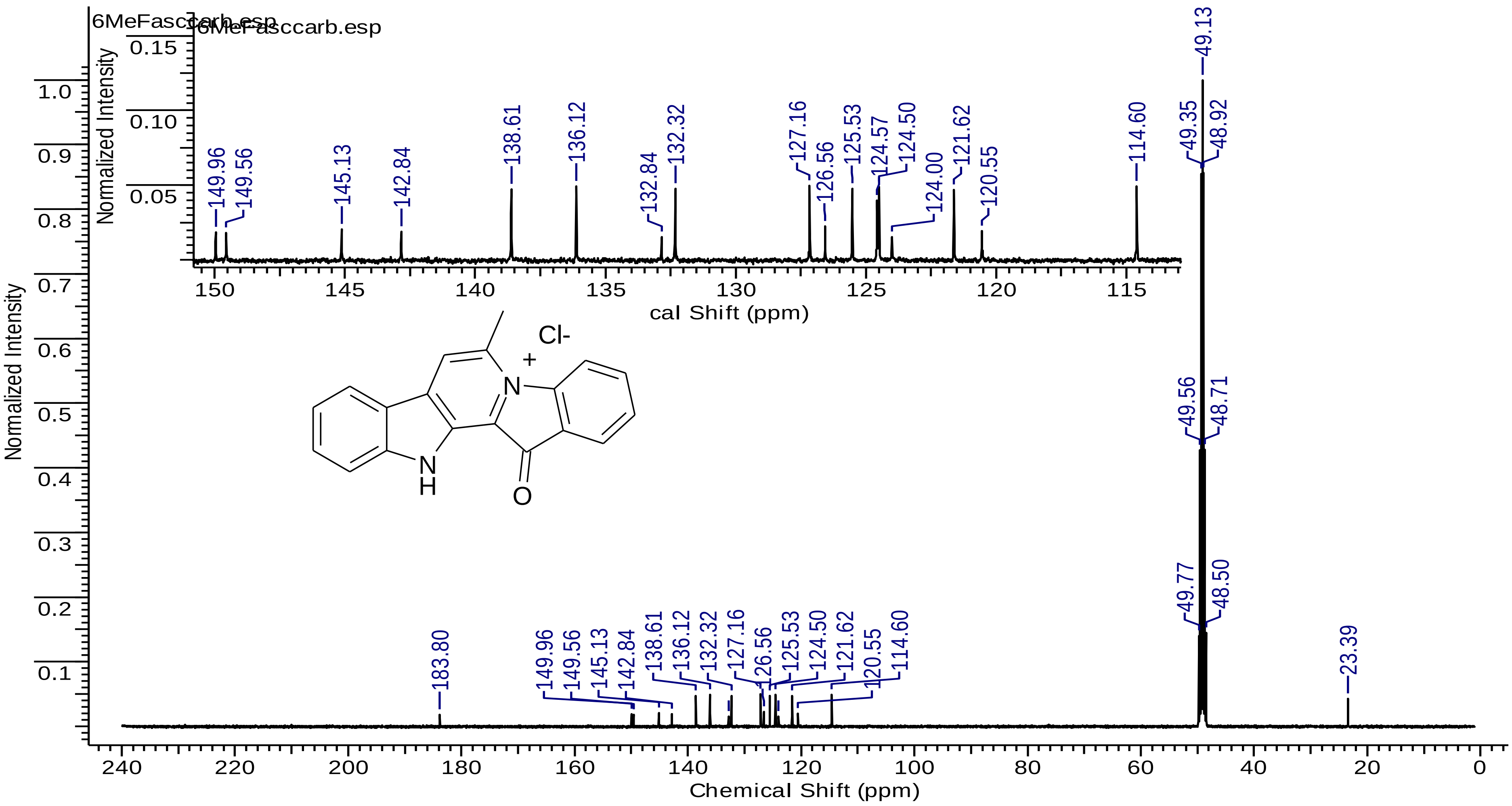

^{1}H NMR spectra of 12,13-dihydro-6-phenyl-13-oxopyrido[1,2-*a*:3,4-*b'*]diindol-5-ium chloride

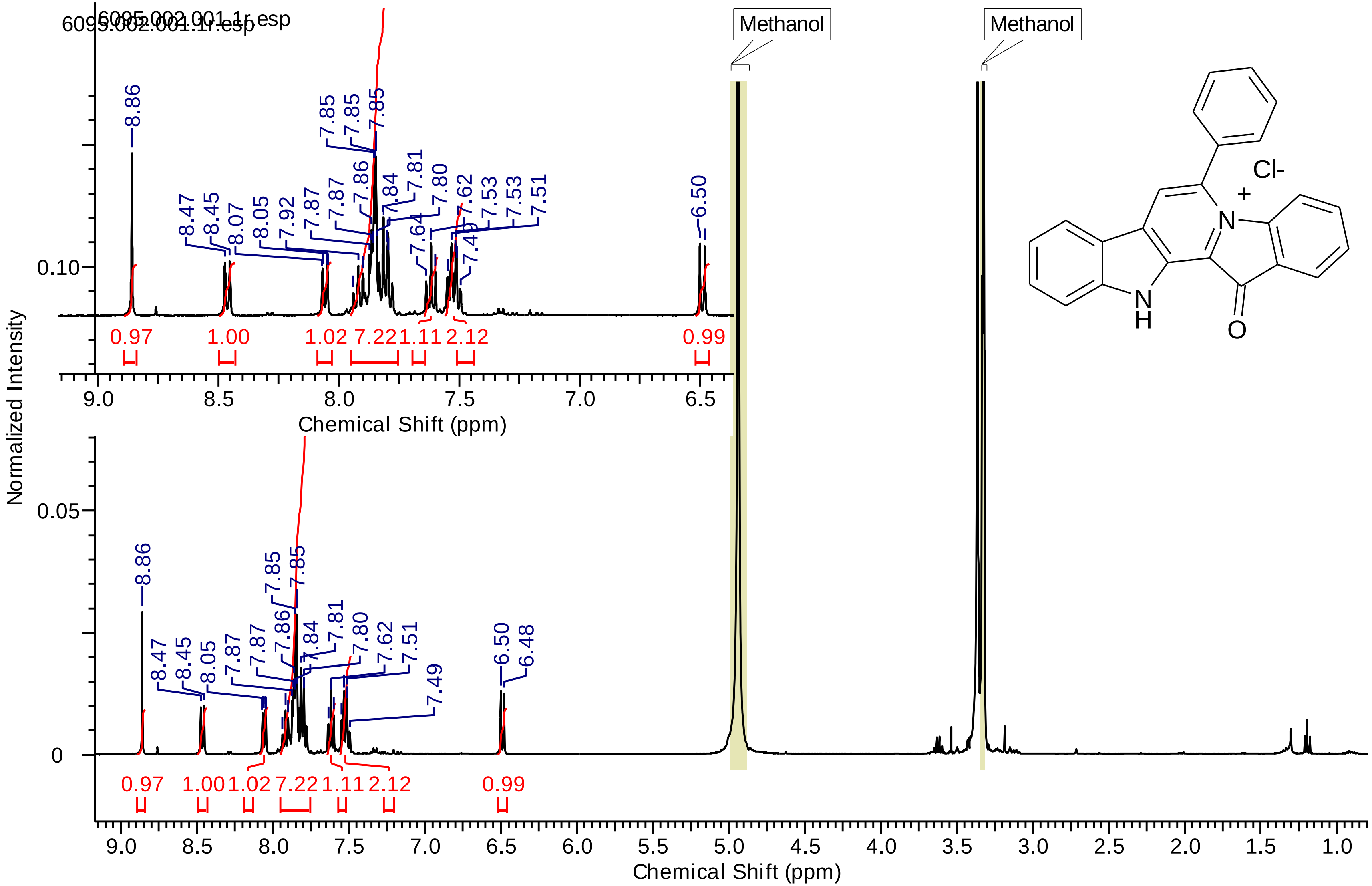

^{13}C NMR spectra of 12,13-dihydro-6-phenyl-13-oxopyrido[1,2-*a*:3,4-*b’*]diindol-5-ium chloride

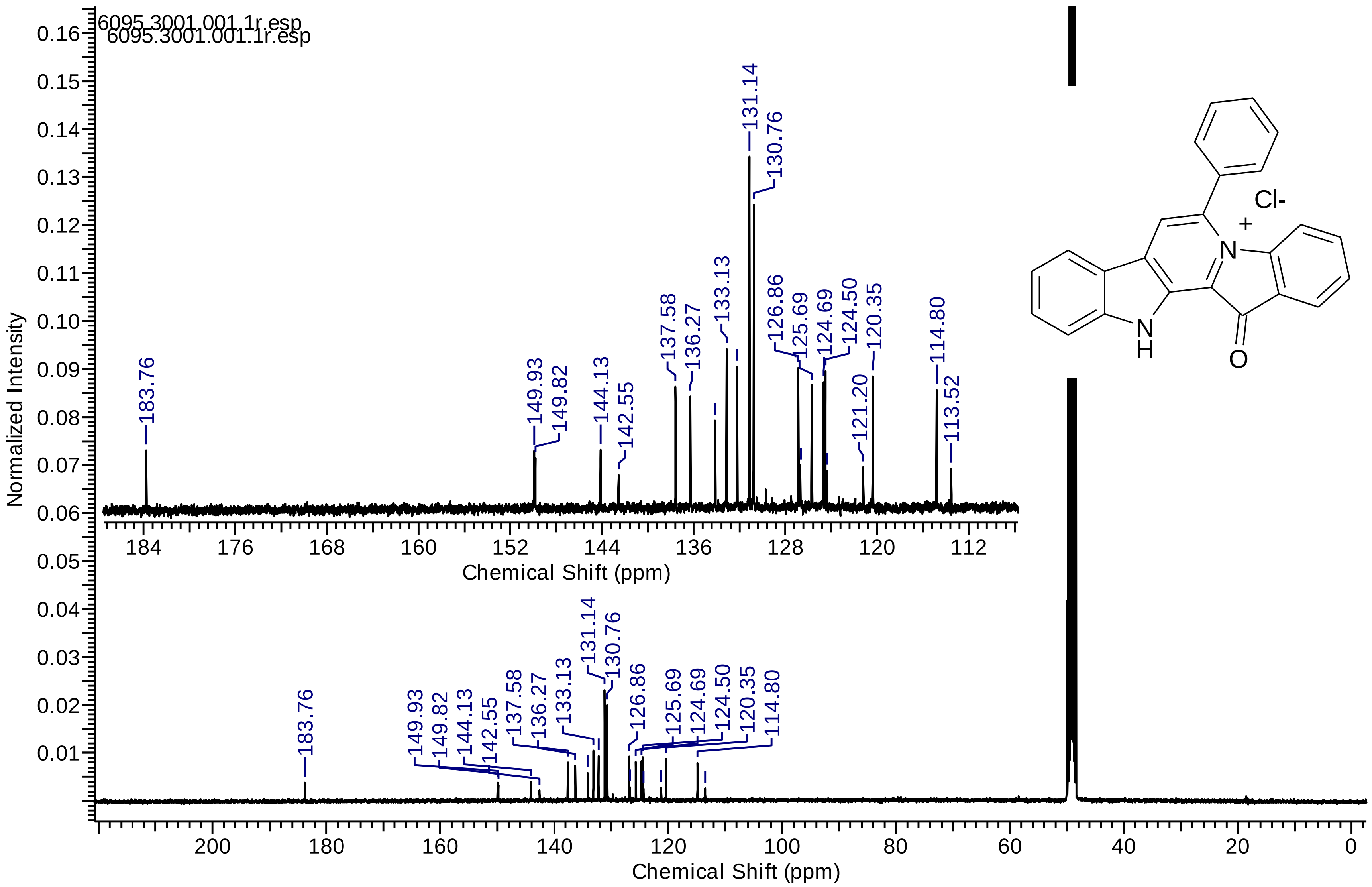

Supplement: Supplementary file 1 [file marinedrugs-20-00185-s001.zip › marinedrugs-1600124-supplementary.pdf]
